# Pearl millet genome sequence provides a resource to improve agronomic traits in arid environments

**DOI:** 10.1038/nbt.3943

**Published:** 2017-09-18

**Authors:** Rajeev K Varshney, Chengcheng Shi, Mahendar Thudi, Cedric Mariac, Jason Wallace, Peng Qi, He Zhang, Yusheng Zhao, Xiyin Wang, Abhishek Rathore, Rakesh K Srivastava, Annapurna Chitikineni, Guangyi Fan, Prasad Bajaj, Somashekhar Punnuri, S K Gupta, Hao Wang, Yong Jiang, Marie Couderc, Mohan A V S K Katta, Dev R Paudel, K D Mungra, Wenbin Chen, Karen R Harris-Shultz, Vanika Garg, Neetin Desai, Dadakhalandar Doddamani, Ndjido Ardo Kane, Joann A Conner, Arindam Ghatak, Palak Chaturvedi, Sabarinath Subramaniam, Om Parkash Yadav, Cécile Berthouly-Salazar, Falalou Hamidou, Jianping Wang, Xinming Liang, Jérémy Clotault, Hari D Upadhyaya, Philippe Cubry, Bénédicte Rhoné, Mame Codou Gueye, Ramanjulu Sunkar, Christian Dupuy, Francesca Sparvoli, Shifeng Cheng, R S Mahala, Bharat Singh, Rattan S Yadav, Eric Lyons, Swapan K Datta, C Tom Hash, Katrien M Devos, Edward Buckler, Jeffrey L Bennetzen, Andrew H Paterson, Peggy Ozias-Akins, Stefania Grando, Jun Wang, Trilochan Mohapatra, Wolfram Weckwerth, Jochen C Reif, Xin Liu, Yves Vigouroux, Xun Xu

**Affiliations:** 1 International Crops Research Institute for the Semi-Arid Tropics (ICRISAT), Hyderabad, Telangana State India; 2 BGI-Shenzhen, Shenzhen, China; 3 Institut de recherche pour le développement (IRD), Montpellier, France; 4 University of Georgia, Athens, Georgia USA; 5 Leibniz Institute of Plant Genetics and Crop Plant Research (IPK), Gatersleben, Germany; 6 Fort Valley State University, Fort Valley, Georgia USA; 7 Cornell University, Ithaca, New York USA; 8 University of Florida, Gainesville, Florida USA; 9 Junagadh Agricultural University, Jamnagar, Gujarat India; 10 United States Department of Agriculture—Agricultural Research Service (USDA-ARS), Tifton, Georgia USA; 11 Department of Ecogenomics and Systems Biology, University of Vienna, Vienna, Austria; 12 Amity University, Mumbai, Maharashtra India; 13 Institut Sénégalais de Recherches Agricoles (ISRA), Dakar, Senegal; 14 University of Georgia, Tifton, Georgia USA; 15 School of Bioinformatics and Biotechnology, D.Y. Patil University, Mumbai, Maharashtra India; 16 University of Arizona, Tucson, Arizona USA; 17 Phoenix Bioinformatics, Redwood City, California USA; 18 Indian Council of Agricultural Research (ICAR)—Central Arid Zone Research Institute (CAZRI), Jodhpur, Rajasthan India; 19 Laboratoire Mixte International Adaptation des Plantes et Microorganismes Associés aux Stress Environnementaux, Centre de Recherche de Bel Air, Dakar, Senegal; 20 ICRISAT Sahelian Center, Niamey, Niger; 21 Faculty of Sciences and Techniques, University Abdou Moumouni, Niamey, Niger; 22 University of Montpellier, Montpellier, France; 23 Laboratoire de biométrie et Biologie Evolutive, Université Lyon 1, Villeurbanne, France; 24 Oklahoma State University, Stillwater, Oklahoma USA; 25 Institut des Mondes Africains (IMAf), Paris, France; 26 CNR-Consiglio Nazionale delle Ricerche, Istituto di Biologia e Biotecnologia Agraria, Milan, Italy; 27 Pioneer Hi-Bred Private Limited, Hyderabad, Telangana State India; 28 Institute of Biological, Environmental and Rural Sciences, Aberystwyth University, Ceredigion, UK; 29 Visva-Bharati, Santiniketan, West Bengal India; 30 USDA-ARS, Ithaca, New York USA; 31 Indian Council of Agricultural Research (ICAR), New Delhi, India; 32 Vienna Metabolomics Center (VIME), University of Vienna, Vienna, Austria; 33 BGI-Qingdao, Qingdao, China; 34 China National GeneBank (CNGB), Shenzen, China

**Keywords:** Next-generation sequencing, Agricultural genetics

## Abstract

Pearl millet [*Cenchrus americanus* (L.) Morrone] is a staple food for more than 90 million farmers in arid and semi-arid regions of sub-Saharan Africa, India and South Asia. We report the ∼1.79 Gb draft whole genome sequence of reference genotype Tift 23D_2_B_1_-P1-P5, which contains an estimated 38,579 genes. We highlight the substantial enrichment for wax biosynthesis genes, which may contribute to heat and drought tolerance in this crop. We resequenced and analyzed 994 pearl millet lines, enabling insights into population structure, genetic diversity and domestication. We use these resequencing data to establish marker trait associations for genomic selection, to define heterotic pools, and to predict hybrid performance. We believe that these resources should empower researchers and breeders to improve this important staple crop.

**Supplementary information:**

The online version of this article (doi:10.1038/nbt.3943) contains supplementary material, which is available to authorized users.

## Abstract (web summary)

Draft genome, 994 re-sequenced lines and GWAS for yield-traits provide a resource of genetics and genomics tools for pearl millet researchers and breeders.

**Supplementary information:**

The online version of this article (doi:10.1038/nbt.3943) contains supplementary material, which is available to authorized users.

## Main

Global temperatures are expected to increase from 1 to 6 °C by 2100, with serious consequences for agriculture^1^. This means that climate-appropriate measures to ensure food security are a priority, especially as the human population is projected to reach 9.1 billion by 2050^2^. Crops that are adapted to the predicted environmental changes have been proposed as one solution^3^. Even now, availability and further improvement of crops that can withstand climate change could reduce the hunger of the 805 million undernourished people living mainly in developing countries^4^.

Pearl millet (*Pennisetum glaucum* (L.) R. Br., syn. *Cenchrus americanus* (L.) Morrone), a C4 grass, is a highly cross-pollinated diploid (2*n* = 2*x* = 14) with excellent photosynthetic efficiency and biomass production potential. It is cultivated as a staple food grain and source of straw for fodder and fuel in arid and semi-arid regions of sub-Saharan Africa, India and South Asia. Climate-smart vegetative, reproductive, and physiological features of pearl millet make this crop well-suited to growth in harsh conditions including low soil fertility, high soil pH, high soil Al^3+^ saturation, low soil moisture, high temperature, high salinity and limited rainfall. Pearl millet reliably produces grain in regions that have a mean annual precipitation as low as 250 mm. In the same drought conditions maize (*Zea mays*), rice (*Oryza sativa*), sorghum (*Sorghum bicolor*), bread wheat (*Triticum aestivum*) and durum wheat (*Triticum durum*) are likely to fail^5^.

Pearl millet is cultivated on ∼27 million hectares worldwide and is the staple food for more than 90 million farmers living in poverty. Millet grain is highly nutritious, with 8–19% protein, low starch, high fiber (1.2 g/100 g)^6^, and higher micronutrient concentrations (iron and zinc) than rice, wheat, maize and sorghum^7^. Importantly, the potential of this crop to tolerate air temperatures >42 °C during the reproductive phase means that it can be cultivated using irrigation in the very hot summers of northwestern India^8^.

Despite the clear importance of pearl millet in agriculture, the production and productivity of this staple crop are very low, with an average grain yield of just 900 kg/ha. This is because pearl millet is mainly grown in dryland conditions, which are marginal production environments, and with minimal use of commercial inputs, such as, adequate irrigation, fertilizers and pesticides. Genetic gains, the rate of increase in yield over a given time period, during 1996–2013 in pearl millet have averaged around 24 kg of grain/ha/year in India, which has the highest millet productivity and production of the main pearl millet growing countries^9^. Pearl millet is vulnerable to several foliar diseases including downy mildew (caused by *Sclerospora graminicola*), Pyricularia leaf spot or blast (*Pyricularia grisea*), and rust (*Puccinia substriata* var. *indica*). Indeed, these pathogen infections can result in massive yield losses and reduced fodder quality. A limited range of genomics tools for pearl millet have impeded the ability of researchers and breeders to exploit methods for improvement, until now.

To accelerate pearl millet crop improvement, we sequenced the whole genome of reference genotype Tift 23D_2_B_1_-P1-P5. We also resequenced 994 pearl millet genotypes, including 963 inbred lines and single plants from each of 31 wild accessions, in order to understand the population structure, genetic diversity and domestication of this staple crop. We carried out a genome-wide association study (GWAS) to predict yield-associated traits in both irrigated and drought conditions. We also used genomic prediction to predict hybrid performance. These applications highlight the utility of our resequencing data set for accelerating breeding and enhancement of genetic gains in pearl millet.

## Results

### Genome assembly

To assemble the pearl millet genome, we used whole genome shotgun (WGS) and bacterial artificial chromosome (BAC) sequencing. Ten small inserts (of ∼170, 250, 500 and 800 bp), and 13 large inserts (of ∼2, 5, 10, 20 and 40 kb) WGS libraries were constructed using Tift 23D_2_B_1_-P1-P5^10^ genotype. These libraries were sequenced on the Illumina HiSeq 2000 and 520 Gb of sequence data, representing 296× genome coverage, were produced (Supplementary Table 1). Two BAC libraries, with an average insert size of ∼120 kb, were constructed from Tift 23D_2_B_1_-P1-P5 using EcoRI and HindIII. 972 Gb of sequence data were generated from 100,608 BAC clones at ∼80× genome coverage (Supplementary Table 2 and Supplementary Fig. 1). In brief, 1.49 Tb of sequence data, after stringent filtering and correction steps, were assembled into 1.58 Gb of contigs (sequences without gaps or Ns) and 1.82 Gb of scaffolds (contigs joined with estimated gaps filled in).

Based on k-mer statistics, the pearl millet genome size was estimated to be 1.76 Gb (Supplementary Fig. 2), indicating that ∼90% of the genome was assembled. Scaffolds longer than 1 kb totaled 1.79 Gb, with 50% of scaffolds (N50) being longer than 884.95 kb (N50 contig = 18,180 bp) and the largest scaffold spanning 4.82 Mb (Supplementary Table 3). To evaluate the assembly, we generated additional whole genome sequence data with 1× coverage on the PacBio platform. More than 90% of these long reads were mapped back to a scaffold with more than 90% similarity and 90% ratio of aligned length (Supplementary Fig. 3).

**Figure 1:**
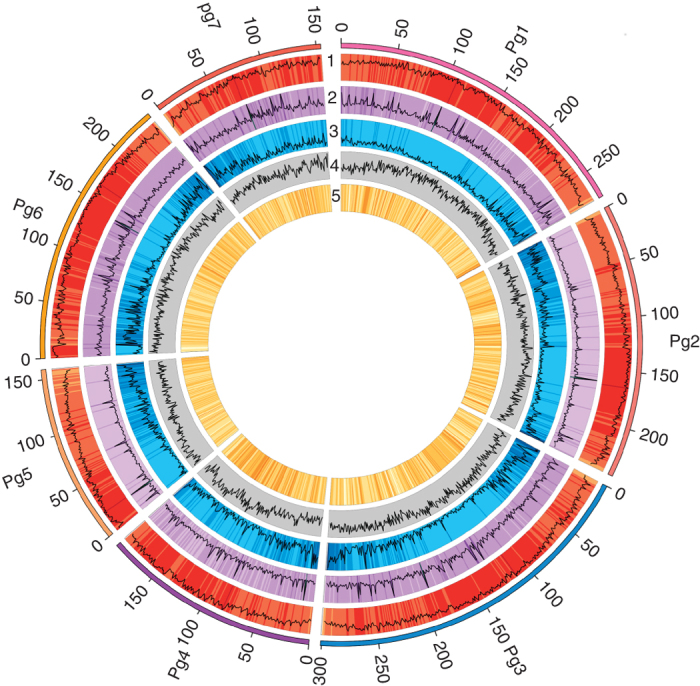
Pearl millet genome. Genome features in 1-Mb intervals across the seven pseudomolecules. Units on the circumference are megabase values of pseudomolecules. (1) Repeat density, (2) tandem repeat density, (3) gene density, (4) GC content and (5) SNPs identified by resequencing PMiGAP lines in 1-Mb bins. The genome assembly furnished an average GC content of 47.9% and contained 38,579 gene models with mean coding sequence length of 1,014.71 bp.

Linkage information from three biparental mapping populations, and collinearity with the genome of foxtail millet (*Setaria italica*)^11^ were used to assemble genomic scaffolds into pseudomolecules. We assembled 1.56 Gb into seven pseudomolecules (Pg1 to Pg7, Fig. 1 and Supplementary Table 4). The average GC content of pearl millet (47.9%) is higher than that of foxtail millet (46.1%), sorghum (44.5%), barley (*Hordeum vulgare*, 44.4%), and rice (43.5%) (Supplementary Fig. 4). We assessed the variability in GC content in 10-kb non-overlapping sliding windows (Supplementary Fig. 5) to show that the observed GC content did not arise from sequencing-based GC bias. The GC content in whole genome coding sequence (CDS; 54.76%) and in 384 expanded gene families (53.14%) was examined as well; it was at a similar proportion to the total genome, providing confidence in this result (Supplementary Table 5 and Supplementary Fig. 6). Analysis of completeness was carried out using the core eukaryotic gene mapping approach (CEGMA), which revealed that >97% of genes were present in the assembly (Supplementary Table 6).

### Repetitive sequences

In total, 1.22 Gb of repeat elements were identified in a 1.58-Gb genome assembly, indicating that 77.2% of the assembled genome is repetitive. In addition, because the repetitive parts of the genome are always the parts that are under-represented in the genome assembly, most of the unassembled DNA (0.18 Gb) is most likely repetitive, too. This is not surprising, because multiple repeats will often collapse into a single repeat in an assembly and also because “repeat masking” is often performed before some assembly steps^11,12,13^. We expect the true percentage of repetitive DNA to be a minimum of 80%. This is similar to the proportion of repetitive DNA found in the 2.3-Gb maize genome (>85%), and considerably more than in 730-Mb sorghum^14^ (∼61%), ∼400-Mb foxtail millet^11^ (∼46%) or 466-Mb rice^15^ (∼42%) genomes. In common with the pattern in many other plant genomes, long-terminal repeat (LTR) retrotransposons were the most abundant class of repetitive DNA, and comprise >50% of the nuclear genome of pearl millet (Supplementary Table 7). Using RepeatMasker, we found that sequence divergence rates were high (peak at 28%) among long interspersed nuclear elements (Supplementary Fig. 7).

### Genes and annotation

A total of 69,398 transcriptome assembled contigs (TACs), amounting to 43 Mb in total, were identified using pearl millet transcriptome sequences from two different studies^16,17^ and a new pearl millet transcriptome assembly generated for this study (Supplementary Table 8). *Ab initio* homology-based gene prediction were combined with transcript assembly to infer a non-redundant set of 38,579 gene models with an average transcript size of 2,420 bp and an average coding sequence of 1,014 bp (Table 1; Supplementary Table 9). The average lengths of mRNA, CDS, introns and exons in pearl millet were similar to those reported for other cereal genomes (Supplementary Fig. 8). Among 458 of the most conserved genes in CEGMA, 437 (95.4%) genes were complete but 8 (1.7%) genes were not found in the genome sequence, 8 (1.7%) genes were not included in the gene set, and 5 (1.1%) genes had more than one copy (possibly fragmented genes). In addition, for 956 genes in benchmarking universal single-copy orthologs (BUSCO) analysis, we annotated 96.7% genes, and 95.4% of these are complete. Gene models of rice and *Arabidopsis thaliana* have been annotated and carefully validated. We chose to use the gene models of rice, which is more closely related to pearl millet than *A. thaliana*, to investigate the completeness of pearl millet genes. Of the 4,202 single-copy genes in rice, 90.86% have homologs in pearl millet, and 86% of these pearl millet genes were complete when compared with rice gene models (ratio of pearl millet length/rice length 0.8), reflecting the completeness of single-copy genes. Gene density increased toward the ends of pseudomolecules (Fig. 1), consistent with findings in all other cereal genomes published to date^11,14,15^. Most of the annotated genes coded for proteins with homology to proteins in SwissProt^18^ (55.61%) and InterPro (ref. 19) (65.53%). Functions were assigned to 27,893 (72.30%) genes, leaving 10,686 (27.70%) genes unannotated (Supplementary Table 10).

**Table 1:** Statistics of genome assembly

	All scaffolds (≥1K)	Scaffold ≥ 2K
**Assembly features**		
Number of scaffolds	25,241	10,605
Total span	1,793,241,529 bp	1,773,407,327 bp
N50 (scaffolds)	884,945 bp	893,809 bp
Longest scaffold	4,816,714 bp	4,816,714 bp
Number of contigs	175,708	160,430
Total length of contigs	1,556,180,121 bp	1,536,443,592 bp
Longest contig	282,901 bp	282,901 bp
N50 (contigs)	18,180 bp	18,442 bp
GC content	47.90%	47.88%
**Gene models**		
Number of gene models	38,579	
Number of gene models (without transposable elements)	38,542	
Mean transcript length	2,420.19 bp	
Mean coding sequence length	1,014.71 bp	
Mean number of exons per gene	4.09	
Mean exon length	248.06 bp	
Mean intron length	454.77 bp	
Number of genes annotated	29,344 (76.06%)	
Number of genes unannotated	9,235 (23.94%)	
**Non-protein coding genes**		
Number of miRNA genes	183	
Mean length of miRNA genes	125.51 bp	
miRNA genes share in genome	0.001%	
Number of rRNA fragments	235	
Mean length of rRNA fragments	265.70 bp	
rRNA fragments share in genome	0.003%	
Number of tRNA genes	909	
Mean length of tRNA genes	75.86 bp	
tRNA genes share in genome	0.004%	
Number of snRNA genes	752	
Mean length of snRNA genes	119.04 bp	
snRNA genes share in genome	0.005%	

Predicted pearl millet proteins were compared to those already annotated in ten plant species (*Arabidopsis*^20^, Brachypodium (*Brachypodium distachyon*)^21^, banana (*Musa acuminata*)^22^, barley^23^, foxtail millet^11^, maize^24^, rice^15^, sorghum^14^, soybean (*Glycine max*)^25^ and bread wheat)^26^ and, as expected according to evolutionary relatedness, the highest number of orthologs were identified in foxtail millet (74.16%) and the lowest number in *Arabidopsis* (61.88%; Supplementary Table 11). Reciprocal pairwise comparisons of predicted proteins for 38,579 pearl millet gene models with 385,891 gene models from the same ten plant species (as above) identified 17,949 orthologous groups (Supplementary Table 12), of which 5,232 contained only a single pearl millet gene, which is suggestive of simple orthology (Supplementary Table 13; Supplementary Fig. 9). In addition to protein-coding genes, we predicted 909 tRNA, 235 rRNA, 183 microRNA (miRNA) and 752 small nuclear RNA (snRNA) genes in our assembly (Supplementary Table 14).

### Gene families

**Figure 2:**
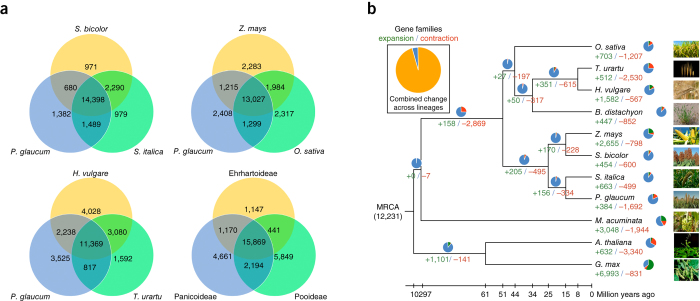
Gene conservation and gene family expansion and contraction in pearl millet. (**a**) Venn diagrams show the number of genes shared between different grass species and among grass families; pearl millet shares 14,398 genes with sorghum and foxtail millet; 13,027 genes with maize and rice; 11,369 genes with barley and wheat. (**b**) 384 gene families are substantially expanded and 1,692 gene families are contracted in pearl millet compared with other plant genomes.

We identified unique and shared gene families among different species in the grass subfamilies Panicoideae, Pooideae and Ehrhartoideae using OrthoMCL (Ortho Markov Cluster Algorithm http://orthomcl.org/orthomcl/)^27^. Pearl millet and foxtail millet share 15,887 gene families (of those, 14,398 are also found in sorghum) while pearl millet and barley share 13,607 gene families (Fig. 2a). A total of 15,869 gene families are present in at least one species in each of the three subfamilies (i.e., Panicoideae, Ehrhartoideae and Pooideae) analyzed (Fig. 2a). 354 gene families were substantially expanded in pearl millet and 1,692 gene families were contracted (Fig. 2b). We compared the average length of the genes for the 384 expanded gene families among all the ten species and used “Quantile” statistics concept to estimate the short CDS. In this concept, Q_1_ is “25th percentile”, Q_3_ is “75th percentile” and interquartile range (IQR) is estimated as Q_3_–Q_1_. We consider a length shorter than Q_1_–3(IQR) to be an extreme outlier. By using this method, we found that only 24 (6.25%) genes had substantially shorter CDS in pearl millet genes compared to other species. Thus, only a small proportion of the expanded gene families might be misidentified because of possible partial genes (Supplementary Fig. 10).

Expansion and contraction of gene families between species might also highlight differences in bioinformatics analysis carried out for different genomes. Bias in gene model identification among different studies might render a comparison of expansion or contraction challenging. One potential source of bias is if a gene is split, that is, a complete gene is instead annotated as two separate genes. Based on eukaryotic orthologous gene sequences, we estimate that 2.3% of our genes might have been misannotated in this way (Supplementary Table 6). Although we found that 1,692 families were contracted in pearl millet, contraction is 5.4 times more likely than expansion. One explanation may be that there was a far higher proportion of split genes in the reference genomes of the other species that we use for comparison than in our pearl millet assembly. This would make our number of gene family contractions an overestimate.

Gene families that seem to be the most greatly expanded are those encoding cutin, suberin, wax biosynthetic genes (*P* < 10^−6^) and transmembrane transporters of secondary metabolites (ABC transporters, *P* < 10^−24^) (Supplementary Table 15). Triterpenoids are a component of wax, and we also observed a substantial expansion of the gene families associated with terpenoid backbone biosynthesis, and monoterpenoid (*P* < 0.05) and di-terpenoid biosynthesis (*P* < 0.005). Notably, increased cuticular wax synthesis improves drought tolerance in *Arabidopsis* species^28^, while reduced wax production has been associated with drought sensitivity in rice^29^. An enriched repertoire of genes for lipid synthesis and export of macromolecules in pearl millet might contribute to its heat and drought tolerance.

Resistance to pathogens is a crucial contributor to crop yield. The majority of resistance genes in plants contain a nucleotide binding site (NBS). Identification of NBS-containing genes in pearl millet will help to identify putative resistance genes. 378 NBS-encoding genes were manually verified after initial searching, comprising ∼1% of the total gene set, similar to the proportion found in other cereal genomes (Supplementary Table 16). NBS-leucine rich repeats (NBS-LRR) genes made up ∼43% of the NBS-genes, with NBS-only genes comprising ∼41%. Of the 378 NBS-encoding genes, 360 were mapped to one of the seven pseudomolecules, with significantly (Chi-squared test *P*-value < 10^−10^) biased distribution among the pseudomolecules; ∼26.2% and ∼25.7% were located on Pg4 and Pg1, respectively (Supplementary Table 17). These are also the same two pseudomolecules to which a downy mildew resistance quantitative trait locus (QTL) was mapped^30^. We observed large tandem arrays of NBS genes near the telomere region of Pg1 (two 4-gene groups, four 5-gene groups and one 6-gene groups) followed by Pg4 (three 2-gene groups and two 4-gene groups) (Supplementary Fig. 11 and Supplementary Table 18), consistent with a biased distribution of these loci and suggesting that tandem duplication may be an important source of local gene amplification.

### Population structure, diversity and domestication

To better elucidate population structure, assess genetic diversity and understand pearl millet domestication, we resequenced 994 lines. The lines resequenced comprised 260 inbred male sterility maintainer (B-) and 320 male fertility restorer (R-) lines, 345 Pearl Millet Inbred Germplasm Association Panel (PMiGAP) lines (including cultivated germplasm from Africa and Asia, elite improved open-pollinated cultivars, hybrid parental inbreds and inbred mapping population parents)^31^, 38 inbred parents of mapping populations and 31 wild accessions. We generated a total of 1.16 Tb whole-genome resequencing (WGRS) data with 1.68× coverage (∼3.05 Gb per line) on PMiGAP lines and a total of 116 Gb WGRS data with 1.86× coverage (∼3.38 Gb per line) on parental lines of mapping populations (Supplementary Tables 19 and 20). In addition, for PMiGAP lines, 78.9 Gb of data at an average coverage of 0.12× was generated using genotyping by sequencing^32^, while for B- and R-lines, 614.45 Gb of data at 0.59× coverage with an average of 1.06 Gb per sample was generated using RAD sequencing^33^ (Supplementary Table 21). Single plants from each of 31 wild accessions sampling the Sahel from Senegal to Sudan were resequenced at an average 2× coverage using WGRS approach (Supplementary Table 22).

**Figure 3:**
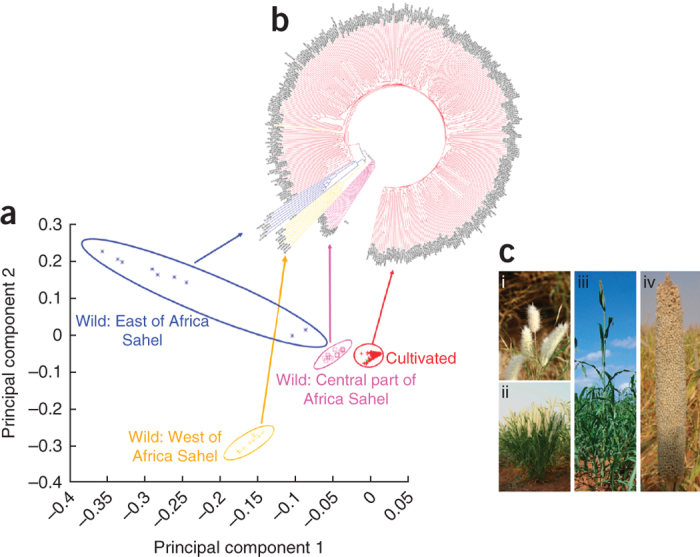
Domestication and genetic diversity in elite and wild accessions of pearl millet. (**a**) Principal component analysis of 376 pearl millet lines (345 PMiGAP lines and 31 wild accessions) using 450,000 high-quality SNPs. Four different groups were identified: cultivated lines (red) and wild lines from east (blue), west (orange) and central Africa (pink). (**b**) Neighbor joining (NJ) tree based on 450,000 high-quality SNPs. This analysis also identified separate groups of cultivated and wild lines from east, west and central parts of Africa. (**c**) Morphological differences between wild (i, ii) and cultivated accessions (iii, iv) of pearl millet. Wild accessions have numerous bristled spikes in the inflorescence and low seed density (i), and a plant architecture characterized by numerous basal and aerial branches (ii), with a plant height of around 1 m. Cultivated accessions have exposed seeds and a high seed density per spike (iii), with a few basal branches and no aerial branches (iv).

We identified 88,256 simple sequence repeat (SSR) motifs using the *MI*cro*SA*tellite program^34^ in the pearl millet genome sequence and designed primers for 74,891 SSR-containing sequences (Supplementary Tables 23 and 24), which can be used by the pearl millet community for genetics and breeding applications. Based on resequencing data, we identified 29,542,173 single-nucleotide polymorphisms (SNPs) in PMiGAP lines (Supplementary Table 25 and details for parents of mapping populations and hybrid parental lines Supplementary Tables 26,27,28), 3,844,446 insertions and deletions shorter than 50 bp (Supplementary Tables 29,30,31), and 423,118 genome-wide structural variations larger than 50 bp such as deletions, duplications and insertions (Supplementary Table 32 and Supplementary Figs. 12–15). We conducted a principal component analysis (PCA) and constructed a neighbor-joining tree based on 450,000 high-quality SNPs. The PCA analysis and phylogenetic tree showed four main clusters, three that contained wild accessions and one that grouped together the cultivated germplasm (Fig. 3a,b). The three wild accession clusters were separated by geographical origin into East, Central and West African clusters (Fig. 3a,b).

The closest of the wild groups to the cultivated samples is from the central part of West Africa (Fig. 3b), indicating that pearl millet originated in this region, consistent with prior research^35^. The oldest archaeological remains, which date to 4,500 years ago, were found in the north-central Sahel, in accordance with our genetic analyses^36^. Studies of archaeological remains found that by 3,500 years ago cultivation of pearl millet was widespread in Sahelian Africa^37,38,39^. Spread of pearl millet agriculture to Asia, and in particular to India also dates to 3,500 years ago^40^. Average pairwise nucleotide diversity within populations (θ_π_) and Watterson's estimator of segregating sites (θ_**ω**_) both indicated high diversity among wild accessions (average θ_π_ = 0.00366 and θ_**ω**_ = 0.00342) compared with PMiGAP (average θ_π_ = 0.00238 and θ_**ω**_ = 0.00289) on all seven pseudomolecules (Supplementary Table 33). In agreement with the PCA analysis and neighbor-joining tree, we observed strong population structure in the wild accessions and weak population structure in PMiGAP lines (Supplementary Figs. 16 and 17). The weak cultivated pearl millet structure suggests a homogenous genetic diversity across large geographical scale. This pattern is certainly associated with a rapid spread of pearl millet agriculture in Africa and India without major bottlenecks during diffusion. This pattern is expected for inbreds derived from a highly allogamous species. The strong structuration of wild diversity and the central geographical origin of the cultivated sample suggest strong untapped and unique diversity for breeding from wild populations found in East Africa (Sudan, Chad) and the West (Senegal, Mauritania).

Domestication in pearl millet, like that observed in maize^24^, was associated with profound modifications of spike morphology and plant architecture (Fig. 3c). We found several genomic regions that showed reduced diversity in the cultivated (but not wild) species that may harbor genes selected for during domestication. Using a negative log ratio of diversity between cultivated (red) and wild (blue) samples, values close to 1 indicate a tenfold decrease in diversity whereas values close to 0 indicate that diversity is maintained in the cultivated samples. We also identified regions with an excess of differentiation based on a fixation index (F_ST_) measure (Supplementary Fig. 18). These analyses provided orthogonal and consistent results and identified 140 genomic regions with values above the 95% threshold for both loss of diversity and differentiation. Using a stringent threshold of 99.5%, and considering only values identified by both statistics, 24 genomic regions had reduced diversity in the cultivated germplasm, of which eight were located on Pg7, six on Pg6 and five on Pg1 (Supplementary Tables 34 and 35). Linkage groups 6 and 7 have previously been identified as carrying QTL that explain most phenotypic differences between wild and cultivated pearl millet germplasm^41,42^. Most of the identified regions have negative Tajima's D values (<−2.0), suggesting a signature of positive selection (Supplementary Table 34). One striking case of diversity loss of more than tenfold was associated with the regulation of an auxin-induced gene PINOID on Pg6. This gene is known as *barren inflorescence2* (ref. 43) in maize, and variation in this gene has been associated with phenotypic variation of the inflorescence^44^. Our analyses also pinpointed genes encoding protiens that might be associated with morphogenesis (LIM2 and PINOID on Pg6, Myosin 11 on Pg7) or gene regulation (Basic helix–loop–helix, bHLH110 on Pg3, Zinc Finger on Pg6). Validation of the role(s) of each of these genes in domestication will require functional analyses and further phenotype–genotype association analyses using fine-scale QTL approaches.

### GWAS

Genome-wide SNP data were used to compute linkage disequilibrium decay (LDD) in all three germplasm sets. We set the *r*^2^ threshold as 0.2 and observed rapid LDD of less than 0.5 kb in B- and R- lines (48 bp) as well as in PMiGAP lines (84–444 bp) (Supplementary Fig. 19). LDD in pearl millet is on par with that in maize, and we note that both these plants are allogamous^45^. Relatively rapid LDD is expected in sets of lines that represent the variation present in a highly allogamous panmictic population. Grain and stover yield, and its component traits, is of crucial importance in pearl millet and has undergone selection during domestication. We carried out GWAS across 288 test-cross progenies of PMiGAP lines for 20 traits, and identified 1,054 strongly significant marker trait associations (MTAs) for 15 traits (Supplementary Table 36): grain number per panicle (91 MTAs), grains per square meter (75 MTAs), stover dry matter yield (kg ha^-1^; 5 MTAs), fresh stover yield (t ha^-1^; 38 MTAs), tillers per plants (147 MTAs), panicle diameter (cm; 1 MTAs), panicle harvest index (%; 1 MTAs), panicle length (cm, EL; 3 MTAs), panicle yield (kg/ha; 9 MTAs), panicle number (ha^-1^; 246 MTAs), plant population (ha^-1^; 68 MTAs), grain yield (kg/ha; 11 MTAs), grain harvest index (%; 5 MTAs), plant height (cm; 344) and 1000 grain mass (g; 10 MTAs). The MTAs explained 9–27% of phenotypic variation (Supplementary Table 36). Selected markers were found common across stress and year for important traits such as grain number per panicle on Pg1 and Pg5 (Supplementary Fig. 20). These markers might be relevant for pearl millet breeding.

### Genomic prediction of hybrid performance

We applied our resequencing data to carry out genomic selection to predict grain yield for test crosses. Four scenarios of prediction were investigated, namely the performance of grain yield in each of the three environments (control, early stress and late stress) and across environments. We observe high prediction accuracy, measured as the Pearson correlation coefficient between the predicted and observed values, standardized with the square root of the heritability (*h* = 0.78), amounting to 0.6 for the performance across environments. Analyses of this kind have been undertaken for grain yield in other crops using genomic selection^46^. A modelling study recently found that with this level of prediction accuracy, genomic selection could substantially improve selection gain per year^47^.

**Figure 4:**
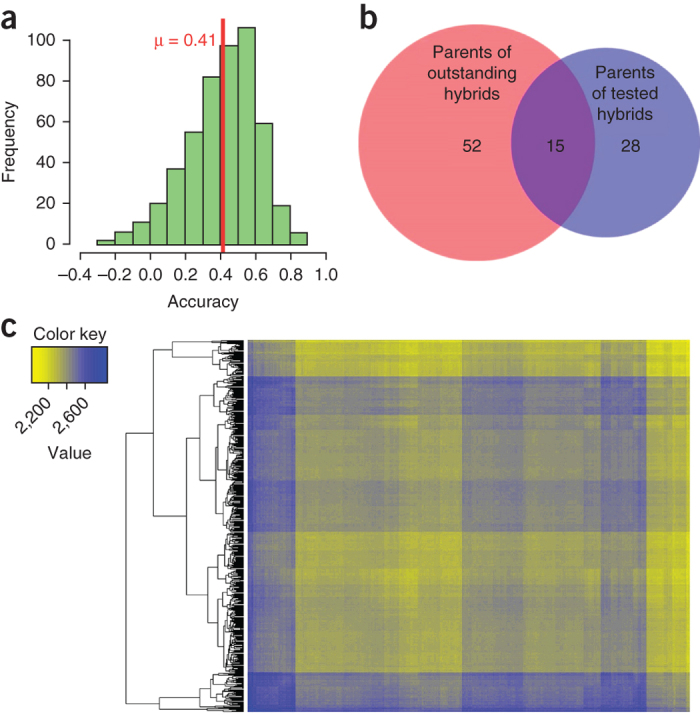
Prediction of hybrid performance. Grain yield of 64 different pearl millet hybrids, produced by crossing 20 male and 23 female lines, was evaluated at five locations (Jamnagar, Anand, SK Nagar, Mahuva, Kothara in India) during 2004–2013. Phenotyping data (Supplementary Data set 1), together with 302,110 high-quality SNP marker data obtained from 580 B and R- lines (Supplementary Table 27), were used to predict hybrid performance. Ridge regression-BLUP, which takes additive and dominance effects into account, was used to predict hybrid performance. (**a**) Prediction accuracy was studied using 500 cross-validation tests. In each cross-validation, 48 hybrids were randomly selected as a training set and the remaining 16 hybrids were used as a test set. (**b**) Promising hybrid combinations that include parental lines that have not been used in breeding efforts previously were identified for testing and release as better hybrids. (**c**) Heat map showing putative heterotic groups.

We also predicted hybrid performance, by using genomic selection strategy that considers additive and dominance effects. The ridge regression best linear unbiased prediction method^46^ was trained using phenotypic grain yield data from 64 pearl millet hybrids grown in five environments in India in replicated trials during the time period 2004–2013. The grain yield data were analyzed with 302,110 SNPs with missing values below 5% and minor allele frequency above 5% for 580 B- and R- lines (Fig. 4a). We found 170 promising hybrid combinations (Supplementary Table 37 and Fig. 4a). Of these, 11 combinations were already used for producing hybrids that showed better performance (Supplementary Table 38). However, 159 combinations have never been used in hybrid breeding (Fig. 4b), and therefore they are good candidates for developing high-yielding hybrids.

We inspected the predicted hybrid performance of all possible 167,910 single-cross combinations by applying hierarchical clustering combined with a heat plot, and examined the potential of this approach to identify promising heterotic groups. The analyses revealed two sets of lines that are predicted to have an average 8% higher hybrid performance when crossed to each other than the total set of 167,910 single-cross combinations (Fig. 4c and Supplementary Fig. 21). These predicted high-yield hybrids could be used as a nucleus to establish high-yielding heterotic groups for hybrid pearl millet breeding^48^ (Supplementary Tables 37 and 38).

## Discussion

Pearl millet is a staple food for more than 90 million people in Africa and Asia. People living in arid and semi-arid regions, in particular, rely on pearl millet, which can crop in the harsh conditions. We sequenced the genome of pearl millet reference genotype Tift 23D_2_B_1_-P1-P5 (available at https://www.ncbi.nlm.nih.gov/assembly/GCA_002174835.1/). The draft genome assembly presents 90% of the pearl millet genome with N50 of scaffolds as 884.95 kb and 87.2% assembled genome into seven pseudomolecules. The genome assembly of cereal species like pearl millet with high levels of repetitive DNA is always challenging. Therefore, in addition to a WGS approach, BAC-sequence data were used to develop the draft genome assembly and PacBio data were generated to validate the assembly. To achieve chromosome level assembly, one can use new approaches of sequencing such as Bionano Genomics optical mapping and Dovetail Genomics chromosome confirmation capture data in different combinations^49^.

Our analysis identified 38,579 protein-coding genes, of which 27,893 (72.30%) were annotated. CEGMA and BUSCO analyses together with comparison with gene models of rice have indicated completeness of predicted genes in pearl millet. Expansion of gene families associated with terpenoid backbone biosynthesis and monoterpenoid and diterpenoid biosynthesis in the genome might explain the high level of heat and drought tolerance in pearl millet as compared to other cereals.

Genome sequence can provide information either about specific genomic regions or specific genes that are associated with agronomically important traits including grain and fodder yield. Pearl millet fodder is the main feedstock for ruminant (and other) livestock, and breeding to improve fodder quality and yield is of crucial importance to both the meat and the dairy industries. In order to identify loci or variants associated with agronomic features, we undertook a large-scale resequencing effort. Resequencing of the PMiGAP set revealed that small structural rearrangements, such as insertions and deletions in the genome have occurred throughout the evolution of pearl millet. This is similar to observations made in maize: a third or more of maize genes seem to be optional. Frequent insertions and deletions pose substantial challenges to resequencing efforts because self-pollinated and small-genome species such as rice are easier to sequence and analyze as compared to cross-pollinated and large-genome species like maize owing to their increased genomic structural variability^50^. With an objective to save the cost, but without losing information, 1.68× coverage WGRS data and 0.12× GBS data were generated on PMiGAP lines and 0.59× coverage RAD-sequencing data were generated on B- and R- lines.

The sequence information from the more genetically diverse PMiGAP inbred panel will be of broader use for genome-wide association mapping and allele mining. All of these sequences are available at https://www.ncbi.nlm.nih.gov//sra/?term=SRP063925. Resequencing data of almost 1,000 pearl millet lines (963 inbreds of cultivated pearl millet and 31 heterozygous wild individuals, available at https://www.ncbi.nlm.nih.gov//sra/?term=SRP063925) provides researchers and breeders with an enormous resource of genome-wide variations including SNPs, indels, SSRs and structural variations (Supplementary Tables 23,24,25,26,27,28,29,30,31,32) for mining alleles of genes with significant MTAs and for developing pearl millet hybrids with increased heterosis. Our analysis on resequencing data on PMiGAP lines together with phenotyping data for 20 traits for GWAS and genomic selection suggests that simultaneous improvement of grain and stover yield might be feasible in pearl millet. Indeed, improved grain and stover yield performance of hybrids in India has been noted over the past 50 years, which underlines the potential for further improvements that could be informed by our analyses.

We also show the use of the genome sequence and resequencing information to make predictions of test-cross hybrid performance. After inspecting predicted hybrid performance of 167,910 single-cross combinations, we identified 159 pair of lines that have not been used so far for hybrid breeding but can exhibit high hybrid performance. This type of analysis has considerable potential for accelerating future rates of selection gain. Our prediction models were also applied to define heterotic pools for pearl millet for South Asia, which could be crucial for increasing the efficiency of hybrid breeding programs in the same region.

Together the draft genome and resequencing data provide a resource for the research community that should enable a better understanding of trait variation and accelerate the genetic improvement of pearl millet. For instance, we identified 1,054 MTAs for 15 agronomic traits that will be useful for pearl millet breeding. Our findings will also contribute to a better understanding of the genetic basis of the exceptional drought and heat tolerance of pearl millet as we have identified expansion of gene families associated with drought and heat tolerance. A detailed understanding of how well pearl millet crops do in hot, arid and semi-arid regions might enable engineering of not only pearl millet but also other cereal crops like rice, maize and wheat, which are currently able to provide only limited produce in arid or semi-arid regions. This is especially important owing to the pressing need for heat- and drought-tolerant cereal crops in the coming years.

## Methods

### Plant material.

The pearl millet genotype Tift23D_2_B_1_-P1-P5 was bred at the Coastal Plain Experiment Station (Tifton, Georgia, USA) by introducing the *d2* dwarfing gene into the genetic background of elite seed parent maintainer line Tift 23B1, and was chosen to generate a draft genome sequence.

Three bi-parental mapping populations were used to develop the genetic map for organizing scaffolds into pseudomolecules. These populations were: (i) a small recombinant inbred line (RIL) population developed at ICRISAT, Patancheru, based on the cross ICMB 841-P3 × ICMB 863B-P2 (MAPPOP1); (ii) a RIL population developed at the Coastal Plain Experiment Station, Tifton, Gerogia (USA) based on Tift 99B × Tift 454 (MAPPOP2); and (iii) an F_2_ population derived from a wild × domestic cross (MAPPOP3) from Institut de Recherche pour le Developpement (IRD) France. 580 B- and R- lines included 200 B- and 200 R- lines from ICRISAT plus 60 B- and 120 R- lines from 5 organizations from India namely Haryana Agricultural University, Hisar, Haryana; Junagadh Agricultural University, Jamnagar, Gujarat; Mahatma Phule Krishi Vidyapeeth, Dhule, Maharashtra; Sri Karan Narendra Agriculture University, Durgapura, Rajasthan; and JK Agri Genetics Ltd., Hyderabad, Telangana, were resequenced using restriction-site-associated DNA (RAD) sequencing (Supplementary Table 39). The PMiGAP lines contains 345 lines: 263 landraces/traditional cultivars, 46 breeding lines, 25 advanced/improved cultivars and 11 accessions with unknown biological status and represents germplasm from 27 countries in two continents (Supplementary Table 40). These 345 accessions were subjected to WGRS. In addition, 38 inbred parents of mapping populations segregating for drought, downy mildew and rust (Supplementary Table 41) and 31 wild accessions representing seven countries (Mali, Mauritania, Senegal, Sudan, Chad, Mali and Niger) were also resequenced using the WGRS approach (Supplementary Table 42).

### Whole genome shotgun sequencing and assembly.

We constructed 10 small insert libraries including 4 with 170 bp insert, 2 with 250 bp insert, 2 with 500 bp inserts and 2 with 800 bp insert, and 13 mate-pair libraries including 4 with 2 kb insert, 4 with 5 kb insert, 2 with 10 kb insert, and 2 with 20 kb insert and 1 with 40 kb insert from pearl millet genotype Tift 23D2B1-P1-P5. To make libraries with ∼170 to ∼800 bp inserts, high quality DNA samples were sheared, end-repaired, and 'A' bases were added to the 3′ end of the DNA fragments to facilitate ligation to adapters. Fragments in the appropriate size range were selected after separation on an agarose gel and amplified using PCR. For mate-pair libraries, a biotinylation reaction was performed after fragmentation and end-repair. Then DNA fragments of the required size were selected and circularized. Circular DNAs were sheared into approximately 400-600 bp fragments, and biotinylated fragments were captured for terminal modification and adaptor ligation to construct libraries. Paired end reads were generated for each library on an Illumina HiSeq 2000 platform.

For BAC library construction, DNA from pearl millet genotype Tift 23D_2_B_1_-P1-P5 was fragmented using HindIII and EcoRI, and then ligated into vector pCC1BAC. The ligations were transformed into *E. coli* DH10b host cells. After DNA isolation from BAC clones, Covaris LE220 system was used to shear DNA into ∼500 bp. Agilent Bravo Automated Liquid Handling Platform and an Agilent BenchCel Microplate Handler were used to construct BACs for sequencing. Then 96-microTUBE plates (Covaris) were used as sample vessels for automated batch processing followed by index adaptor ligation and size selection^51^. Generally, the sizes of the BAC ranged from 80 -180 kb and fragments for sequencing were about 500 bp. In total 100,608 BAC clones were constructed and HiSeq 2000 was used for sequencing paired end reads of each BAC clone.

For each library, we filtered the reads that comprised more than 5 percent of “Ns” or polyA structure, and also removed reads that possessed 20 or more bases with quality score less than or equal to 7. Reads with >10 bp aligned to the adaptor sequence (allowing ≤3 bp mismatch) were considered as adaptor contaminants and removed. Additionally, paired-end reads with a total length smaller than the library insert size allowing a window of 30 bp were removed. We also trimmed the reads if the quality of bases at the head or tail of the reads was low.

### k-mer analysis.

We performed k-mer analysis^52^ for the estimation of the genome size of pearl millet genotype Tift 23D_2_B_1_-P1-P5. Genome size was estimated by the formula: Genome size = k-mer_num/Peak_depth where k-mer_num was the total number of k-mers and Peak_depth was the expected value of k-mer depth obtained from the distribution curve. The number of k-mers (generally K = 17) was calculated from short fragment size reads with a one bp slide, and then the frequency of each k-mer was determined. A distribution curve of depth versus frequency was plotted, where the *x*-axis represents the depth and the *y*-axis represents the proportional frequency at that depth divided by the total frequency of all the depths.

### Development and improvement of genome assembly.

For WGS assembly, clean reads were assembled by SOAP*de novo*^53^ (Version 2.04) (parameters: pregraph -s assembly.lib -K 63 -R -d 1 -o pm; contig -g pm –R; map -s assembly.lib -g pm -k 45; scaff -g pm). The k-mer frequency follows a Poisson distribution when read length << genome size^54^. Short insert libraries were assembled into contigs. The reads were mapped back onto the contigs to estimate overlap between contigs. Gapcloser^53^ (Version1.10, parameter: -a pm.scafSeq.fill -b reads.lib -o pm.scafSeq.fillGap -t 24) within the SOAP*de novo* package was used to fill gaps in the scaffold with paired end reads. BAC-by-BAC sequencing of 100,608 BAC clones was conducted to improve the quality of the genome assembly. Each sequenced BAC was assembled separately by SOAP*de novo*. First, sequences shorter than 2,000 bp or having more than 30% unknown bases in BAC clones were discarded. The remaining sequences were then pooled with WGS scaffolds together to extend and collapse redundant sequences.

For improving WGS-based assembly, BAC- sequence data were included in analysis using Rabbit package^55^. This package consists of three modules: Relation Finder, Overlapper and Redundancy Remover. In the first step, 40 bp at the end of each sequence was trimmed as they turn to be of lower quality. Then overlapping between sequences were detected by BLAT^56^ with minimum overlap length set to be 3,000 bp. In second module for extension, overlapping with identity greater than 90% were merged and sequences were extended. To avoid the duplicates in the final assembly, segmental duplications and divergent haplotypes were identified and filtered based on the Poisson-based k-mer model following methods described in Liu *et al*.^52^. To evaluate the assembly of the pearl millet genome, we first calculated the length and N50 distribution for the BAC sequences. The BAC lengths ranged from 80-140k, and their N50s were from 10-40k (Supplementary Fig. 22). Gaps can occur in the fragmented BAC assemblies since the insert size of the pair end reads is 500 bp. PacBio reads were processed using Blasr (processed with PBJelly pipeline) to evaluate the assembled sequence.

### GBS and SNP calling on mapping populations.

GBS libraries were prepared using restriction enzyme *Ape*KI as described by Elshire *et al*.^32^. The MAPPOP1 and MAPPOP2 populations were sequenced at 384-plex (that is, 384 samples per flowcell lane) on an Illumina HiSeq 2000, while the MAPPOP3 population was sequenced at 96-plex (96 samples per flowcell lane). SNPs were called using the TASSEL-GBS pipeline in TASSEL v4.1.32^57^. The TASSEL-GBS pipeline incurs an overhead for each separate pseudomolecule processed, hence we concatenated the thousands of individual scaffolds into ∼20 megascaffolds to ease computation. Reads were processed into clean 64 bp “tags” and mapped against the reference scaffolds with Bowtie 2 (ref. 58). SNPs were called with the DiscoverySNPCallerPlugin in TASSEL, with minimal filters to reduce the number of false positives due to sequencing errors (minor allele frequency ≥ 0.01, minor allele count ≥ 10, genotype calls in at least 10% of samples) (Supplementary Code 1).

### RAD sequencing.

Genomic DNA of each B- and R- individual was digested with EcoRI. After electrophoresis, DNA fragments of the desired lengths were gel purified. Adaptor ligation and DNA cluster preparation were performed and fragments were sequenced on an Illumina HiSeq 2000 platform. Similarly, 29 DNA libraries were constructed for B- and R- lines (580 samples) and sequenced using the RAD-Seq approach^33^.

### Genetic map construction.

SNPs called from the GBS data on three populations (MAPPOP1, MAPPOP2 and MAPPOP3) were first filtered for quality based on minor allele frequency, missingness and heterozygosity (Supplementary Code 2). Linkage groups were defined based on hierarchical clustering of SNPs and ordered with MSTMap. For each population, we created three maps: one from stringently filtered SNPs, one from moderately fileted SNPs, and one mapping GBS sequencing tags back to the stringently filtered map (Supplementary Code 2). The framework map generated in the largest RIL population (Tift 99B × Tift 454) formed the basis of an initial colinearity study between pearl millet and foxtail millet, and the resulting comparative knowledge was used to incorporate additional scaffolds for which orthology to the foxtail millet genome had been established using BLASTP (to identify putative orthologous pearl millet and foxtail millet genes at an E-value threshold of 1e-5) and MCScanX^59^ (to identify colinear segments of at least five syntenic genes between pearl millet and foxtail millet) analyses into the framework map. The genetic maps generated for each of the crosses, and the map that we built based on collinearity information between pearl millet and foxtail millet, were merged using ALLMAPS^60^ with the most weight assigned to the synteny map followed by the stringent SNP maps, the moderately filtered SNP maps, and finally the GBS sequencing tags (Supplementary Code 3). Linkage group numbering was adopted as per an existing consensus map^17^ based on mapping SSR sequences to the assembled genome (Supplementary Code 3).

### Repeat annotation, gene prediction and genome annotation.

We searched the genome for tandem repeats with Tandem Repeats Finder^61^ (Version 4.04) (parameters: 2 7 7 80 10 50 2000 -d -h). Transposable elements (TEs) were identified in the genome by a combination of homology-based and *de novo* approaches^62^. For homology-based predictions, we used the repeat database Repbase16.10^63^ to identify known repeats in the genome assembly with the program RepeatMasker^64^ (Version 3.3.0) (parameter: -nolow -no_is -norna -parallel 1 -lib RepeatMaskerLib.embl.lib). At the protein level, RepeatProteinMask, a software in the RepeatMasker package, was used to perform RMBlast against the TE protein database (parameter: -noLowSimple -pvalue 0.0001). For *de novo* prediction, the programs RepeatModeler^65^ (Version 1.0.5) and LTR_FINDER^66^ (Version 1.0.5) were used on the entire genome to generate a pearl millet repeat database, which was subsequently used as input library with RepeatMasker (Version 3.3.0) to identify TEs.

For predicting genes, we applied several approaches: (i) Homology-based prediction: Proteins previously annotated in other species (Supplementary Table 9) were mapped to the genome using BLAT^56^ (Version 34) with default parameters. Alignments in which the coverage of the query protein was less than 0.3 were removed. In addition, if there were multiple BLAT hits (BLAT output was set to the five best hits), secondary hits were removed if their aligned length was less than 0.3 of the aligned length of the top BLAT hit to filter paralogs with lower sequence identity. GeneWise^67^ (with parameter -trev -sum -genesf) was used to predict spliced alignments. (ii) *De novo* gene prediction: AUGUSTUS^68^ (Version 2.5.5,–species = maize–uniqueGeneId = true–noInFrameStop = true–gff3 = on–strand = both) and Fgenesh^69^ (Version 1.3) were used to detect gene models in the repeat masked genome. (iii) Prediction based on transcript sequences: The assembled transcriptome sequences were aligned to the genome assembly using BLAT (Version 34) using the parameters identity ≥ 0.98 and coverage ≥ 0.98 to generate spliced alignments. (iv) Integration evidence: Source evidence generated from the three approaches mentioned above were integrated using GLEAN^70^ to produce a consensus gene set.

To annotate the function of the final gene models, protein sequences were aligned against KEGG^71^ (release 58) and SwissProt^18^ (release 20156) with BLASTP (E-value ≤ 1.0e-05) to find the best matches. InterProScan^19^ (Version4.8, performed with profilescan, blastprodom, hmmsmart, hmmpanther, hmmpfam, fprintscan and patternScan analysis) was used to identify motifs and domains in the proteins encoded by the gene models along with gene ontology annotations^72^. For ncRNA annotation, tRNA genes in the assembly were identified by tRNAscan-SE^73^ (Version 1.23). rRNA genes were aligned with plant query sequences (rRNA from *Arabidopsis* and rice species) using BLASTN with an E-value threshold of 1.0e-05. Other non-coding RNAs, such as miRNAs and snRNAs were predicted by homology searches against the Rfam database^74^ using the INFERNAL^75^ (Version 0.81) software.

### RNA seq data generation and development of transcriptome assembly.

The transcriptome sequence data were generated from individuals “9-8” and “3-9” accessions at IRD. Library preparation and sequencing (PE 100 bp) on an Illumina Hi-Seq 2000 platform was performed by Fasteris (Plan-les-Ouates, Switzerland). A total of 81,207,232 and 74,187,066 sequence reads were obtained for “3-9” and “9-8”, respectively. Adaptor sequences were trimmed and reads were processed for *de novo* assembly using Velvet 1.0.18^76^ and then Oases 0.1.18^77^. Several values of hash length were tested to optimize the assembly: 39, 51, 63, 65, 69 and 73. The obtained assemblies were compared for their ability to map raw reads using BWA^78^. We consequently decided for a hash length of 73. The transcript assembly was then searched for redundancy. Contigs sharing identity over ≥95% of the length of the shortest sequence in a set of putative homologous sequences were clustered. The final transcript assembly contained 50,313 contigs, with a total of 36,479,993 nucleotides. Three transcriptomes (Zeng *et al*.^16^, Rajaram *et al*.^17^, and the transcriptome data generated at IRD, France, available under BioProject ID PRJNA391885) were combined and clustered using CDHIT-EST^79^ with default parameters to eliminate redundancy at the sequence level. Then, CAP3^80^ was used to assemble the contigs. Ns on either end of the resultant contigs were trimmed. Finally, contigs of at least 200 bp in length were used in gene annotation.

### Gene family and phylogenetic analysis.

For gene family analysis, BLASTP with an E-value cutoff of ≤ 1.0e-05 was used to compare all annotated pearl millet protein sequences against a protein data set of 10 sequenced plant species (*Arabidopsis*^20^, *Brachypodium*^21^, banana^22^, barley^23^, foxtail millet^11^, maize^24^, rice^15^, sorghum^14^, soybean^25^ and *T. urartu*^26^). The proteins were clustered using OrthoMCL^27^ (–mode 3) to define gene families which included both paralogs and orthologs. The number of gene families in each species and genus was calculated based on the composition of the OrthoMCL clusters. Genes that were single copy in an OrthoMCL cluster for all species analyzed were selected to construct a phylogenetic tree using the PhyML (parameters: -d nt -b -4 -m HKY85 -a e -c 4 -t e) program^81^ (Version 3.0). Divergence times between pearl millet and other species were estimated using MCMCTREE^82^ with default parameter. First, the gene family size for each species was calculated based on the output of OrthoMCL, and rooted tree in newick format. CAFE^83^ (-p 0.05 -t 4 -r 10000 -filter) was used to predict the expansion and contraction of gene family numbers based on the phylogenetic tree and gene family statistics.

### Population analysis.

Population genetic analyses of the PMiGAP lines, including PCA and diversity detection were conducted essentially as described for rice by Xu and colleagues^84^. We used a subset of 450,000 SNPs, with a missing rate <10% across PMiGAP lines and wild accessions. Briefly, for PCA, eigenvector decomposition of the SNP genotype data was calculated using the R function eigen^85^. A Tracey-Wisdom test with default parameter settings was performed to determine the significance of axes using the twstats program. To build a phylogenetic tree, the percentage of pairwise nucleotide differences between individuals (p-distance) was calculated^85^. The program fneighbor (PHYLIPNEW v3.69.650 within the package EMBOSS v6.6.0.0; parameter: -matrixtype s -treetype n) was used to construct a neighbor joining tree. The resulting tree was edited and visualized using MEGA5^86^ by choosing Radiation style. Population structure was assessed using the program Snmf (–k K –c)^87^. Five runs were performed and the values with the smallest Cross-Entropy for K from 2 to 7 were selected to generate the structure graphs. To better assess the structure, we performed the analysis in a geographical context, using TESS3^88^ that takes geographical coordinates of the sample into account. Furthermore, parameters of population genetic diversity π, θ_ω_ and differentiation (F_ST_) were calculated based on the SNP data as described earlier^85^. To analyze diversity across the genome, we used a window of 100 kb and calculated the diversity π, θ_ω_ and differentiation F_ST_ for each window for PMiGAP lines and wild accessions using BioPerl modules (Bio::PopGen::Statistics and Bio::PopGen::PopStats) on a sliding window of 100 kb using genotype data. The effective sequence length (without Ns) in each window was used as the denominator to calculate per-bp values. We then calculated a minus log of the ratio of diversity between cultivated and wild samples: –log (π cultivated/ π wild). For this log ratio of diversity and differentiation, we retained the most extreme values using a classical threshold of 95% for a unilateral test and a more stringent threshold of 99.5%. This later stronger stringent threshold was used to identify the most likely gene candidates selected during domestication. Loci with higher levels of differentiation (most extreme F_ST_) and stronger loss of diversity in the cultivated compared to the wild accessions were considered to be provisionally involved in the domestication process.

### Identification of NBS domain, TIR domain, LRR motif and CC motif.

All pearl millet proteins were assessed for the presence of NBS domains (PF00931, NB-ARC) using the Hidden Markov Model based method implemented in hmmsearch (version 3.0)^89^ with an e-value cutoff = 1. To filter false positive hits, all identified NBS containing proteins were screened against the Pfam-A database. NBS domains that overlapped with other domains identified at lower e-values were filtered out. Likewise, the TIR domain (PF01582) was used as query against all pearl millet proteins with hmmsearch and further checked by looking at the overlapping domains. To detect LRR motifs, predicted NBS encoding proteins were searched against 10 LRR families in LRR clan (CL0022) with an e-value cutoff = 1. All regions predicted as LRR motifs and not overlapping with other domains identified with lower e-values were considered real LRR motifs.

### SNP calling, structural variation and linkage disequilibrium (LD) decay.

Sequence reads generated for the B- and R- lines, PMiGAP lines, and parental lines and wild lines were mapped separately to the pearl millet genome assembly using BWA (v0.6) (parameter: aln -n 0.04 -o 1 -e 30 -i 15 -d 10 -l 35 -k 2 -m 2000000 -t 4 -M 3 -O 11 -E 4 -R 30 -q 0 -I; sampe -a 500 -o 100000 -n 3 -N 10 -c 1.0e-05). The BAM files generated by BWA were sorted and provided as input to the GATK software package^90^ (Version 3.1-1). The UnifiedGenotyper module within GATK was used to detect SNP variants. The variants were filtered using VariantFiltration, a module from GATK (parameters: QD < 2.0 || FS > 60.0 || MQ < 40.0 || HaplotypeScore > 13.0; parameters for indel: QD < 2.0 || FS > 200.0), and the number of variants distribution in intergenic/coding regions were calculated. The data used in the downstream analysis were controlled with MAF 0.05 and missing rate 0.5. SNPs with a mean depth > 100 and missing rate > 0.5 were removed. The remaining SNPs were used in further analyses. Variants for wild lines that used in population structure and domestication analysis were detected together with PMiGAP accessions and processed with the same strategy (BAM and VCF files available at http://ceg.icrisat.org/ipmgsc/).

The BAM files from each resequenced accession was analyzed by Breakdancer (version 1.1.2)^91^ with default parameters to detect structural variation namely, deletions, insertions, inversions, and intra-chromosomal translocations. Breakdancer results of accessions that come from a same line (see Supplementary Table 32) were combined to remove redundancy and to calculate the number and length of the rearrangements.

Using SNP data sets from PMiGAP lines, Haploview software^92^ (-maxdistance 250 –minMAF 0.05 -dprime -memory 5096) was used to calculate correlation coefficient (*r*^2^) values for LD. The average (*r*^2^) values between pairwise distances (bp) were calculated and figures were plotted using R.

### Statistical analysis.

*Phenotyping data and GWAS analysis.* For establishing marker trait associations, 288 test cross hybrids were generated by crossing of PMiGAP lines as pollen parents with a common seed parent ICMA 843-22. These hybrids were grouped by maturity (early, medium early, medium and late) and phenotyped for 20 morphological traits under two drought stress conditions (early and late stress) along with controls (or no stress) for two years (2011, 2012). Experiments were conducted in an alpha-lattice designs with two replications in three test environments during Summer 2011 and 2012 (January to May) in the red precision (RP) experimental fields at the ICRISAT, Patancheru, Telengana, India (545 m above mean sea level, 17.53° N latitude and 78.27° E longitude). The early maturity group consisted of lines which had days to 50% flowering (DFF) from 42-52 days; the medium-early maturity group consisted of lines with DFF from 53-57 days; the medium maturity group consisted of entries with DFF from 58-62 days; the late maturity group consisted of lines which recorded more than 62 days for DFF. Early drought stress is a more severe stress imposed by withholding irrigation from about one week before flowering until maturity. Late stress is a less severe drought stress initiated during early grain-filling by withholding irrigation from 50% flowering time till maturity.

The three test environments consisted of early-onset of stress, late-onset stress, and a common, fully-irrigated non-stress treatment. Drought stress was imposed by withholding irrigation from about one week before flowering in early-onset treatment, while drought stress in the late-onset treatment was imposed by withholding irrigation from 50% flowering. Data were recorded for a total of 20 traits namely, grain yield (GYHA), panicle yield (HYHA), panicle harvest index (PHI), time to 75% flowering (TB), plant height (PH), panicle length (EL), panicle diameter (ED), panicle number (HCHA), number of tillers per plant (Till), biomass yield (BM), grain harvest index (HI), thousand grain weight (TGW), grain number per panicle (GNP), grain number per m^2^ (GNM2), agronomic score (AgS), stover dry matter fraction (DMF) and vegetative growth index (GI). PH, EL, and ED were measured on the main stems of five representative plants of each entry in a plot at maturity. At harvest, data were recorded from the harvested area on plant population (PCHA), panicle numbers (HCHA) and fresh stover yield (FSWTHA). Effective tiller number (Till) was calculated as the ratio HCHA/PCHA. HYHA, GYHA and TGW were recorded after oven drying for about 24 h. Stover dry matter yield (DMY) was estimated from plot FSWTHA using the fresh and dry weights of a chopped subsample of stover from each plot. BM was calculated as HYHA + DMY on a plot basis. Grain number per panicle (GNP) was derived from primary data as [(GYHA/HCHA)/ (TGW/1000)]. Grain harvest index was calculated as the ratio between grain yield and biomass yield at harvest, and panicle harvest index as the ratio between grain weight and panicle weight. Flowering time was recorded as days from seedling emergence to stigma emergence for 75% of the main shoots in a plot. The traits measured include grain yield (kg/ha), panicle yield (kg/ha), panicle harvest index (%), time to 50% flowering (number of days), plant height (cm), panicle length (cm), panicle diameter (cm), panicle number, tillers per plant, biomass yield (kg/ha), vegetative growth index (kg/ha/day), grain harvest index (%), fresh stover yield (t/ha), stover dry matter yield (kg/ha), stover dry matter fraction, 1000-grain mass (g), grain number per panicle, and grain number per m^2^ (Supplementary Data set 2). Analysis of variance for all traits was performed using the PROC MIXED procedure in SAS 9.3 (SAS Institute Inc 2013) with Kenward-Roger degree of freedom approximation method considering replicates and accessions as fixed effects, whereas incomplete blocks within each replication were considered as random effects for combined intra and inter block analysis. Best linear unbiased estimates (BLUEs) were calculated for all accessions.

For GWAS analysis, a total of 3,117,056 SNPs retained after filtering the minor alleles (MAF<0.05) and 20% missing data were used. Marker-trait associations were established using AOV model with a bloc effect for maturity group in R (Phenotype∼Bloc+SNP). We tested the suitability of the model by plotting the observed P-values from the association test against an expected (cumulative) probability distribution. These quantile-quantile (q-q) plots clearly indicated that we corrected properly for population stratification (Supplementary Fig. 23). Significance of associations between loci and traits were determined adjusting for multiple testing by using FDR at a 0.001 threshold level and considering p value lower than 10^−10^.

### *Genomic prediction analysis for testcross performance.*

Grain yield performance of 259 PMiGAP lines was used for hybrid prediction analysis. In our analysis, flowering time was considered as a cofactor. For genomic prediction analysis, we performed a one-stage phenotypic data analysis on 259 PMiGAP lines as test cross hybrid trials using a linear mixed model that included genotype, flowering time, year, stress, interaction among genotype, stress and year, replication, incomplete block and residual effects. The effect of flowering time was always assumed to be fixed. When estimating variance components, all other effects were assumed to be random. To get the BLUE of each line, we set the genotype effect as fixed.

The heritability on the line mean basis was estimated as where  and  are variance components arising from genotype, genotype × year interaction, genotype × stress interaction, the three-way interaction and the residual, respectively. *y*, *s* and *r* are the number of different years, stresses, and replications. In addition, we calculated the BLUE for each genotype in each environment (stress versus control) across years. That is, for each environment we fitted a linear mixed model including genotype, flowering time, year, genotype × year interaction, replication, incomplete block and residual effects. The assumptions of the parameters were similar to above. The heritability in this case was estimated as All phenotypic data analyses were done using the ASreml- R 3 software^93^.

A total of 2,235,060 SNPs with <20% missing rates were used with above mentioned phenotyping data for genomic prediction analysis. We used the genomic best linear unbiased prediction (G-BLUP) model for genomic selection: , where *y* refers to *n*-dimensional vector of phenotypic records, 1_*n*_ is an *n*-dimensional vector of ones,  is the mean, *g* is an *n*-dimensional vector of additive genotypic values and *e* is an *n*-dimensional vector of residual terms.

In the model  we assume that  is a fixed parameter, and *g*, *e* are random parameters with  and , where *G* denotes the *n* × *n* genomic relationship matrix. *G* was calculated as follows: Let *X* = (*x*_*ij*_) be the *n* × *p* matrix of SNP markers, where *x*_*ij*_ equals the number of a chosen allele at the *j*^th^ locus for the *i*^th^ genotype. Let *p*_*j*_ be the allele frequency of the *j*^th^ marker. *W* = (*w*_*ij*_) is an *n* × *p* matrix with *w*_*ij*_ = *x*_*ij*_ − 2*p*_*j*_.

Then we have Note that when calculating the kinship coefficient for two genotypes, only those markers without missing values in both genotypes were considered.

The accuracy of genomic prediction was evaluated by fivefold cross-validation with a total of 100 cross-validation runs. The cross-validated prediction accuracy was calculated as the Pearson product-moment correlation between predicted and observed genotypic values of the lines in the test set. The GBLUP model was implemented using the R software^94^.

*Hybrid prediction analysis.*

Grain yield of 64 pearl millet hybrids grown at five locations in India (Jamnagar, Anand, SK Nagar, Mahuva, Kothara) during the time period 2004-2013 was measured. Trials were conducted during 2004, 2005, 2006, 2008, 2011 and 2012 in Kharif, Summer and pre rabi season. However, during 2007, 2009, 2010 and 2013 trials were conducted in only Kharif and Summer. We adopted randomized block design with a spacing of 60 cm between the rows and 10-15 cm between the plants and adopted standard agronomic practices. The 64 hybrids were generated by crossing 20 male and 23 female lines.

By using the grain yield phenotyping data for 64 hybrids as mentioned above, we used the following linear mixed model to estimate the variance components as well as BLUEs:

Yield ∼Genotype + Replication.

To estimate variance components, all effects were treated as random. The BLUEs for each environment were calculated by the same mixed model but modelling genotype as fixed effect. Repeatability was estimated as , where N_R_ refers number of replications,  refers to genetic variance, and  refers to residual variance. Four environments with repeatability lower than 0.5 were removed from further analysis. The BLUEs of the 64 hybrids of each environment were used for an analysis across environments by fitting following model:

Yield∼Genotype + Environment.

The genotype effects were treated as fixed and the environment effects as random. The distribution of the BLUEs across environments approximated a normal distribution. The variance components of genotypes , genotype x environment interactions  and of the residuals  were estimated using a one-step model. Broad-sense heritability was then calculated as the ratio of genotypic to phenotypic variance:



where *l* refers to the number of environments and r is the average number of replications per environment. The hybrid prediction was based on 302,110 high-quality SNP markers obtained from 580 B- and R- lines. We used ridge regression-BLUP considering additive and dominance effects to predict the hybrid performance. Details of the implementation of the models have been described earlier^95^. Briefly, the general form of the model is defined as the following:

where *1*_*n*_ is a vector of ones and *n* is the number of hybrids, μ refers to the overall mean across all four locations. *Z*_*A*_ and *Z*_*D*_ are *n* × *m* design matrices for the additive and dominance effects of the markers, where *m* refers to the number of markers. The elements of *Z*_*A*_ are -1, 0, 1, and elements of *Z*_*D*_ is 0, 1. While *a* = (*a*_1_, *a*_2_, ..., *a*_*m*_)^*T*^ and *d* = (*d*_1_, *d*_2_, ..., *d*_*m*_)^*T*^ are the vectors of length m, and *a*_*i*_ *d*_*i*_ denote the additive and dominance effects for the *i*^th^ marker, respectively. *e* = (*e*_1_, *e*_2_, ..., *e*_*n*_)^*T*^ is a vector of length *n* and *e*_*j*_ is the residual for the j^th^ hybrid.

Prediction accuracy was studied using cross validations. In each cross validation, 48 hybrids were randomly selected as training set and the remaining 16 hybrids were used as test set. The cross validation was run 500 times and accuracy was estimated as the Pearson correlation coefficient between predicted and observed values standardized with the square root of the heritability (h = 0.76). Next, we used all 64 hybrids as a training set and predicted the hybrid performance of 167,910 possible single-cross combinations among the 580 inbred lines (260 B-lines and 320 R-lines). Based on the predicted values, we selected 0.1% hybrids that had the highest predicted yields (170/167,910 hybrids). Of those 170 hybrids, 11 have been bred so far and are thus a subset of the 64 phenotyped hybrids. The remaining 159 hybrids are based on parental inbred lines that have never been used for hybrid breeding and could be tested in the field. All analyses were done using the ASreml-R 3 software^93^.

### Data availability.

Genome sequence assembly and annotation data: BioProject ID PRJNA294988; BioSample ID SAMN04124419. Resequencing data: SRA SRP063925. Transcriptome data: BioProject ID PRJNA391885. BAM and SNP files are available at http://ceg.icrisat.org/ipmgsc. GigaScience Database record: 10.5524/100192 Scripts used in the MS are available at https://github.com/ICRISAT-CEG/PM-Scripts.git

A Life Sciences Reporting Summary is available.

## Additional information

**Publisher's note:** Springer Nature remains neutral with regard to jurisdictional claims in published maps and institutional affiliations.

## Supplementary information

Supplementary Text and Figures Supplementary Figures 1–23. (PDF 4617 kb)

Life Sciences Reporting Summary Life Sciences Reporting Summary. (PDF 114 kb)

Supplementary Tables Supplementary Tables 1–14, 16–18, 22, 24, 28, 32–33, 42. (PDF 1754 kb)

Supplementary Table 15 Summary of genes expanded during pearl millet evolution. (XLSX 12 kb)

Supplementary Table 19 Summary of data generated on the PMiGAP lines using whole genome sequencing. (XLSX 30 kb)

Supplementary Table 20 Data generated on 38 inbred parents of different mapping populations using whole genome resequencing. (XLSX 11 kb)

Supplementary Table 21 Data generated for B- and R-lines of pearl millet using RAD-Seq approach. (XLSX 41 kb)

Supplementary Table 23 Summary of SSR motifs identified, primers designed and their genome coordinates. (XLSX 8049 kb)

Supplementary Table 25 Distribution of SNPs in intra-genic and inter-genic regions across PMiGAP lines. (XLSX 46 kb)

Supplementary Table 26 Distribution of SNPs in intra-genic and inter-genic regions across parental lines of mapping populations. (XLSX 15 kb)

Supplementary Table 27 Distribution of SNPs in intra-genic and inter-genic regions across B- and R- lines. (XLSX 69 kb)

Supplementary Table 29 Insertions and deletions identified in the PMiGAP lines. (XLSX 10 kb)

Supplementary Table 30 Insertions and deletions identified in the parental lines of mapping populations. (XLSX 10 kb)

Supplementary Table 31 Insertions and deletions identified in B- and R- lines. (XLSX 10 kb)

Supplementary Table 34 Regions with loss of diversity and strong differentiation between wild and cultivated pearl millet. (XLSX 101 kb)

Supplementary Table 35 List of the genes found in the regions showing strong differentiation between wild and cultivated germplasm and diversity loss in cultigen. (XLSX 15 kb)

Supplementary Table 36 Genome-wide marker-trait associations for grain and stover yield. (XLSX 109 kb)

Supplementary Table 37 Best 170 predicted hybrid combinations. (XLSX 15 kb)

Supplementary Table 38 Best 11 tested hybrid combinations. (XLSX 10 kb)

Supplementary Table 39 Pedigree details of B- and R- used in the study. (XLSX 31 kb)

Supplementary Table 40 Details of 345 Pearl Millet Inbred Germplasm Association Panel (PMiGAP) lines used in the study. (XLSX 35 kb)

Supplementary Table 41 Details of 38 parental lines of mapping populations of pearl millet used in the study. (XLSX 12 kb)

Supplementary Code 1 (ZIP 1 kb)

Supplementary Code 2 (ZIP 56 kb)

Supplementary Code 3 (ZIP 26 kb)

Supplementary Dataset 1 (XLSX 132 kb)

Supplementary Dataset 2 (XLSX 347 kb)

## Data Availability

BioProject
PRJNA294988
PRJNA391885 Sequence Read Archive
SRP063925
