# Supplementary material for: Pearl millet genome sequence provides a resource to improve agronomic traits in arid environments
Source: Nat Biotechnol. 2017 Sep 18;35(10):969–76. doi: 10.1038/nbt.3943 (PMC6871012; doi:10.1038/nbt.3943)
Supplement: Supplementary Text and Figures — Supplementary Figures 1–23. (PDF 4617 kb) [file 41587_2017_BFnbt3943_MOESM47_ESM.pdf]

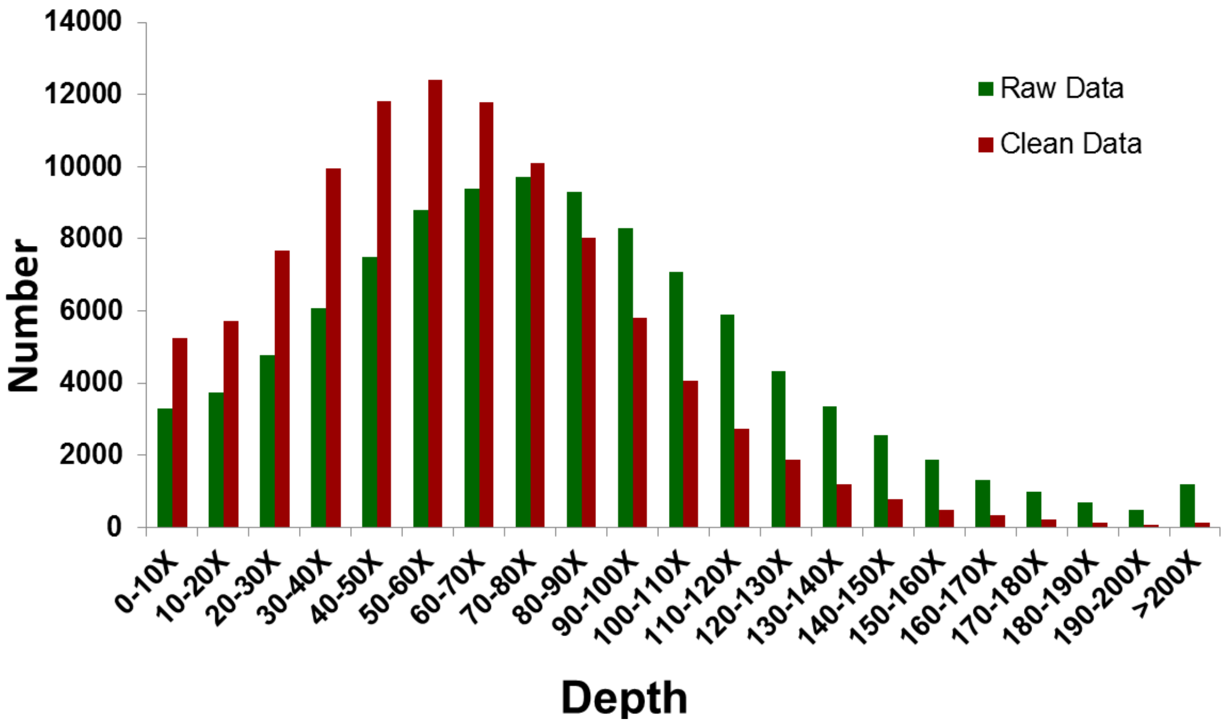

Supplementary Figure 1. Distribution of sequence depth across the bacterial artificial chromosomes (BACs). The x-axis denotes the sequencing depth (X) of each BAC and y-axis denotes the number of BACs corresponding to depth.

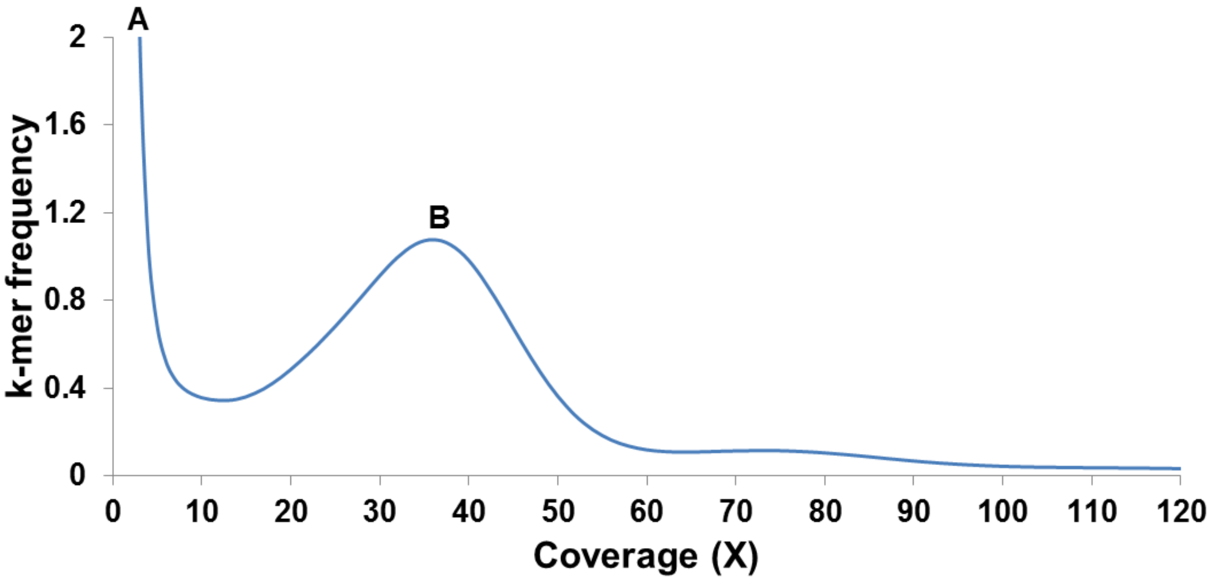

Supplementary Figure 2. Estimation of the pearl millet genome size based on K-mer statistics.

The frequency distribution of 17-mers within the raw genomic reads displays 2 major peaks (A and B). Peak A resembles a Gaussian distribution and represents k-mers of ~0-10X coverage which arise by chance due to sequencing errors. Peak B, corresponding to k-mers of ~20-50X coverage, represents the majority of the genome and resembles a Poisson distribution with minor differences due to sequencing errors, heterozygosity and repetitive DNA. The total genome size of pearl millet was estimated by obtaining the multiplication product of 17 bp and the k-mer frequency (value at y-axis) corresponding to the coverage (value at x-axis) at Peak B (*i.e.* 17X Peak B frequency).

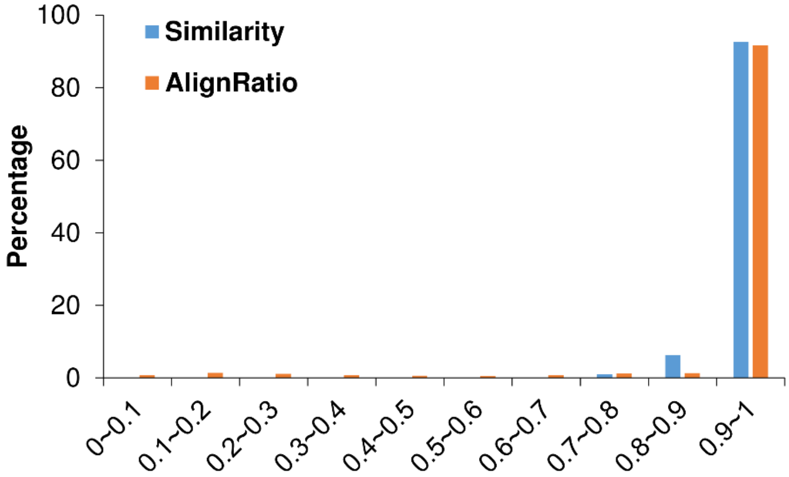

Supplementary Figure 3. Percent similarity and align rate for long PacBio reads vs scaffold sequences.

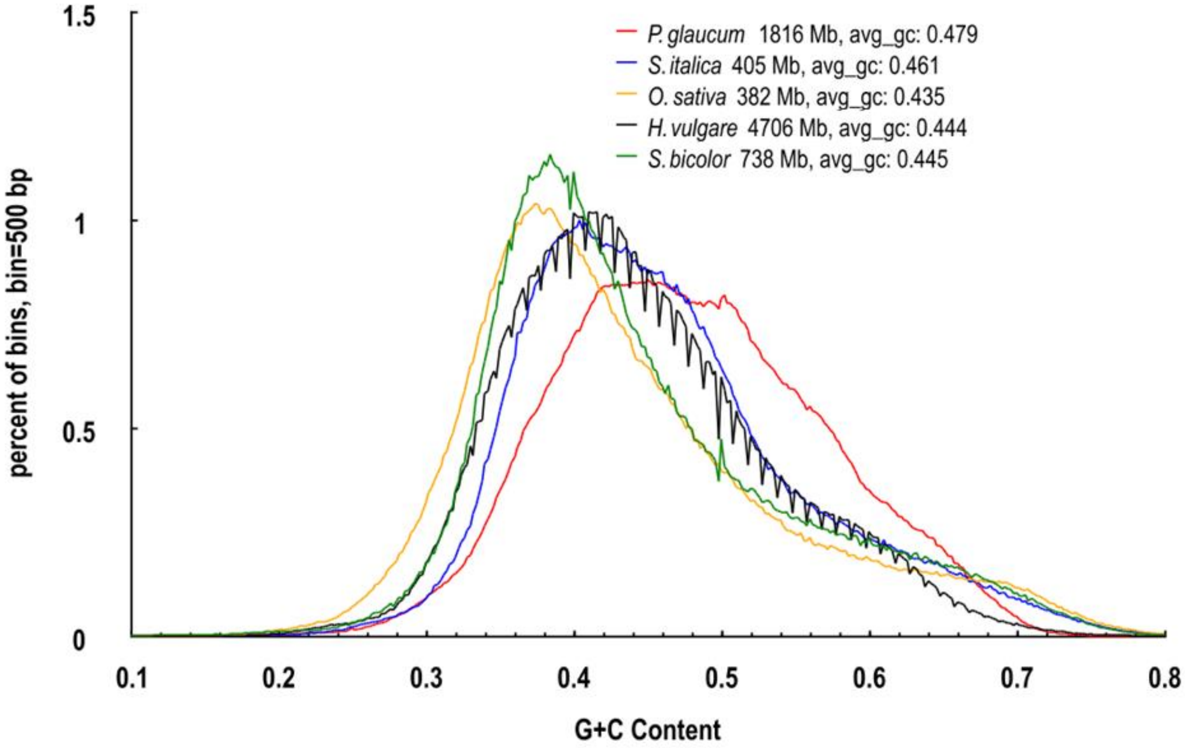

Supplementary Figure 4. GC content distributions in selected grass genomes. The mean GC content of pearl millet is higher than barley (*Hordeum vulgare*), foxtail millet (*Setaria italica*), rice (*Oryza sativa*), and sorghum (*Sorghum bicolor*). GC content is represented on the x-axis and the proportion of the bin number divided by the total windows on the y-axis.

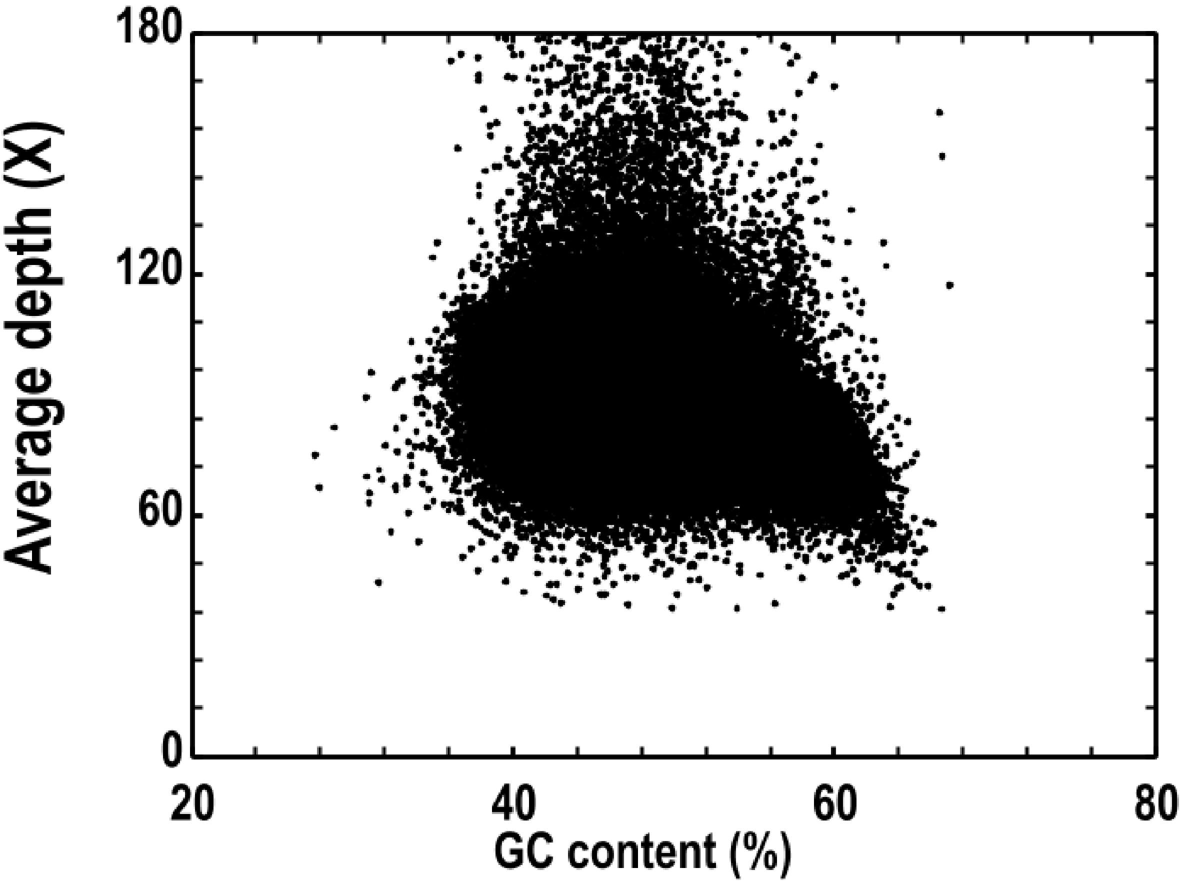

Supplementary Figure 5. Scatterplot showing GC content *versus* sequencing depth. The graph indicates that no GC bias is present in the sequence data generated.

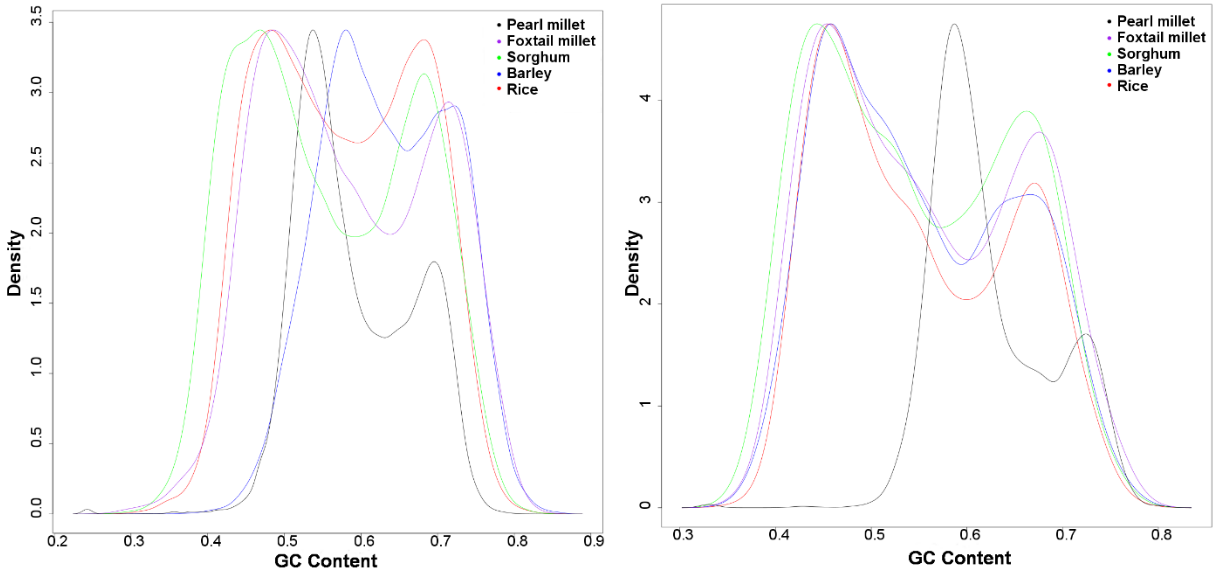

Supplementary Figure 6. GC content distribution of whole genome CDS (left) and 384 pearl millet expanded families (right) in grasses. The GC content in whole genome CDS and 384 expanded gene families were found similar.

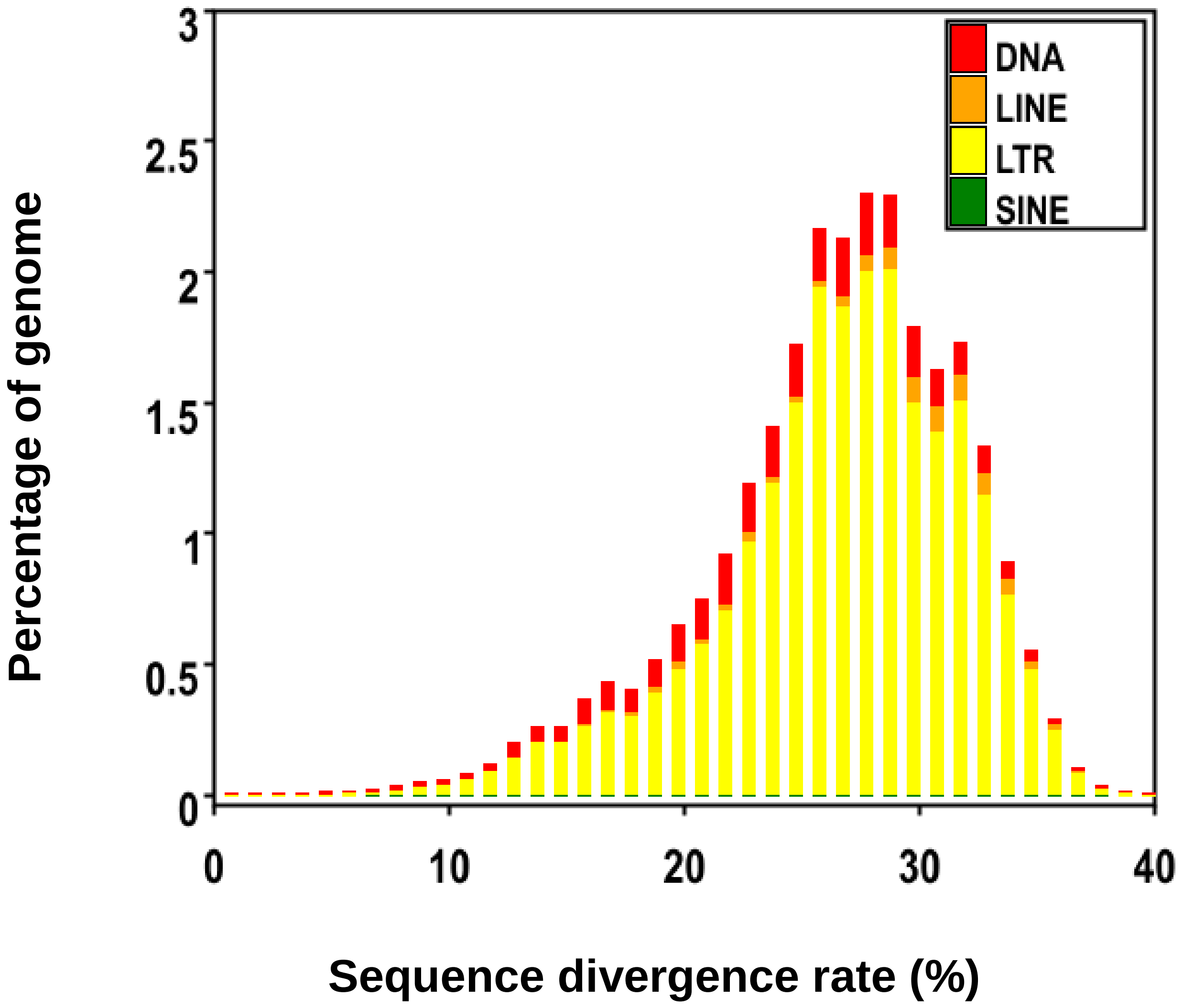

Supplementary Figure 7. Distribution of divergence rates of different transposable element (TE) types in the pearl millet genome. DNA- DNA elements; LINE- long interspersed nuclear elements; LTR- long terminal repeat transposable elements; SINE- short interspersed nuclear elements. Divergence rates were high among LTR elements.

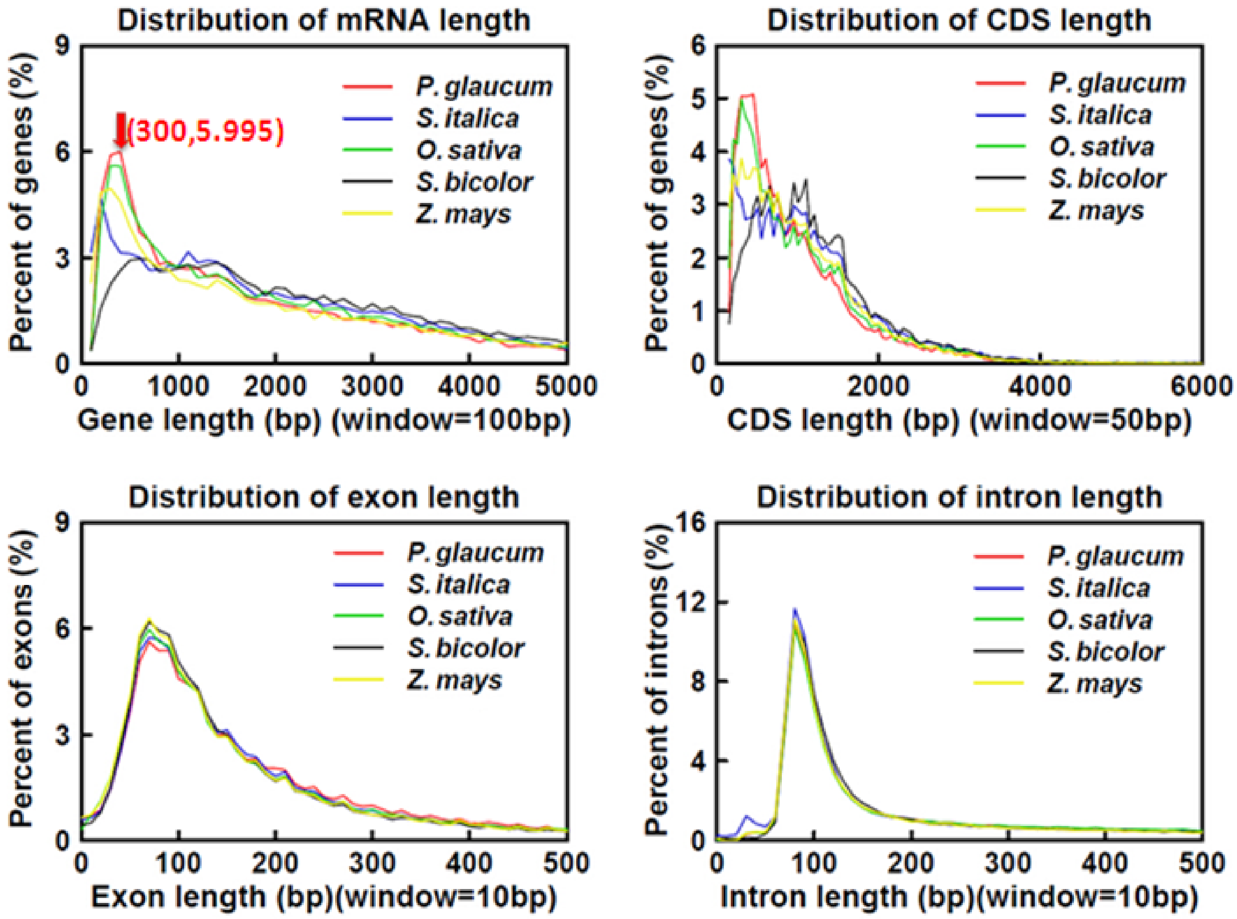

Distribution of mRNA length
Percent of genes (%)
Gene length (bp) (window=100bp)
(300,5.995)
P. glaucum
S. italica
O. sativa
S. bicolor
Z. mays
Distribution of CDS length
Percent of genes (%)
CDS length (bp) (window=50bp)
Distribution of exon length
Percent of exons (%)
Exon length (bp)(window=10bp)
Distribution of intron length
Percent of introns (%)
Intron length (bp)(window=10bp)

a.

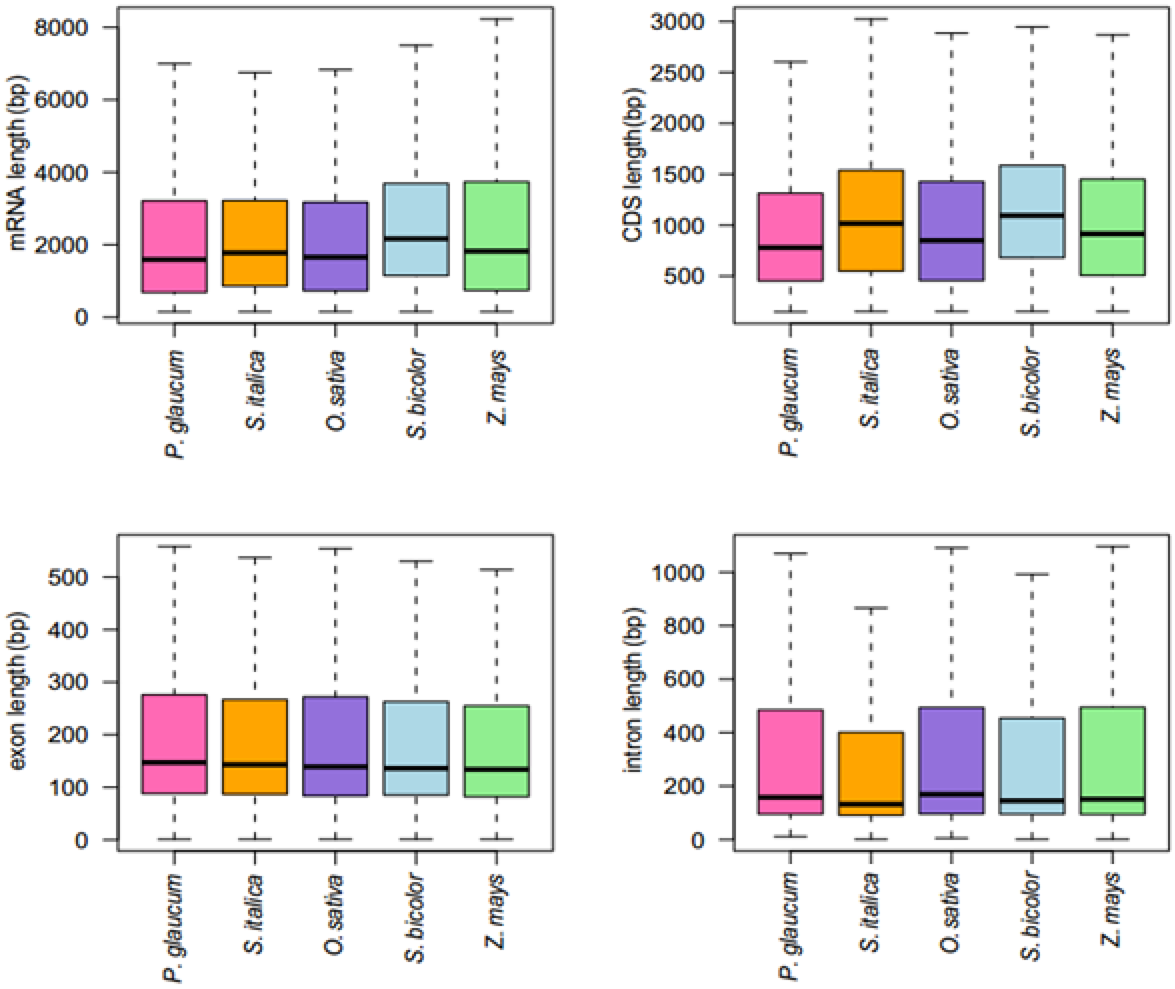

b.

Supplementary Figure 8. Length distribution of mRNA, CDS, exons and introns in sequenced cereal genomes. (a) The x-axis indicates the length, and the y-axis indicates the gene percentage in the corresponding length window. For example, in the figure "Distribution of mRNA length", the coordinate indicated by the red arrow is (300, 5.995), meaning that 5.995% of all gene models have lengths in the range 300-400 bp. **(b)** Boxplots indicate that the average lengths of different gene features in pearl millet are similar to those in the other four cereal species

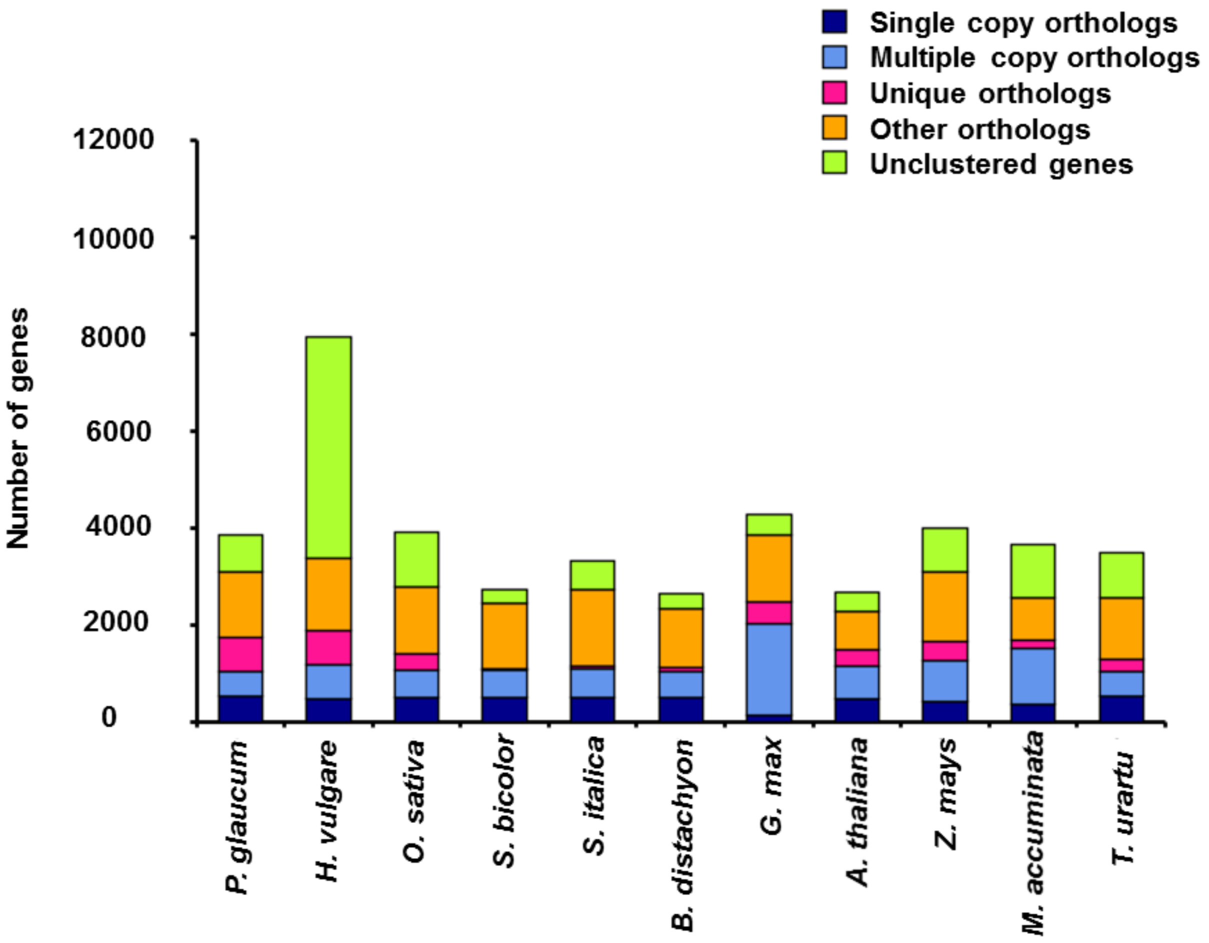

Supplementary Figure 9. An overview of orthologous and paralogous genes in pearl millet. The number of orthologs and paralogs in the pearl millet genome is shown in comparison to those gene classes in the genomes of ten select plant species [Arabidopsis (*Arabidopsis thaliana*), Brachypodium (*Brachypodium distachyon*), banana (*Musa acuminata*), barley, foxtail millet, maize, rice, sorghum, soybean (*Glycine max*) and bread wheat]. Reciprocal pair-wise comparisons of the 38,579 pearl millet gene models with 385,891 gene models from ten select plant species identified 17,949 orthologous groups (Supplementary Table 12), among which 5,232 contained only a single pearl millet gene, suggestive of simple orthology (Supplementary Table 13).

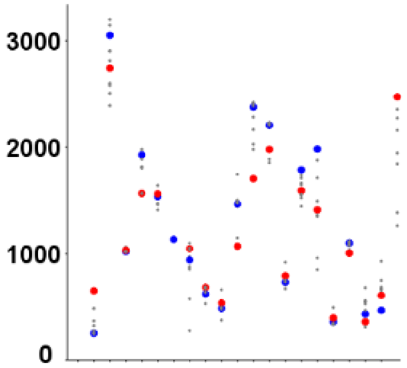

a.

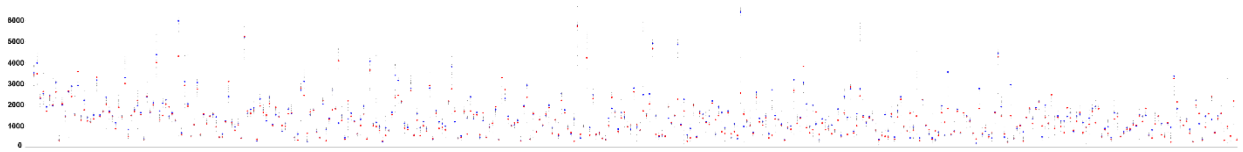

b.

Supplementary Figure 10. Length distribution for each expanded family. (a) 20 expanded gene families randomly plotted. (b) 384 expanded gene families. Red dots represent pearl millet, and blue represent rice.

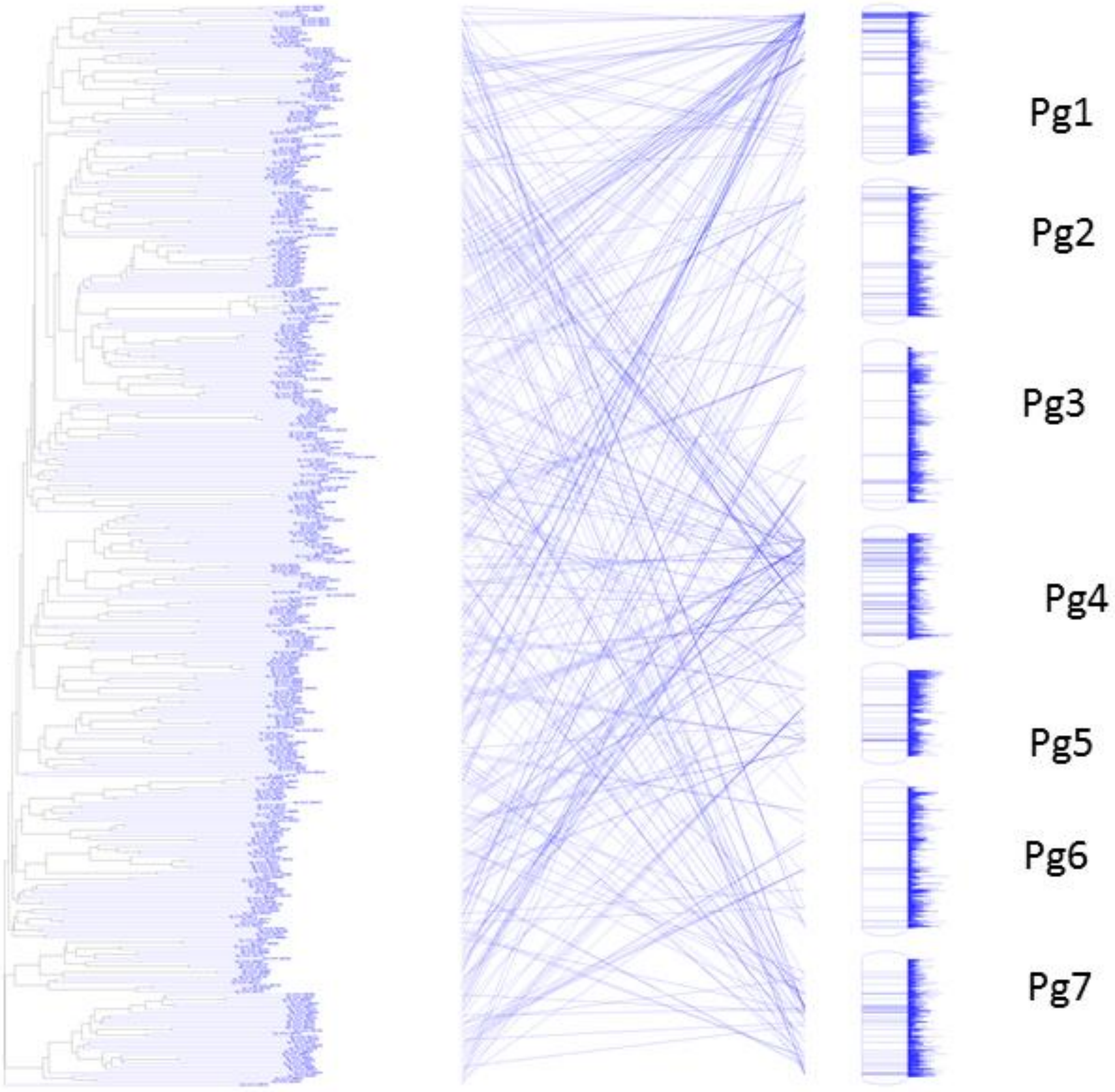

Supplementary Figure 11. Phylogeny of NBS-LRR genes. Heavily tandemized gene groups can be observed at one telomere of Pg1 and Pg4 followed by Pg7.

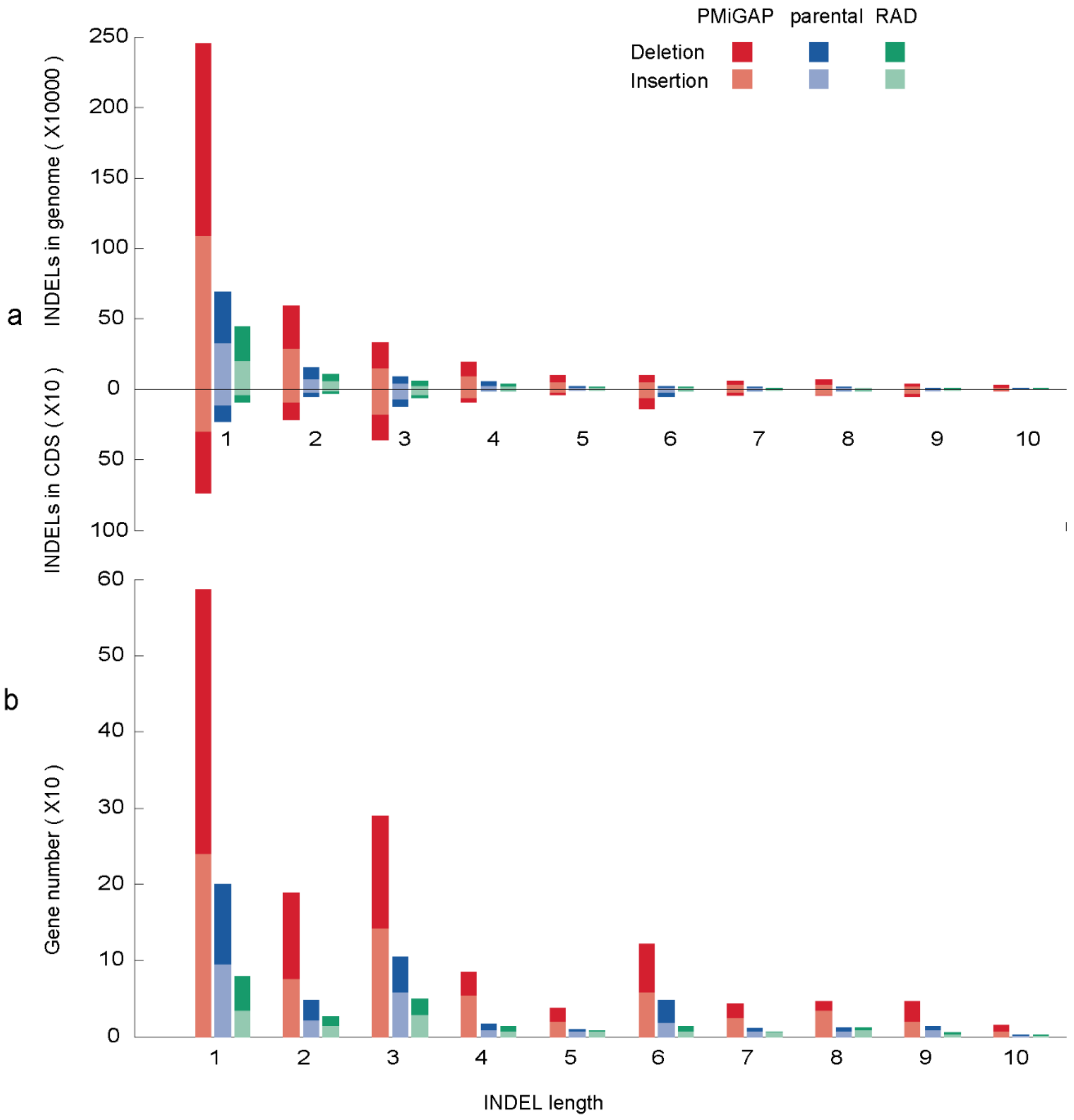

Supplementary Figure 12. (a) Indels and (b) gene numbers in PMiGAP lines, parental lines of mapping population and B- and R- lines. Large number of indels were observed in the genome compared to CDS regions in PMiGAP lines.

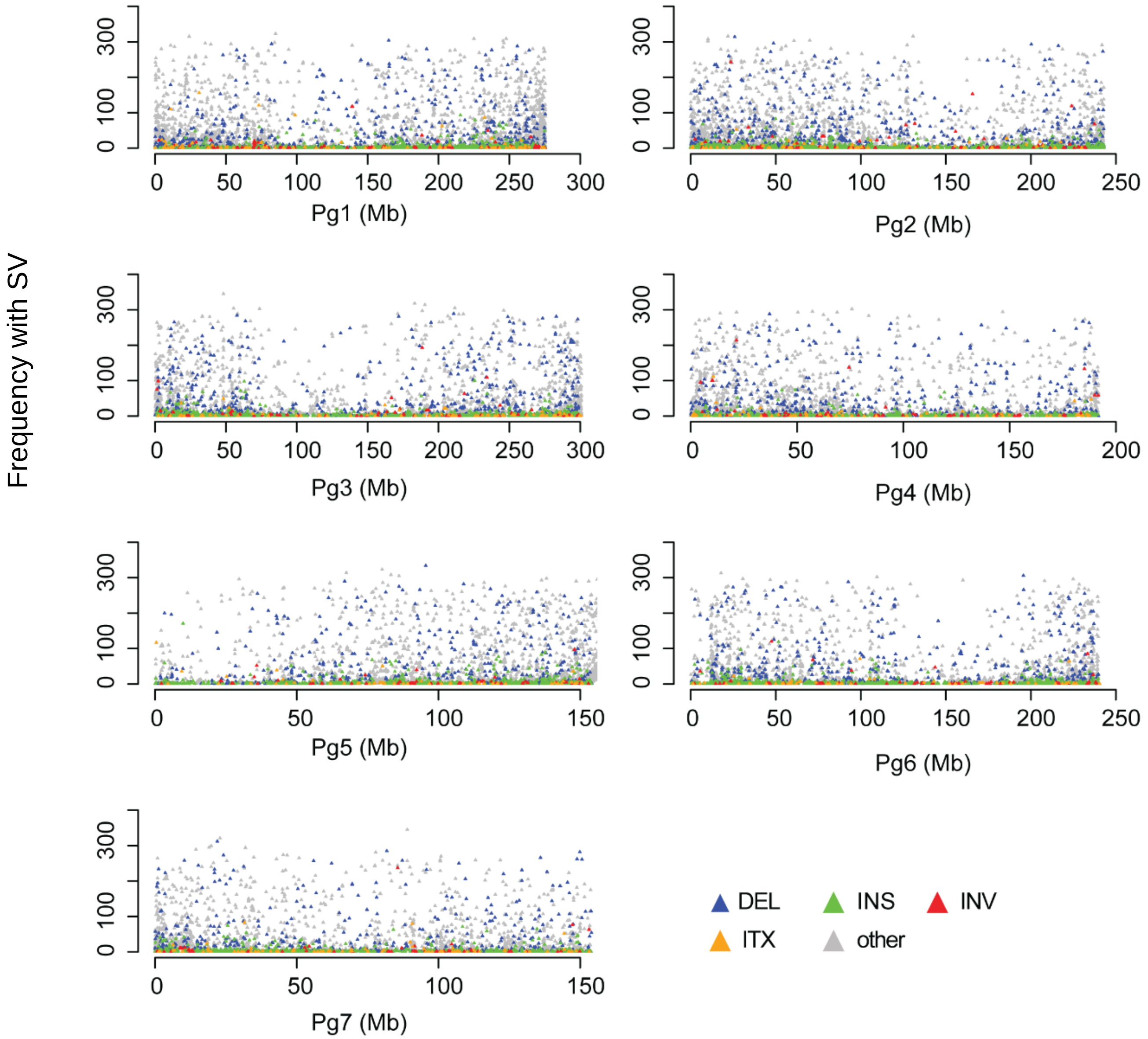

Supplementary Figure 13. Distribution of structural variations in PMiGAP lines. Structural variations such as deletions (DEL), insertions (INS), inversions (INV), intra-chromosomal translocations (ITX) and others were determined using BreakDancer software. Among different structural variations DEL were found in large number across the genome in the PMiGAP lines.

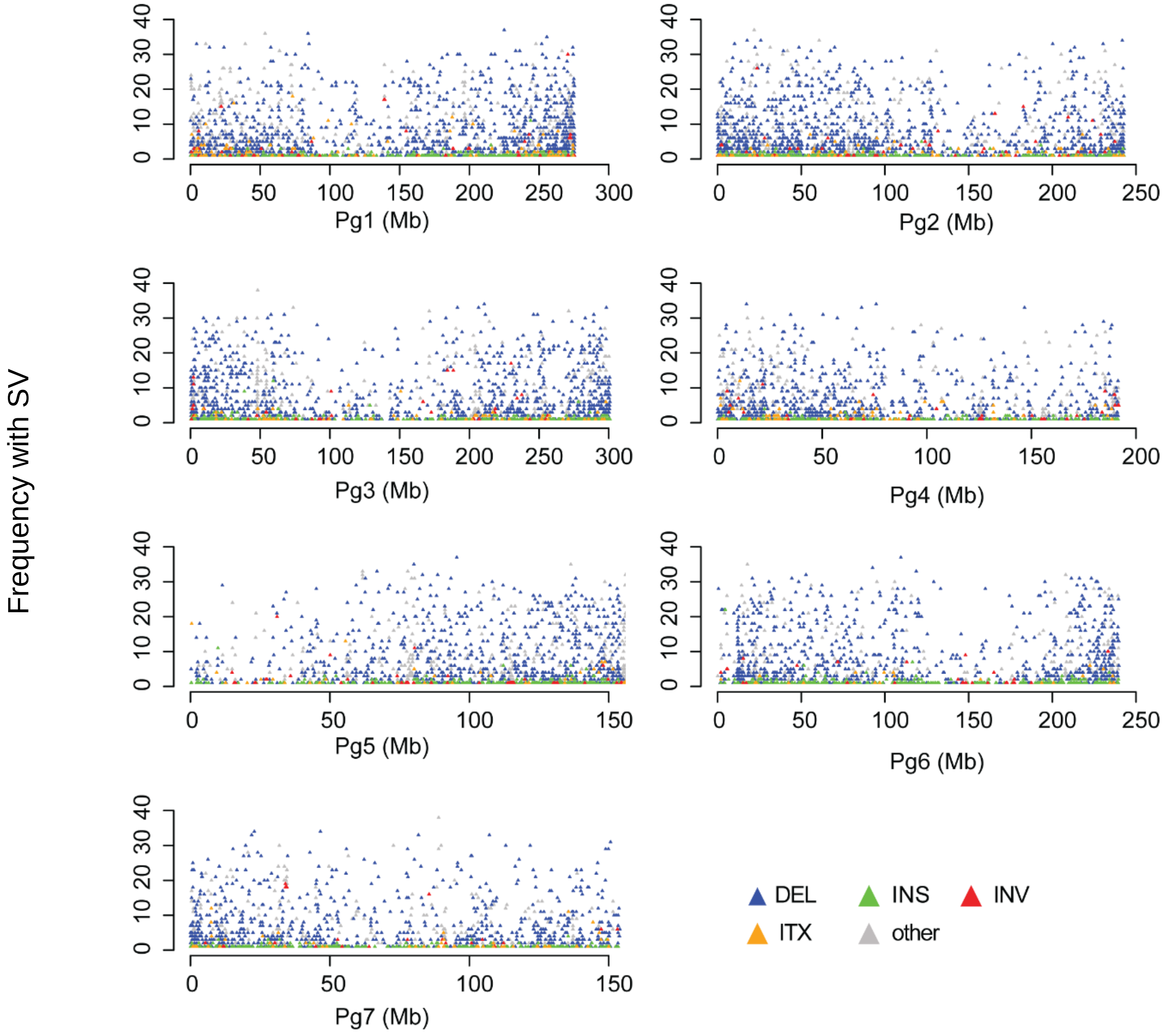

Supplementary Figure 14. Distribution of structural variations across 38 parents of mapping populations. Deletions were found in large numbers across the genome in the parental lines.

Nature Biotechnology: doi:10.1038/nbt.3943

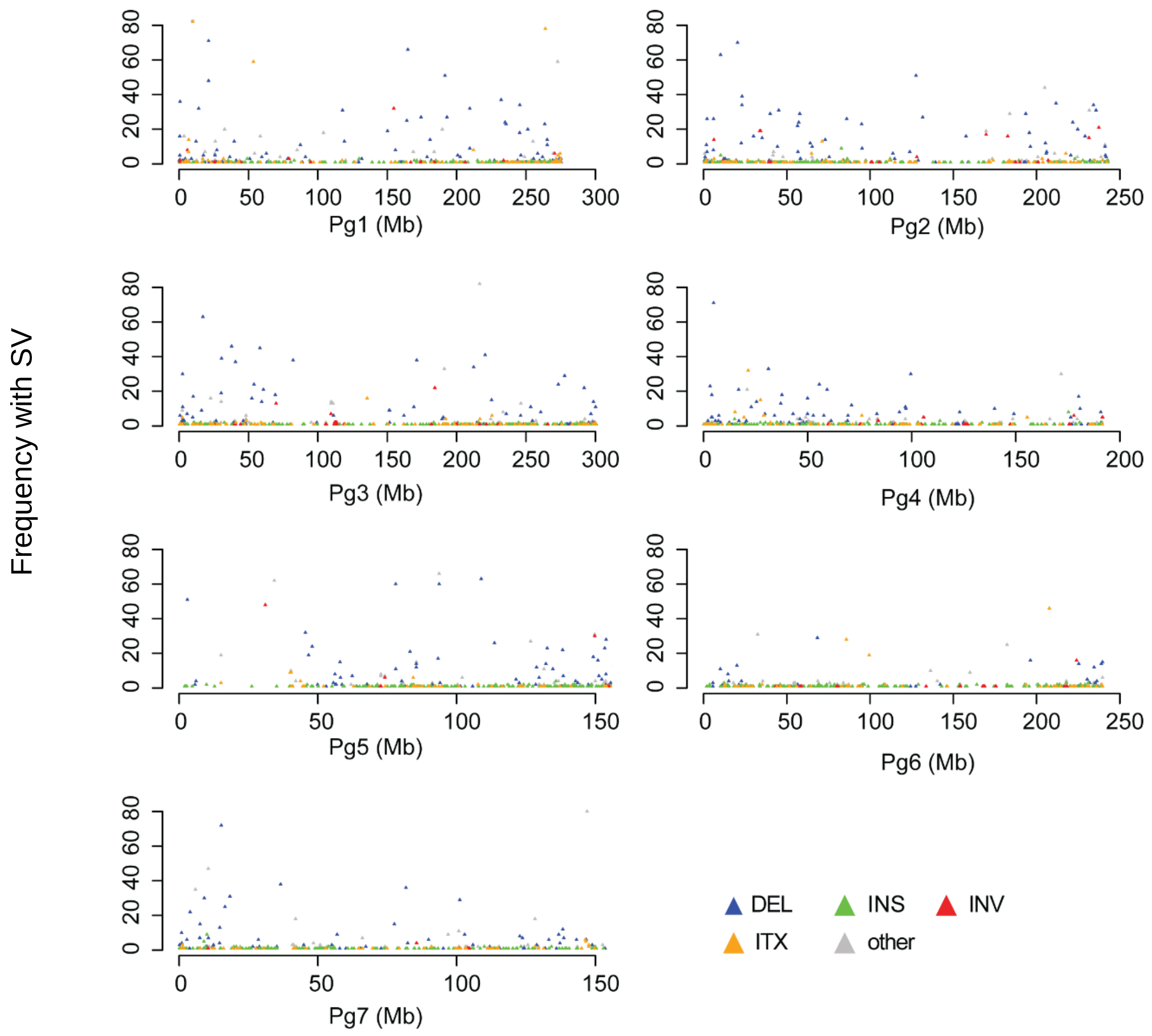

Supplementary Figure 15. Distribution of structural variations in B and R lines. The number of structural variations identified in the B- and R- lines is significantly lower than that identified in case of PMiGAP lines and parental lines of mapping populations. This can be attributed to the RAD-seq approach adopted for sequencing these lines.

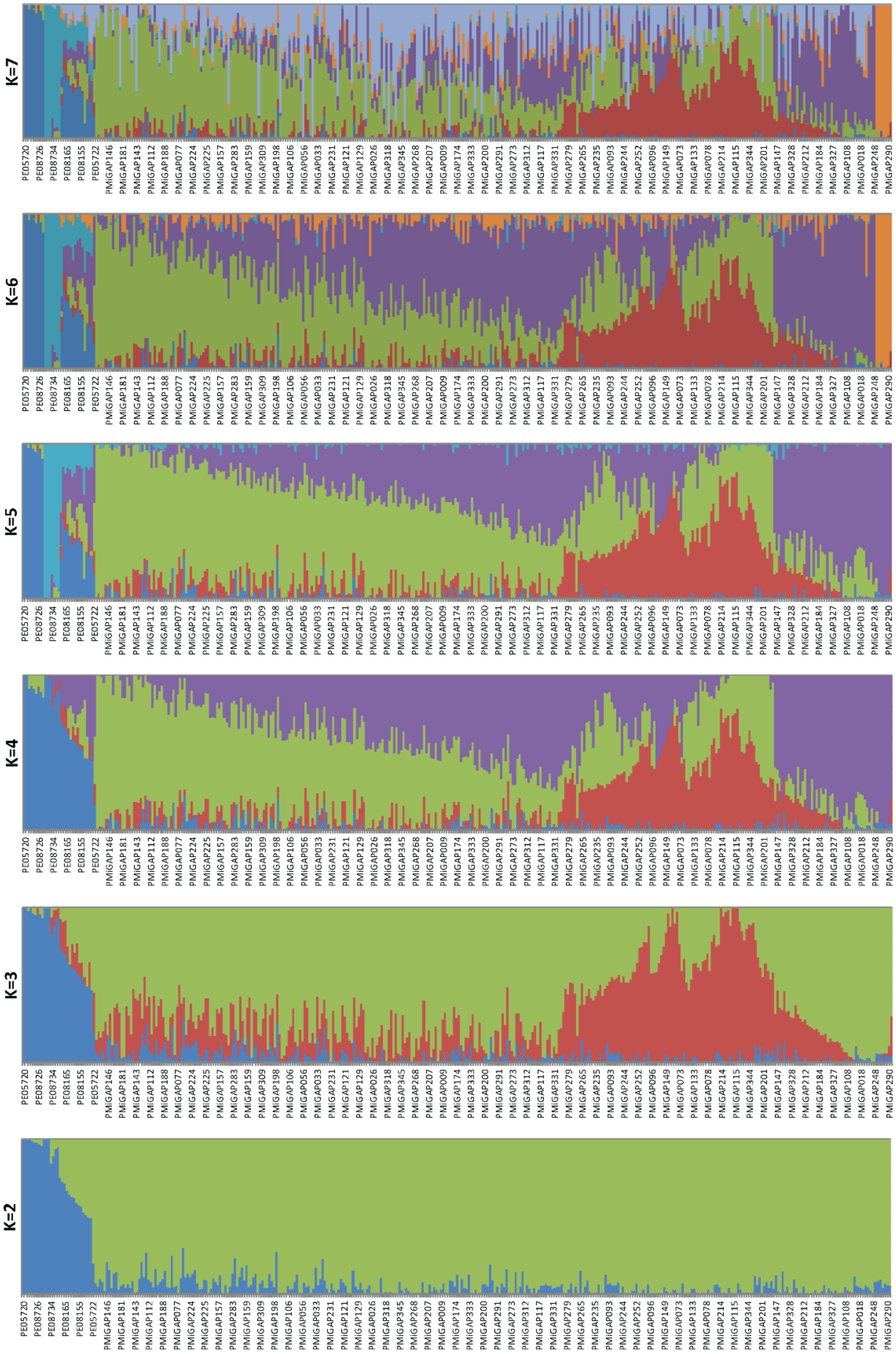

Supplementary Figure 16. Population structure of 345 PMiGAP lines and 31 wild samples. A set of 29.54 million SNPs were used to determine the number of sub-populations in PMiGAP lines using STRUCTURE analysis. Population structure, when K=2, mainly separate all the

cultivated from the wild relatives. At K=3, cultivated are separated but the separation does not correspond to clear geographical pattern, a large fraction of the cultivated share a blue and red component. At K=4 a further division inside the cultivated show ancestry almost admixed for all cultivated. At K=5, wild populations are split in two well defined clusters and a cluster presenting strong similarity to the cultivated. This last cluster is the closest to the cultivated in the PCA, and correspond to the central area of the wild populations, Niger and Mali. The two other well defined cluster correspond to wild from East Africa and Senegal/Mauritania/West Mali. A new cultivated cluster among widely across cultivated appear at K=6 and 7. Structure can be influenced by number of samples as well as imbalance between cultivated and wild populations. Among the East group, two individuals show a closer proximity to the cultivated in the PCA analysis (PE08743 from Soudan and PE08721 from Chad). These two individuals show admixed ancestry 48% cultivated and 24% cultivated respectively. These two sample were sampled at latitude 12.78 and 12.53 respectively. At this latitude wild populations are in sympatry with cultivated varieties and hybridization could occur.

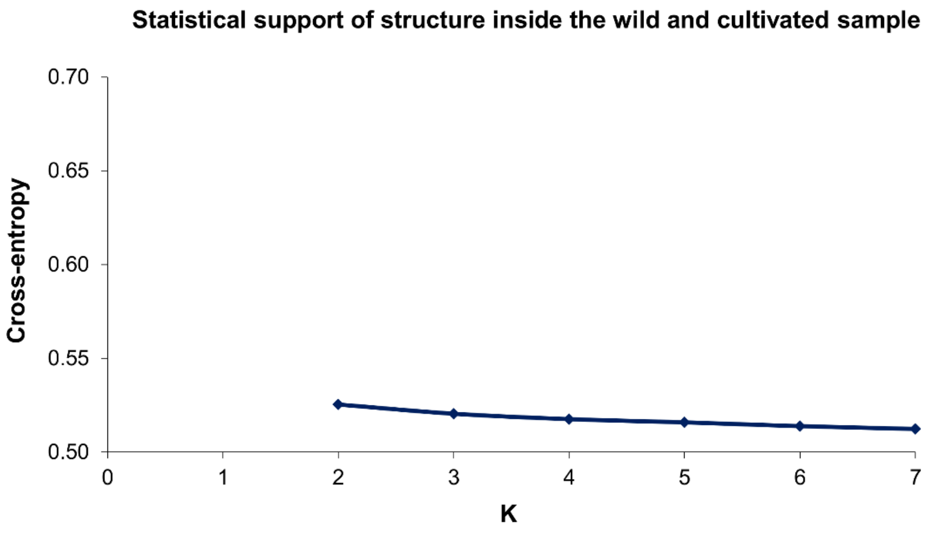

Statistical support of structure inside the wild and cultivated sample
Cross-entropy
0.70
0.65
0.60
0.55
0.50
0
1
2
3
4
5
6
7
K

a.

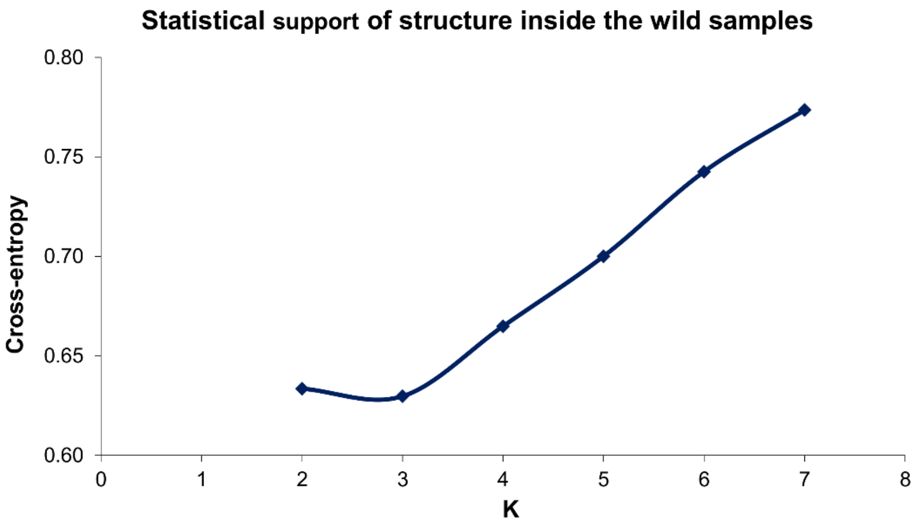

Statistical support of structure inside the wild samples
Cross-entropy
0.80
0.75
0.70
0.65
0.60
0
1
2
3
4
5
6
7
8
K

b.

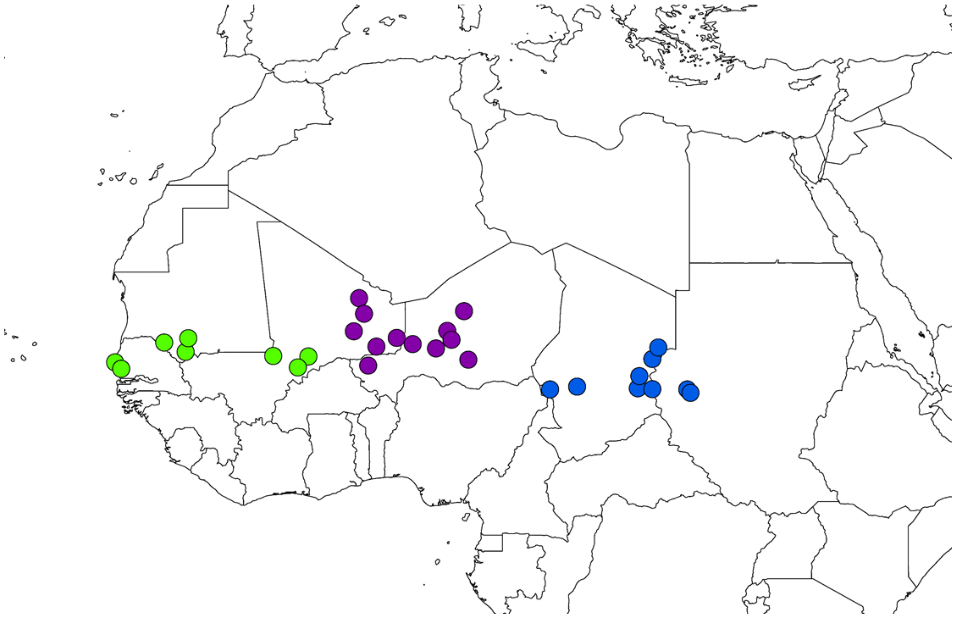

c.

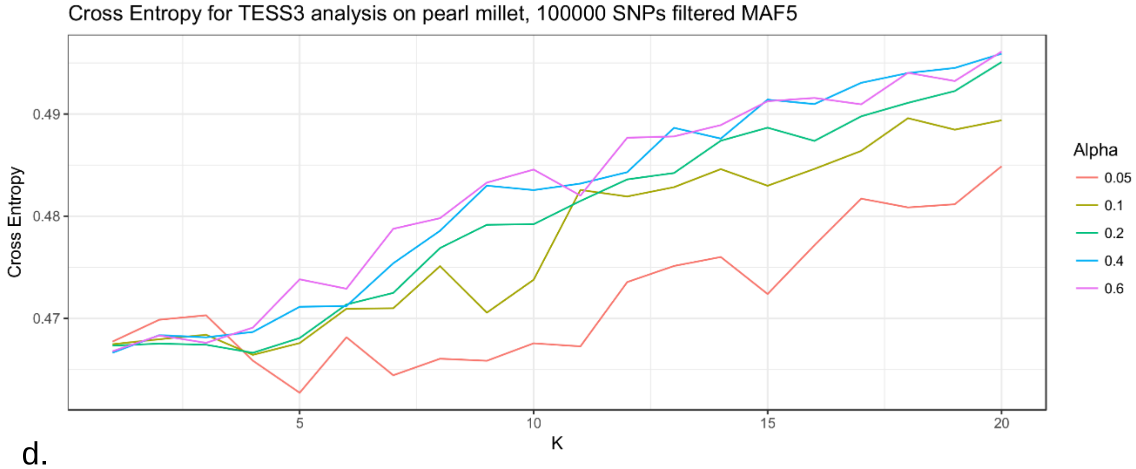

d.

Supplementary Figure 17. **Cross entropy analysis of population structure**. (a) For all wild and cultivated samples, Cross entropy decreased slowly from K=2 to K=7. But, when we considered wild samples alone, (a) We found a strong signal of structure inside the wild populations separating wild individuals from the East, Center and West of the Sahel. The different genetic group are represented (c). The wild pearl millet accessions sequenced in this study clustered in to three main groups West African (green), Central Africa (violet) and East African (blue). Note that we used a similar scale here for these two graphics. To better assess if there is any geographical structuration inside the cultivated (d), we performed a geographically explicit model using TESS3. We made the alpha parameter varied from 5% to 60% allowing to weight the geographical position of the samples in the structure inference. The cross entropy do not vary much and is flat or slightly increase from K=1 (no structure) to K=3. Altogether, these analysis suggest a marked signal of structure inside the wild populations, but not inside the cultivated.

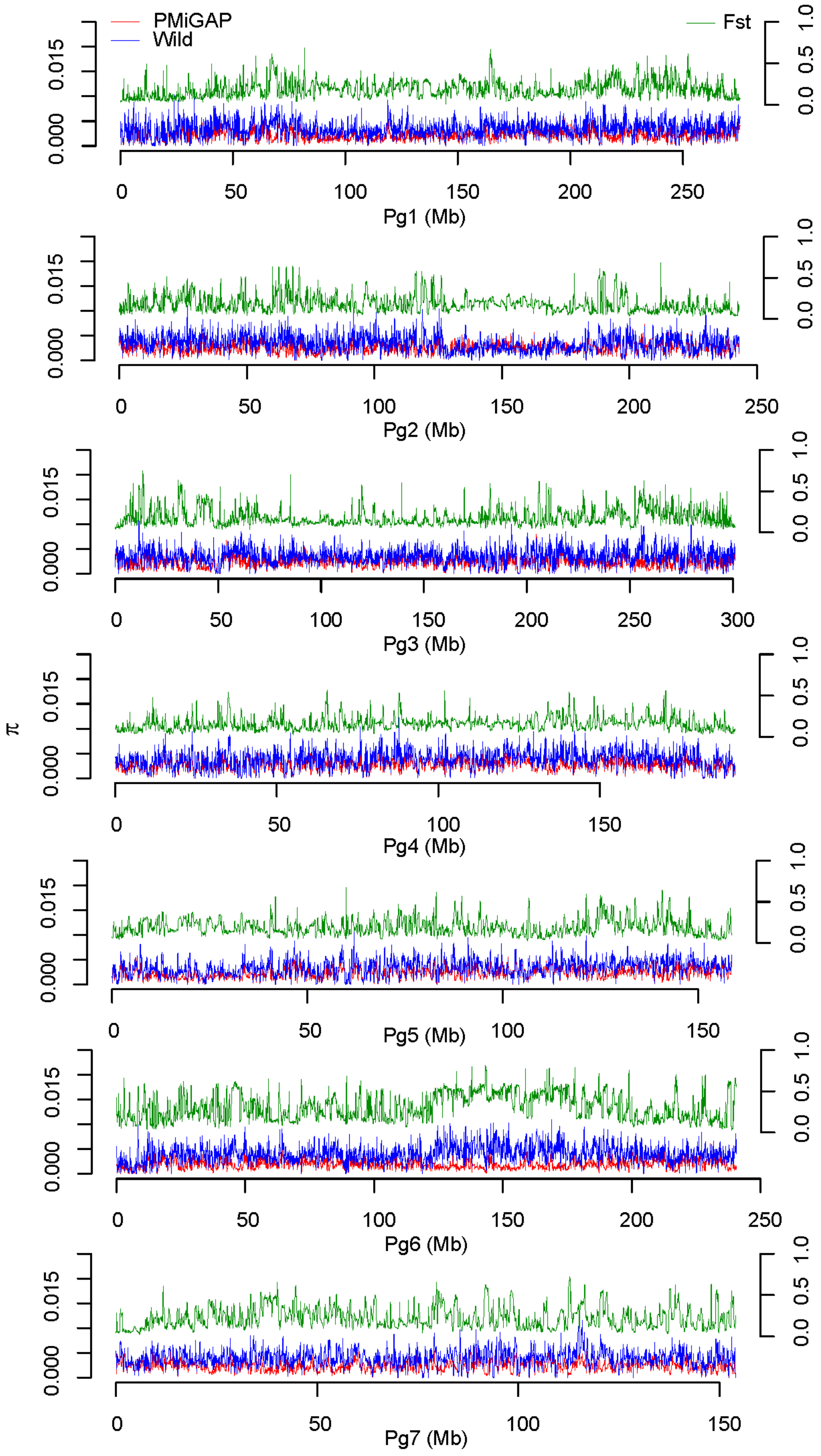
PMiGAP
Wild
Fst
π
0.000
0.015
0.0 0.5 1.0
Pg1 (Mb)
Pg2 (Mb)
Pg3 (Mb)
Pg4 (Mb)
Pg5 (Mb)
Pg6 (Mb)
Pg7 (Mb)
0
50
100
150
200
250
300

a.

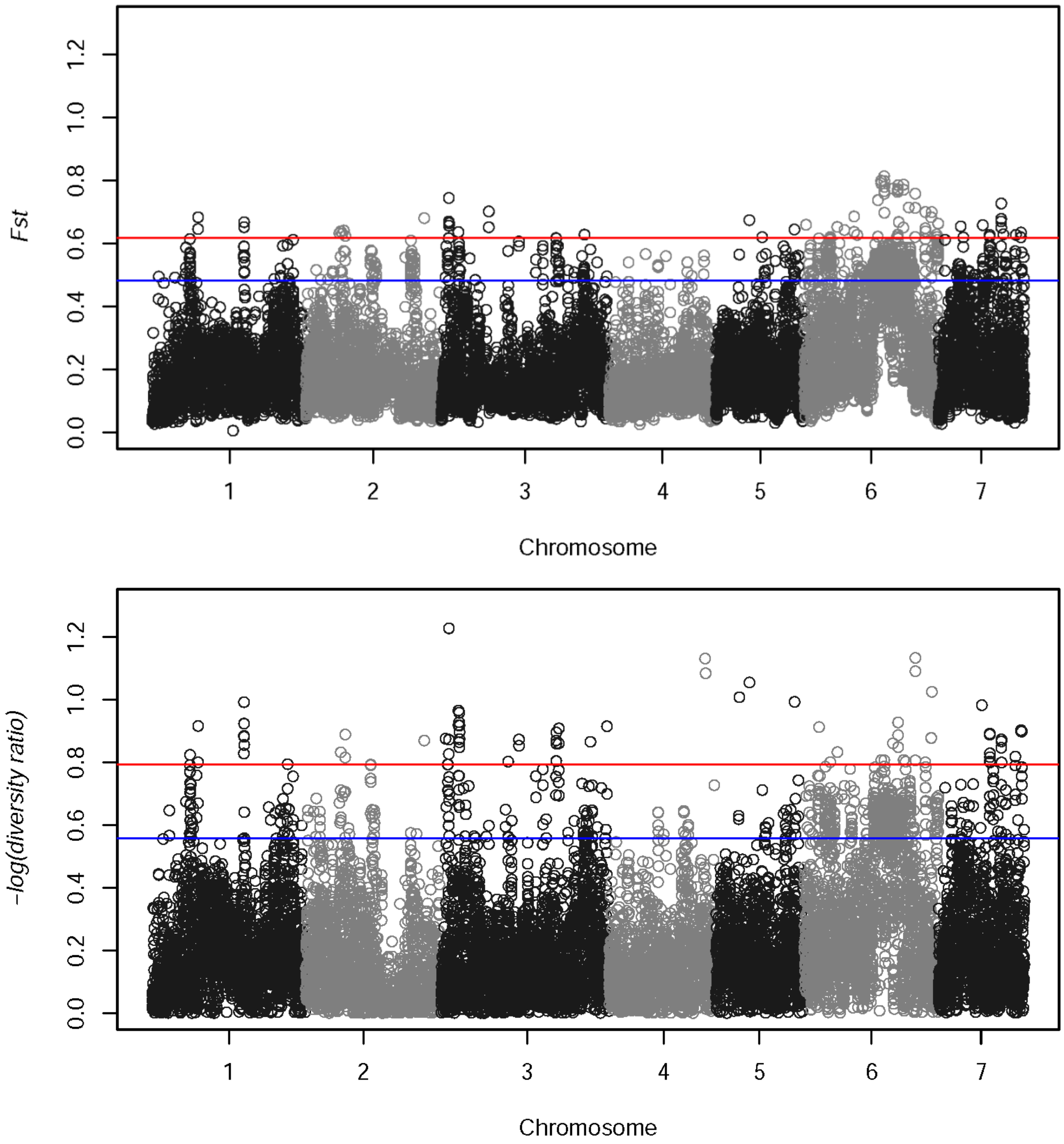

b.

Supplementary Figure 18. Diversity in PMiGAP lines and wild species accessions of pearl millet. a. Diversity metrics, presented as average pair-wise nucleotide diversity (current [θπ] and historical [θω]), are shown across all seven pseudomolecules. **b**. F_{ST} and -log ratio of the diversity loss across the 7 pseudomolecules.

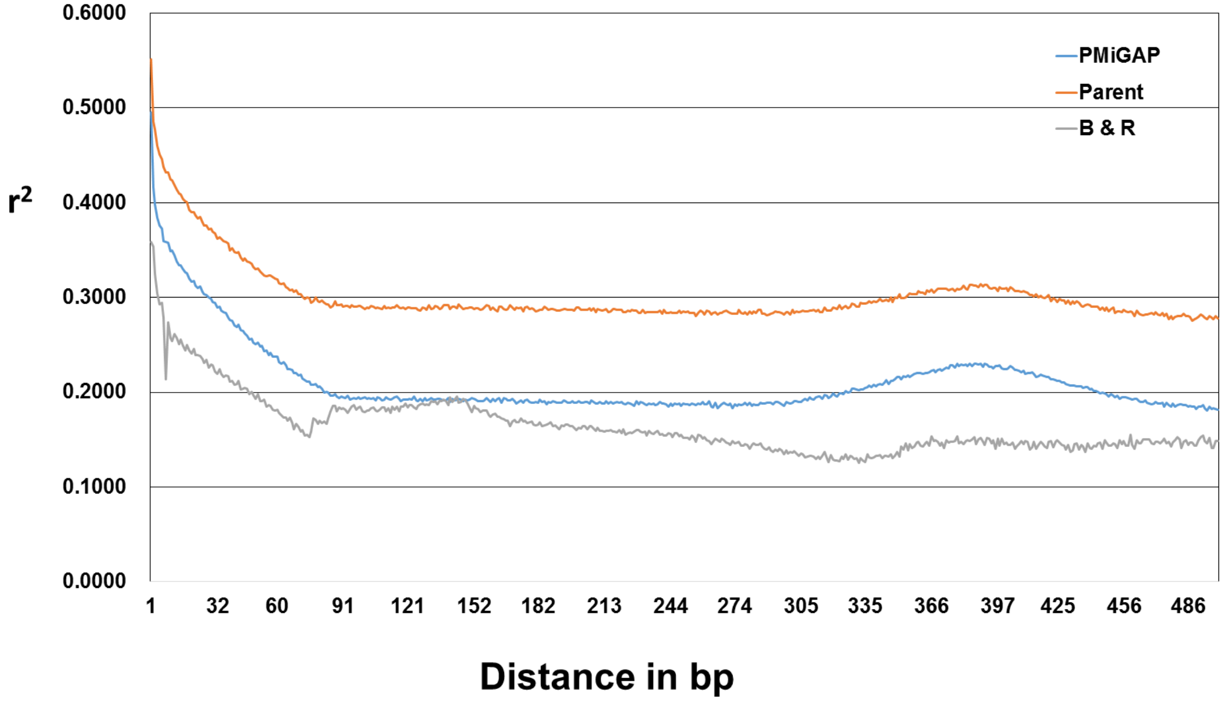

Supplementary Figure 19. Genome wide linkage disequilibrium decay in the PMiGAP lines, parents of mapping populations, and B- and R- lines. Linkage disequilibrium decays more rapidly across the PMiGAP lines compared to the B- and R- lines and parental lines.

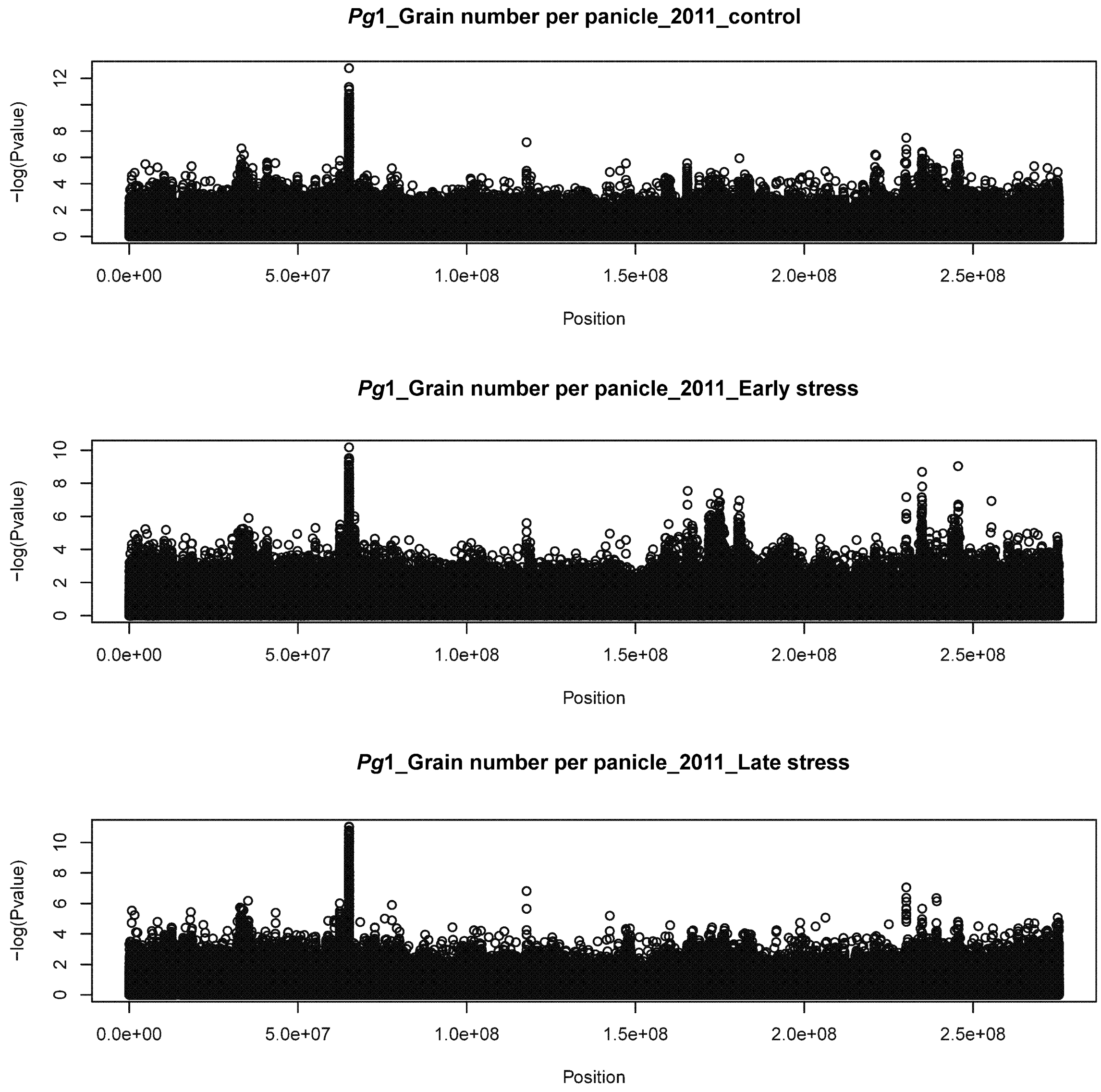

Pg1_Grain number per panicle_2011_control
−log(Pvalue)
0
2
4
6
8
12
0.0e+00
5.0e+07
1.0e+08
1.5e+08
2.0e+08
2.5e+08
Position
Pg1_Grain number per panicle_2011_Early stress
−log(Pvalue)
0
2
4
6
8
10
0.0e+00
5.0e+07
1.0e+08
1.5e+08
2.0e+08
2.5e+08
Position
Pg1_Grain number per panicle_2011_Late stress
−log(Pvalue)
0
2
4
6
8
10
0.0e+00
5.0e+07
1.0e+08
1.5e+08
2.0e+08
2.5e+08
Position

a.

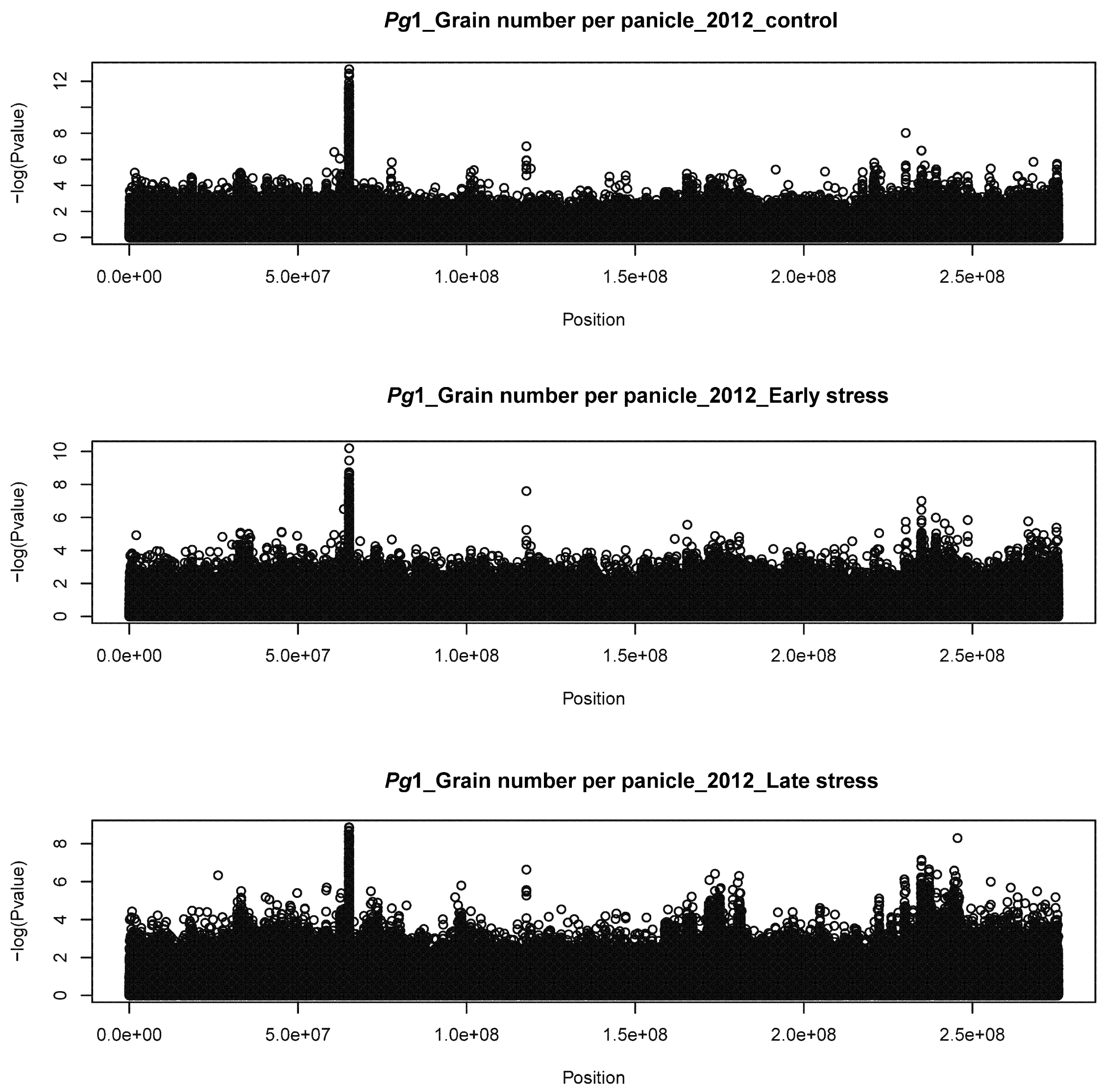

b.

Nature Biotechnology: doi:10.1038/nbt.3943

***Pg5*_Grain number per panicle_2011_control**

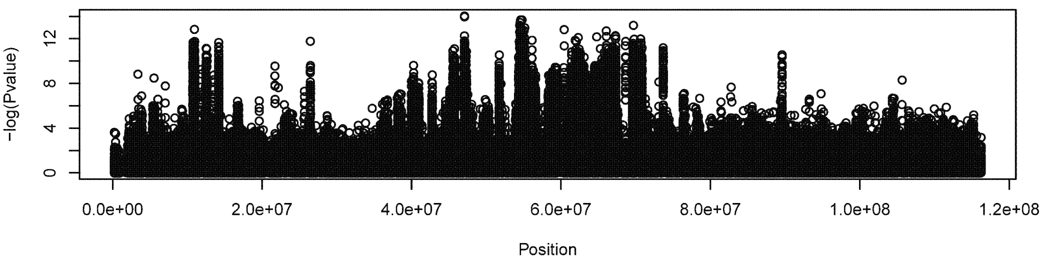

***Pg5*_Grain number per panicle_2011_Early stress**

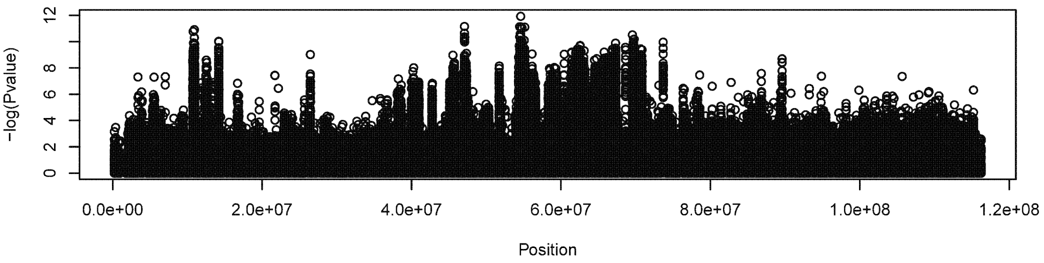

***Pg5*_Grain number per panicle_2011_Late stress**

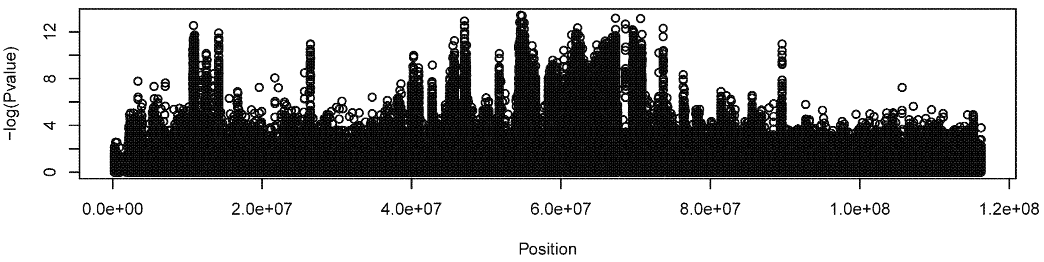

c.

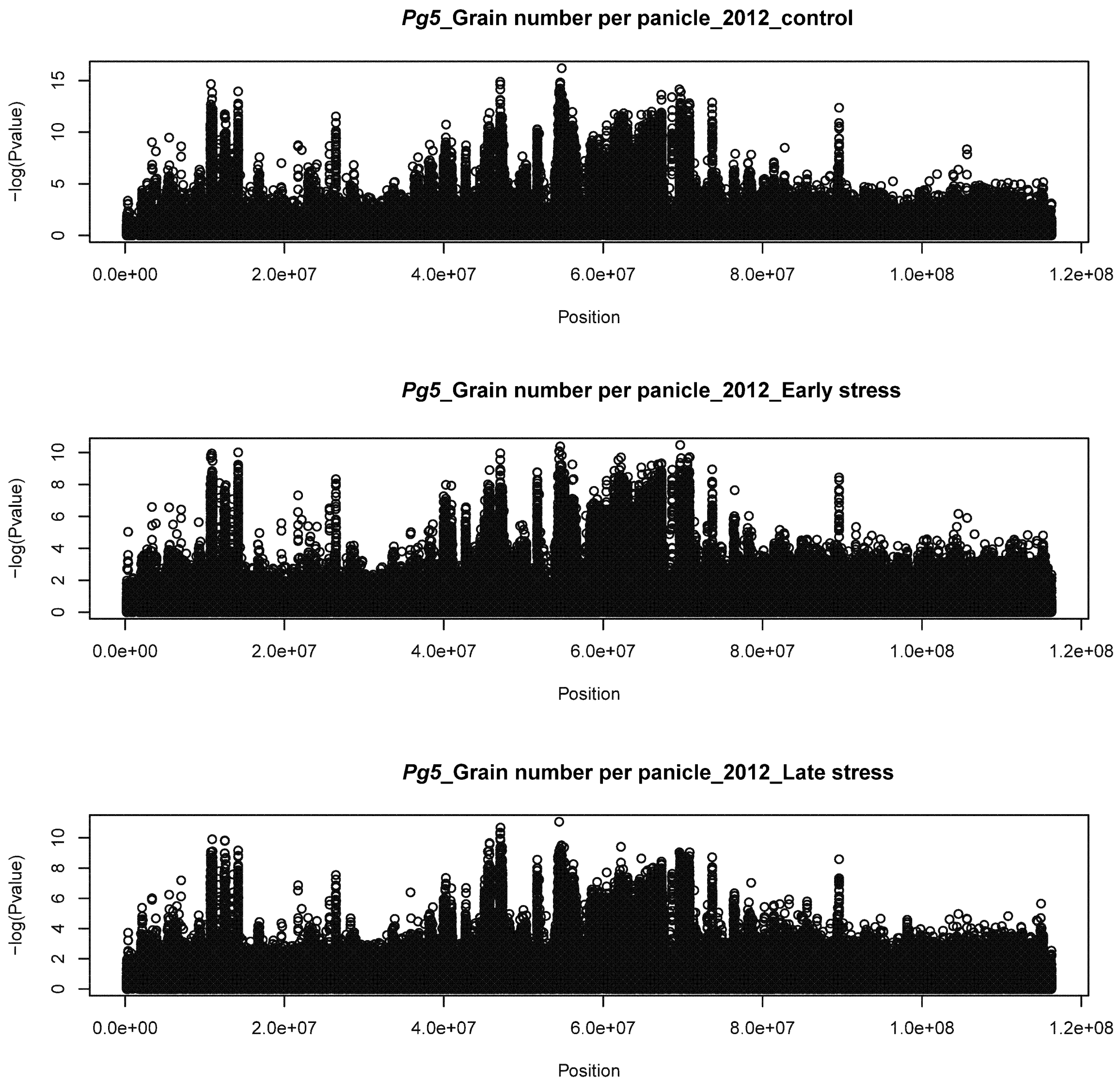

d.

Supplementary Figure 20. Association mapping for the grain number per panicle. The figure represent the -log(p-value) of the association in function of the position of the SNP on the pseudomolecule for the control as well as early and late stress field trials. We represented here the result for the grains per panicle (GNP). (a) results for *Pg*1 in 2011 , (b) results for *Pg*1 in 2012, (c) results for *Pg*5 in 2011, (d) results for *Pg*5 in 2012.

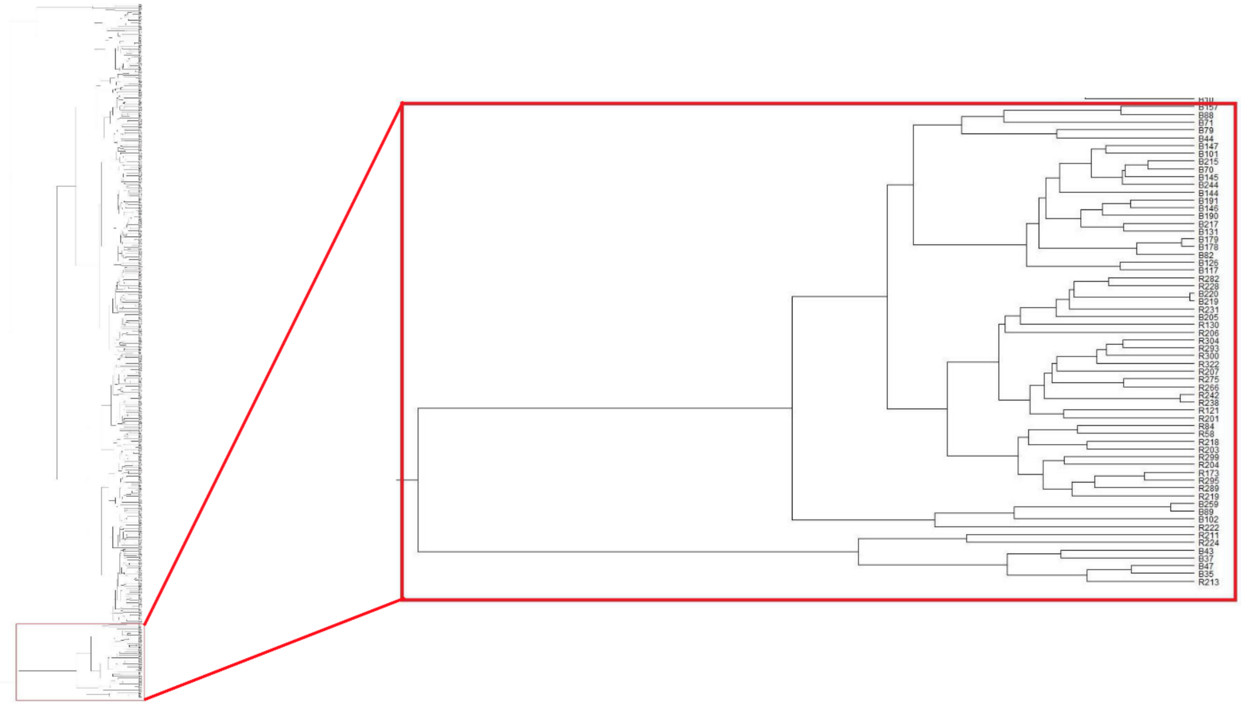

Supplementary Figure 21. Hierarchical clustering of predicted hybrid performance. The lower bottom of clusters shown are the best hybrid combinations for pearl millet breeding

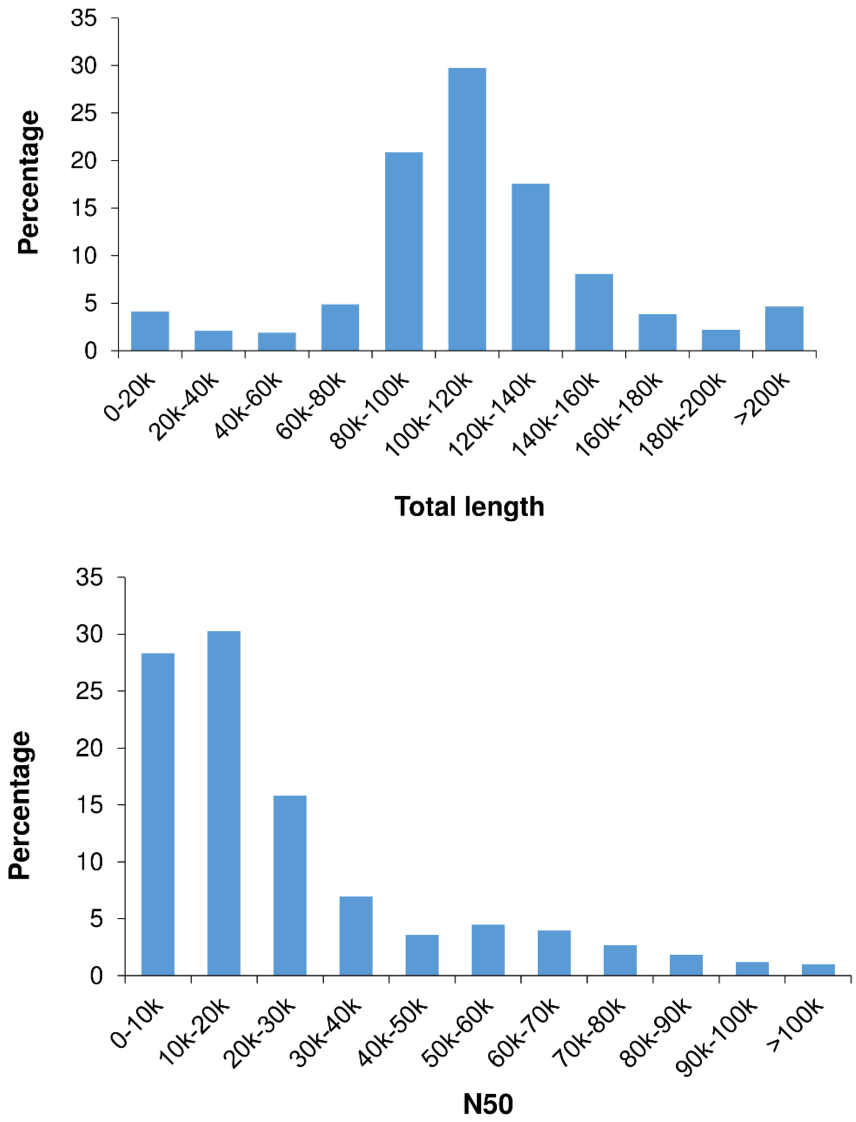

Supplementary Figure 22. Length and N50 distribution of the BAC clones.

Nature Biotechnology: doi:10.1038/nbt.3943

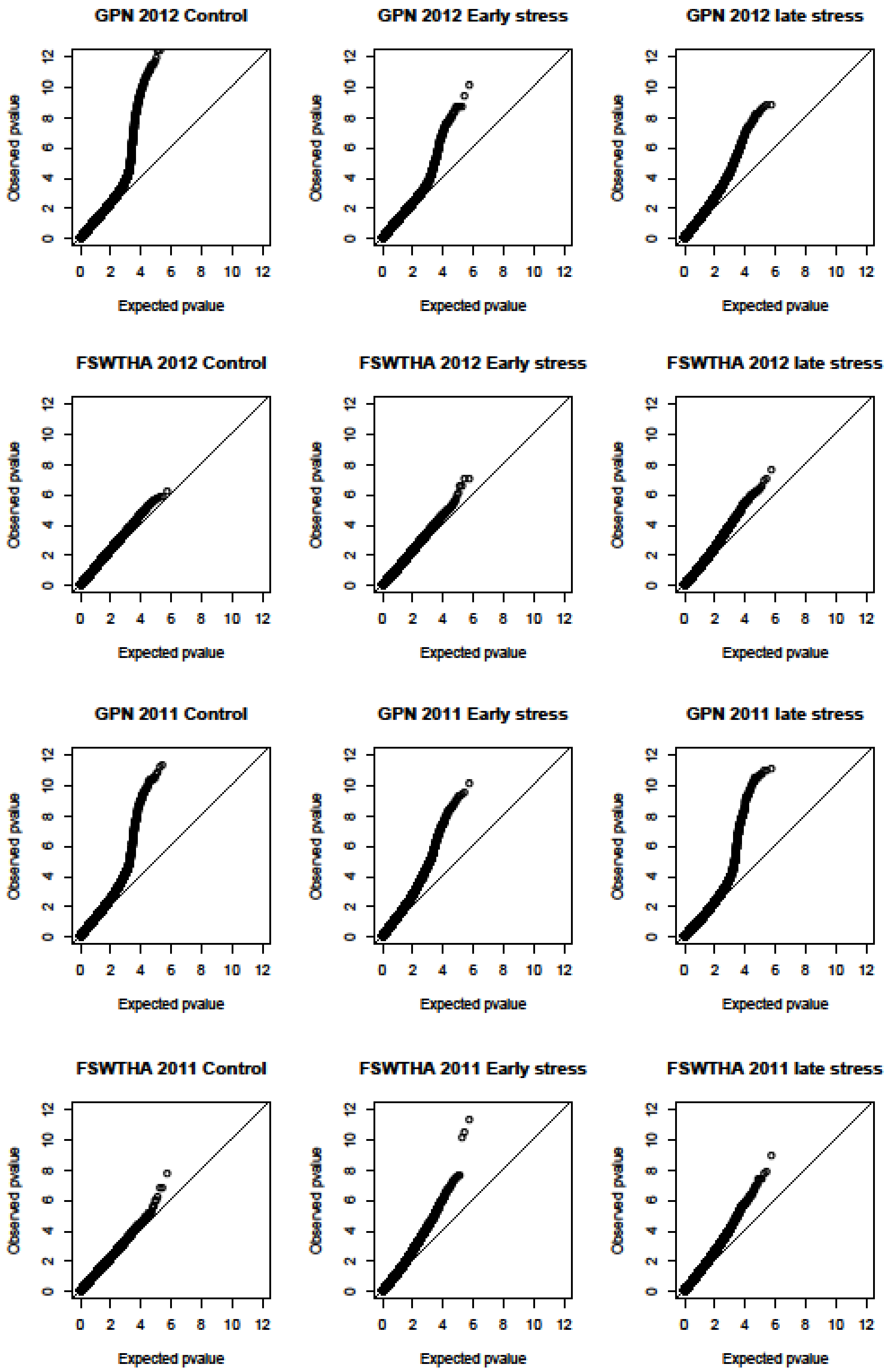

Supplementary Figure 23. QQ plot of expected and observed P-value. The expected and observed p-value for the number of grains per panicle (GNP) and fresh stover yield (FSWTHA) are reported for the two years (2011, 2012) and the three field trials (control, early stress, late stress). P-value are reported on a -log scale. A value of 4 corresponds to a P-value of 10^{-4}. Expected

Nature Biotechnology: doi:10.1038/nbt.3943

and observed p-value fit the x=y relationship. The marker trait association with are significant when P-value above 10^{-4}.

Nature Biotechnology: doi:10.1038/nbt.3943
