# Supplementary material for: Pearl millet genome sequence provides a resource to improve agronomic traits in arid environments
Source: Nat Biotechnol. 2017 Sep 18;35(10):969–76. doi: 10.1038/nbt.3943 (PMC6871012; doi:10.1038/nbt.3943)
Supplement: Life Sciences Reporting Summary — (PDF 114 kb) [file 41587_2017_BFnbt3943_MOESM48_ESM.pdf]

Corresponding author(s): Rajeev K Varshney

☐ Initial submission ☐ Revised version ☒ Final submission

Editorial Policy Checklist

This form is used to ensure compliance with Nature Research editorial policies related to research ethics and reproducibility in the life sciences. For further information, please see our Authors & Referees site. All questions on the form must be answered.

Data availability

Policy information about availability of data

Data availability statement

All manuscripts must include a data availability statement. This statement should provide the following information, where applicable:

- Accession codes, unique identifiers, or web links for publicly available datasets
- A list of figures that have associated source data
- A description of any restrictions on data availability

☒ A full data availability statement is included in the manuscript.

Required accession codes

Data deposition is mandated for certain types of data.

Confirm that all relevant data have been deposited into a public repository and that all accession codes are provided.

☐ Accession codes will be available before publication ☐ No data with mandated deposition ☒ All relevant accession codes are provided

Data presentation

Image integrity

☒ Confirm that all images comply with our image integrity policy.

Unprocessed data must be provided upon request. Please double-check figure assembly to ensure that all panels are accurate (e.g. all labels are correct, no inadvertent duplications have occurred during preparation, etc.).

Data distribution

Data should be presented in a format that shows data distribution (dot-plots or box-and-whisker plots), with all box-plot elements (e.g. center line, median; box limits, upper and lower quartiles; whiskers, 1.5x interquartile range; points, outliers) defined. If bar graphs are used, the corresponding dot plots must be overlaid.

☒ Confirm that all data presentation meets these requirements.

☐ Confirm that in all cases where the number of data points is <10, individual data points are shown.

Structural data

Policy information about special considerations for specific types of data

☒ If this study did not involve data of these types, check here and skip the rest of this section.

Electron microscopy

☐ For all electron microscopy work, confirm that you have deposited any density maps and coordinate data in EMDB.

Macromolecular structures

☐ For all macromolecular structures studied, confirm that you have provided an official validation report from wwPDB.

Code availability

Policy information about availability of computer code

Code availability statement

For all studies using custom code, the Methods section must include a statement under the heading "Code availability" describing how readers can access the code, including any access restrictions.

☒ A full code availability statement is included in the manuscript ☐ No custom code used

Nature Biotechnology: doi:10.1038/nbt.3943

▸ Research animals

Policy information about studies involving animals; follow the ARRIVE guidelines for reporting animal research

☒ If this study did not use animals and/or animal-derived materials for which ethical approval is required, check here and skip the rest of this section.

Ethical compliance

☐ Confirm that you have complied with all relevant ethical regulations and that a statement affirming this is included in the manuscript.

Ethics committee

☐ Confirm that you have stated the name(s) of the board and institution that approved the study protocol in the manuscript.

▸ Human research participants

Policy information about studies involving human research participants

☒ If this study did not involve any human research participants, check here and skip the rest of this section.

Ethical compliance

☐ Confirm that you have complied with all relevant ethical regulations and that a statement affirming this is included in the manuscript.

Ethics committee

☐ Confirm that you have stated the name(s) of the board and institution that approved the study protocol in the manuscript.

Informed consent

☐ Confirm that informed consent was obtained from all participants.

Identifiable images

For publication of identifiable images of research participants, confirm that consent to publish was obtained and is noted in the Methods.

Authors must ensure that consent meets the conditions set out in the Nature Research participant release form.

☐ Yes ☐ No identifiable images of human research participants

▸ Clinical studies

Policy information about clinical studies

☒ If this study was not a clinical trial, check here and skip the rest of this section.

Clinical trial registration

☐ Confirm that you have provided the trial registration number from ClinicalTrials.gov or an equivalent agency in the manuscript.

Phase 2 and 3 randomized controlled trials

Confirm that you have provided the CONSORT checklist with your submission.

☐ Yes ☐ No ☐ Not a phase 2/3 randomized controlled trial

Tumor marker prognostic studies

Did you follow the REMARK reporting guidelines?

☐ Yes ☐ No ☐ Not a tumor marker prognostic study

▸ Methods reporting

Nature Research wishes to improve the reproducibility of the work we publish. As part of this effort, all life science manuscripts require a reporting summary; certain types of research require specialized modules in addition to this form.

☒ Confirm that you have provided a complete and accurate reporting summary.

n/a	Confirmed
☒	☐ For MRI studies, confirm that you have completed the additional MRI module.
☒	☐ For flow cytometry studies, confirm that you have completed the additional flow cytometry module.
☒	☐ For ChIP-seq studies, confirm that you have completed the additional ChIP-seq module.

I certify that all the above information is complete and correct.

Typed signature Rajeev K Varshney Date 11/07/2017

Nature Biotechnology: doi:10.1038/nbt.3943
