# Supplementary material for: Pearl millet genome sequence provides a resource to improve agronomic traits in arid environments
Source: Nat Biotechnol. 2017 Sep 18;35(10):969–76. doi: 10.1038/nbt.3943 (PMC6871012; doi:10.1038/nbt.3943)
Supplement: Supplementary Tables — 1–14, 16–18, 22, 24, 28, 32–33, 42. (PDF 1754 kb) [file 41587_2017_BFnbt3943_MOESM49_ESM.pdf]

Supplementary Tables

Supplementary Table 1. Summary of whole genome shotgun sequence used for genome assembly

Library insert size (bp)	Read length (bp)*	Raw data		Filtered data	
		Total data (Gb)	Sequence depth (X)	Total data (Gb)	Sequence depth (X)
170	100PE	86.60	49.28	78.64	44.75
250	150PE	88.43	50.32	63.07	35.89
500	90PE	58.32	33.19	50.63	28.81
800	100PE	56.81	32.32	47.43	26.99
2,000	49PE	83.06	47.26	37.22	21.18
5,000	49PE	73.95	42.08	22.21	12.64
10,000	49PE	35.32	20.10	7.26	4.13
20,000	49PE	21.18	12.15	2.10	1.19
40,000	49PE	16.52	9.40	3.42	1.95
**Total**		**520.19**	**296.00**	**311.97**	**177.52**

***PE= paired end**

Nature Biotechnology: doi:10.1038/nbt.3943

Supplementary Table 2. Statistics of bacterial artificial chromosome (BAC) sequencing

Library insert size (bp)*	Number of BACs	Total data (Gb)	Raw mean depth (X)/ BAC	Clean mean depth (X)/ BAC
500	100,608	971.51	80.47	60.68

*The average size of BAC clones is 120 kb

Nature Biotechnology: doi:10.1038/nbt.3943

Supplementary Table 3. Statistics of the final genome assembly

	**Contig**		**Scaffold (≥1 kb)**	
	Size	Number	Size	Number
N90	3.99 kb	95,122	182.47 kb	2,261
N80	7.26 kb	66,462	359.87 kb	1,572
N70	10.1 kb	48,105	529.31 kb	1,157
N60	13.73 kb	34,700	695.61 kb	857
**N50**	**18.18 kb**	**175,708**	**884.95 kb**	**25,241**
Longest	282.9 kb	-	4.82 Mb	-
Total size	1.58 Gb	-	**1.79 Gb**	-
Total number (>=100 bp)	208,873		-	57,931
Total number (>=2 kb)	121,670		-	10,605

Nature Biotechnology: doi:10.1038/nbt.3943

Supplementary Table 4. Genome sequence assembly organized in chromosome-level pseudomolecules

Pseudomolecule name	Number of scaffolds in pseudomolecule	Pseudomolecule size (bp)
Pg1	1138	275,469,374
Pg2	815	243,054,348
Pg3	963	300,907,625
Pg4	809	191,830,058
Pg5	699	158,694,342
Pg6	894	240,570,548
Pg7	561	154,011,256
**Total**	**5,879**	**1,564,537,551**

Nature Biotechnology: doi:10.1038/nbt.3943

Supplementary Table 5. Summary of whole genome GC content and expanded gene families.

Species	GC%（whole CDS）	GC%（expanded families）
Pearl millet	54.76	53.14
Foxtail millet	54.93	53.91
Sorghum	55.17	53.93
Barley	54.54	53.57
Rice	55.32	52.91

Nature Biotechnology: doi:10.1038/nbt.3943

Supplementary Table 6. Evaluation of completeness of the genome assembly using the core eukaryotic gene mapping approach (CEGMA)

Parameter*		Number	Percent (%)
Total KOGs		450	
KOGs aligning to single genes		437	97.11
KOGs aligning to several genes	Overlap >0.5	5	1.11
KOGs without aligned genes		8	1.78

*KOGs=Eukaryotic orthologous gene sequences

We found 97.11% of the eukaryotic orthologous gene sequences and 5 KOGs mapping to two or more genes in our assembly, i.e. 1.11% of the KOG genes. Manual visualization of these 5 genes show split into 10 genes in our assembly. Consequently we could estimate the percentage of the gene model split in our assembly to 2.3% i.e. ten genes out of 437+5 are split.

Nature Biotechnology: doi:10.1038/nbt.3943

Supplementary Table 7. Summary of Class I transposable elements in the current assembly

Category	Percent in the assembly (%)
Class I	
LTR	60.0
Copia	22.2
Gypsy	28.8
Unclassified LTR Retros	9.0
LINE	0.11
SINE	0.004

Nature Biotechnology: doi:10.1038/nbt.3943

Supplementary Table 8. Comparison of different pearl millet transcriptome assemblies

Feature*	Transcriptome assemblies			
	Comprehensive (this study)	Zeng et al.[15]	Rajaram et al.[16]	This study‡
Sequencing platform	Mixed	454 GS FLX	454 GS FLX	PacBio
Program used for assembly	CAP3	MIRA	PLANTTA/TGICL	NA
Total number of TACs*	69,398 (15,521 contigs + 53,877 singletons)	26,576 (26,498 contigs + 78 singletons)	112,864 (34,270 contigs +78,594 singletons)	50,313
Total number of bases (bp)	43,011,379	6,832,261	26,843,578	36,479,993
Average contig length (bp)	591	257	238	725
N50 (bp)	1014	293	238	1142
Largest contig (bp)	15,300	2770	2571	15,292
Shortest contig (bp)	200	40	100	100

*TACs- transcriptome assembled contigs

‡The transcriptome data can be accessed at https://sites.google.com/site/africropproject/data

Nature Biotechnology: doi:10.1038/nbt.3943

Supplementary Table 9. Prediction of protein-coding genes in pearl millet

Gene set		Number	Average transcript length (bp)	Average CDS length (bp)	Average number of exons per gene	Average exon length (bp)	Average intron length (bp)
De novo	AUGUSTUS	53,274	2133.76	712.37	3.11	229.34	674.87
	FGENESH	53,689	3107.46	944.47	4.31	218.99	652.90
Homolog	Rice	26,187	2484.78	1139.00	4.85	234.64	349.17
	Foxtail millet	34,417	2307.77	1103.26	4.58	241.05	336.75
	Sorghum	28,353	2481.16	1136.16	4.79	237.18	354.86
	Maize	33,208	2309.73	1051.49	4.67	225.13	342.79
PgTA		52,758	3945.35	687.59	2.84	242.22	1771.79
GLEAN		38,579	2420.19	1014.71	4.09	248.06	454.77

PgTA= *Pennisetum glaucum* transcriptome assembly. For predictions with Augustus (Version 2.5.5) and FGENESH (Version 1.3), parameters trained on maize and monocot plants were used, respectively, with other parameters setting as default. For homolog-based prediction, proteins from four monocot species, *O. sativa* (JGI9.0), *S. italica* (http://foxtailmillet.genomics.org.cn/), *S. bicolor* (JGI7.0) and *Z. mays* (JGI9.0) were aligned to the pearl millet genome with BLAT to find homologous regions. GeneWise (version 2.2) was then used to generate spliced alignments and the gene models. PgTA sequences were matched against the genome with BLAT with identity ≥0.98, coverage ≤0.98 to find coding region with high confidence. Gene models predicted from the three approaches mentioned above were integrated using GLEAN (version) to produce the final consensus gene set.

Nature Biotechnology: doi:10.1038/nbt.3943

Supplementary Table 10. Functional annotation of predicted genes in pearl millet

	Number	Percent (%)
Total	38,579	
Functionally annotated	27,893	72.30
SwissProt	21,455	55.61
InterPro	25,281	65.53
KEGG	21,526	55.80
Unannotated	10,686	27.70

Versions: KEGG81, UniProt rls201506 and InterProScan 5

Nature Biotechnology: doi:10.1038/nbt.3943

Supplementary Table 11. Orthology of pearl millet proteins with the proteins of Arabidopsis, banana, barley, Brachypodium, foxtail millet, maize, rice, sorghum, bread wheat and soybean

Species	Pearl millet		Aligned species	
	**Matched proteins**	**Percentage (%)**	**Matched proteins**	**Percentage (%)**
Pearl millet vs Arabidopsis	23,874	61.88	22,005	82.61
Pearl millet vs banana	24,356	63.13	29,610	81.06
Pearl millet vs barley	26,932	69.81	32,526	40.98
Pearl millet vs Brachypodium	27,106	70.26	23,951	90.68
Pearl millet vs foxtail millet	28,609	74.16	28,232	84.87
Pearl millet vs maize	27,355	70.91	31,271	78.55
Pearl millet vs rice	27,372	70.95	27,415	70.40
Pearl millet vs sorghum	27,726	71.87	25,716	94.69
Pearl millet vs soybean	24,005	62.22	38,877	90.71
Pearl millet vs bread wheat	26,197	67.90	27,268	78.18

The alignment was performed by BlastP with an e-value threshold of 1.0e-05. The values in "Matched proteins" show the number of proteins with a match

Nature Biotechnology: doi:10.1038/nbt.3943

Supplementary Table 12. Orthology analysis at the protein level of predicted genes in pearl millet with those annotated in Arabidopsis, banana, barley, Brachypodium, foxtail millet, maize, rice, sorghum, bread wheat and soybean

Species	Total predicted genes	No. of genes in orthologous groups	No. of genes not in orthologous groups [1]	Total No. of orthologous groups[2]	Species-specific homolog groups[3]	Average No. of genes per group
**Pearl millet**	**38,579**	**30,783**	**7,796**	**17,949**	**800**	**1.72**
Arabidopsis	26,637	22,845	3,792	13,064	860	1.75
Brachypodium	26,413	23,335	3,078	17,198	232	1.36
Banana	36,549	25,585	10,964	12,759	709	2.01
Barley	79,379	33,628	45,751	20,715	2,711	1.62
Foxtail millet	33,264	27,327	5,937	19,156	204	1.43
Maize	39,810	30,851	8,959	18,509	1,355	1.67
Rice	38,942	27,913	11,029	18,627	1,103	1.50
Sorghum	27,159	24,466	2,693	18,339	83	1.33
Soybean	42,859	38,648	4,211	14,036	1,508	2.75
Bread wheat	34,879	25,563	9,316	16,858	838	1.52

[1]Predicted genes that were not organized into groups using OrthoMCL. We suggest that many such genes are mis-annotated, though we cannot rule out genes with unique domain arrangements that have undergone lineage specific expansion. [2]Orthologous groups containing at least one gene from the indicated species. [3]Groups containing putative paralogs from the indicated species, but lacking genes from other species. Such unassigned homologous groups may contain genes with ambiguous relationships among species, such as many of the NBS-LRR disease resistance genes that can evolve by processes such as non-allelic recombination and gene conversion.

Nature Biotechnology: doi:10.1038/nbt.3943

Supplementary Table 13. Summary of orthologous and paralogous genes in pearl millet relative to selected sequenced cereal, legume and dicot genomes

Species	**Single-copy orthologs**	**Co-orthologs[1] (Multiple copy orthologs)**	**Unique paralogs**	**Other orthologs[2]**	**Unclustered genes**
**Pearl millet**	**5,232**	**5,205**	**7,002**	**13,344**	**7,796**
Arabidopsis	4,609	6,923	3,338	7,975	3,792
Banana	3,578	11,528	1,874	8,605	10,964
Barley	4,603	7,108	7,164	14,753	45,751
Brachypodium	5,106	5,355	616	12,258	3,078
Foxtail millet	4,894	6,168	510	15,755	5,937
Maize	4,058	8,554	4,025	14,214	8,959
Rice	5,061	5,707	3,335	13,810	11,029
Sorghum	4,995	5,758	216	13,497	2,693
Soybean	1,326	19,031	4,489	13,802	4,211
Bread wheat	5,435	4,905	2,499	12,724	9,316

[1]Co-orthologous genes, also known as "inparalogs", are derived from duplication in the indicated genome. [2]Other orthologs represent gene duplication events internal to the overall set, but basal to more than two of the compared species.

Nature Biotechnology: doi:10.1038/nbt.3943

Supplementary Table 14. Non-coding RNA genes in the pearl millet genome

Type		Number of copies	Average length (bp)	Total length (bp)	% of genome
miRNA		183	125.51	22,968	0.000013
tRNA		909	75.86	68,961	0.000038
rRNA		235	265.70	62,440	0.000034
	18S	64	651.33	41,685	0.000023
	28S	78	133.31	10,398	0.000006
	5.8S	26	149.50	3,887	0.000002
	5S	67	96.57	6,470	0.000004
snRNA		752	119.04	89,519	0.000049
	CD-box	579	110.03	63,705	0.000035
	HACA-box	28	121.07	3,390	0.000002
	splicing	145	154.65	22,424	0.000012

Nature Biotechnology: doi:10.1038/nbt.3943

Supplementary Table 15. Summary of genes expanded during pearl millet evolution

It has been uploaded as an excel spread sheet

Nature Biotechnology: doi:10.1038/nbt.3943

Supplementary Table 16. Different classes of nucleotide binding site (NBS) encoding genes present in pearl millet and other monocot species

	Pearl millet	Foxtail millet	Sorghum	Rice
	**Number (%)**	**Number (%)**	**Number (%)**	**Number (%)**
LRR-NBS-LRR	1 (0.26)	1 (0.22)	0	1 (0.19)
NBS-only	154 (40.74)	126 (27.94)	99 (34.49)	192 (36.02)
NBS-LRR	162 (42.86)	223 (49.46)	145 (50.52)	267 (50.09)
NBS (X2,3)	39 (10.32)	51 (11.31)	23 (8.01)	44 (8.26)
NBS (X2,3)-LRR	22 (5.82)	50 (11.09)	20 (6.98)	29 (5.44)
NBS-total	**378**	45	287	533
TIR-only	2	2	2	1

Nature Biotechnology: doi:10.1038/nbt.3943

Supplementary Table 17. Distribution of nucleotide binding site (NBS) encoding genes on the seven pseudomolecules of pearl millet

Pseudomolecule name	NBS number	NBS (%)	Genes on pseudomolecule	NBS on each pseudomolecule (%)
Pg1	97	25.66	5,925	1.64
Pg2	33	8.73	6,039	0.55
Pg3	25	6.61	5,979	0.42
Pg4	99	26.19	3,992	2.48
Pg5	30	7.94	4,669	0.64
Pg6	23	6.08	5,113	0.45
Pg7	53	14.03	4,074	1.30
Unanchored scaffolds	18	4.76	2,788	0.65
**Total**	**378**	**100**	**38,579**	

Nature Biotechnology: doi:10.1038/nbt.3943

Supplementary Table 18. **Summary of tandem/closely located groups of NBS genes on seven pseudomolecules of pearl millet**

Pseudo-molecule	Total NBS gene	Tandem (1)*	Closely located group(5)*	Closely located group(10)*
Pg1	97	11X2**, 1X3, 2X4	8X2, 7X3, 3X4, 3X5, 1X6	6X2, 3X3, 3X4, 6X5, 2X6
Pg2	33	2X2	4X2, 1X3	5X2, 1X3
Pg3	25	2X2, 1X3, 1X4	2X2, 1X3, 2X4	2X2, 1X3, 1X4, 1X5
Pg4	99	11X2, 2X3, 3X4	9X2, 3X3, 6X4, 1X5	12X2, 3X3, 6X4, 2X6
Pg5	30	2X2, 1X5	3X2, 1X4, 1X6	3X2, 1X4, 1X7
Pg6	23	2X3	2X2, 1X3, 1X4	2X2, 1X3, 1X4
Pg7	53	8X2, 1X3	6X2, 2X3, 1X4	7X2, 1X3, 2X4

*Three types of tandem/closely located NBS gene groups were defined and identified: (a) Tandem (1) - are groups of NBS genes that are strictly adjacent with each other; (b) Tandem (5) -Closely located group of NBS genes that are within 5 non-NBS genes and (c) Tandem (10) -Closely located group of NBS genes that are within 10 non-NBS genes

**pattern of group: e.g. 11X2 means there are 11 groups of tandem (1) with size = 2 genes in Pg1

Specifically, seven closely located tandem (10) group were found within the first 10 Mb region of Pg1(two 4-gene groups, four 5-gene groups and one 6-gene group), while on Pg4 there were five closely located tandem (10) group in the first 20 Mb region (three 2-gene groups and two 4-gene groups)

Nature Biotechnology: doi:10.1038/nbt.3943

Supplementary Table 19. Summary of data generated on the PMiGAP lines using whole genome resequencing
It has been uploaded as an excel spread sheet

Nature Biotechnology: doi:10.1038/nbt.3943

Supplementary Table 20. Data generated on 38 inbred parents of different mapping populations using whole genome resequencing

It has been uploaded as an excel spread sheet.

Nature Biotechnology: doi:10.1038/nbt.3943

Supplementary Table 21. Data generated for B- and R-lines of pearl millet using RAD-Seq approach

It has been uploaded as an excel spread sheet.

Nature Biotechnology: doi:10.1038/nbt.3943

Supplementary Table 22. Summary of whole genome resequencing data generated on 31 wild pearl millet accessions

Sample ID	Raw reads	Raw bases (bp)	Clean reads	Clean bases (bp)	Depth (X)
PE00838	20,991,644	4,072,378,936	19,327,391	3,749,513,854	2.06
PE01458	21,645,848	4,199,294,512	19,558,592	3,794,366,848	2.09
PE05720	26,744,557	5,188,444,058	24,616,203	4,775,543,382	2.63
PE05722	17,071,580	3,311,886,520	15,789,265	3,063,117,410	1.69
PE05724	21,989,652	4,265,992,488	19,807,750	3,842,703,500	2.12
PE08084	18,266,209	3,543,644,546	17,052,862	3,308,255,228	1.82
PE08094	15,059,662	2,921,574,428	13,715,527	2,660,812,238	1.46
PE08106	20,341,921	3,946,332,674	18,563,412	3,601,301,928	1.98
PE08127	19,312,626	3,746,649,444	17,683,366	3,430,573,004	1.89
PE08136	15,116,320	2,932,566,080	14,147,046	2,744,526,924	1.51
PE08146	24,855,946	4,822,053,524	23,064,451	4,474,503,494	2.46
PE08155	11,762,336	2,281,893,184	10,962,258	2,126,678,052	1.17
PE08165	30,798,488	5,974,906,672	27,840,516	5,401,060,104	2.97
PE08171	25,929,183	5,030,261,502	23,939,935	4,644,347,390	2.56
PE08470	41,205,101	7,993,789,594	37,285,934	7,233,471,196	3.98
PE08473	14,886,036	2,887,890,984	13,767,576	2,670,909,744	1.47
PE08492a	25,492,041	4,945,455,954	23,127,743	4,486,782,142	2.47
PE08487	15,807,544	3,066,663,536	14,378,064	2,789,344,416	1.54
PE08492	18,626,253	3,613,493,082	17,248,236	3,346,157,784	1.84
PE08498	18,965,000	3,679,210,000	17,275,954	3,351,535,076	1.85
PE08503	16,846,817	3,268,282,498	15,216,794	2,952,058,036	1.63
PE08721	24,132,585	4,681,721,490	22,191,406	4,305,132,764	2.37
PE08726	20,379,802	3,953,681,588	18,755,512	3,638,569,328	2.00
PE08727	26,464,489	5,134,110,866	24,312,948	4,716,711,912	2.60
PE08730	21,223,323	4,117,324,662	19,458,645	3,774,977,130	2.08
PE08731	19,248,865	3,734,279,810	17,746,151	3,442,753,294	1.90
PE08732	22,697,013	4,403,220,522	20,958,165	4,065,884,010	2.24
PE08734	25,386,251	4,924,932,694	23,018,094	4,465,510,236	2.46
PE08736	20,799,867	4,035,174,198	19,193,426	3,723,524,644	2.05
PE08743	9,334,258	1,810,846,052	8,607,858	1,669,924,452	0.92
PE08744	31,308,056	6,073,762,864	28,511,787	5,531,286,678	3.05

Nature Biotechnology: doi:10.1038/nbt.3943

Supplementary Table 23. Summary of SSR motifs identified, primers designed and their genome coordinates

It has been uploaded as an excel spread sheet.

Nature Biotechnology: doi:10.1038/nbt.3943

Supplementary Table 24. Summary statistics on the simple sequence repeats identified, their distribution and primer design based on genome assembly

Total size of examined sequences (bp)	1,816,969,981
Total number of identified SSRs	88,256
Total number of SSRs present in compound formation	9,042
Number of scaffolds examined	52,052
Number of SSR containing scaffolds	3,411
Number of scaffolds containing more than 1 SSR	840
Distribution to different repeat type classes (excluding mono-nucleotide repeats)	
Number of di-nucleotide repeats	39,394
Number of tri-nucleotide repeats	38,987
Number of tetra-nucleotide repeats	7,799
Number of penta-nucleotide repeats	1,347
Number of hexa-nucleotide repeats	729
Primer pairs for SSRs	
Scaffolds used to design primer pairs	1,729
Total numbers of primer pairs designed	74,891

*Minimum of six units for di-, and five units for tri-, tetra-, penta- and hexa-nucleotide repeats were required for defining simple SSRs. Primers were designed for identified SSRs using the software Primer3 v2.3.4 with the following criteria: (i) primer length ranging from 18 bp to 24 bp with an optimum of 20 bp (ii) product size ranging from 100 bp to 350 bp; (iii) melting temperature (Tm) between 50–65 °C with 60 °C as optimum; (iv) GC % content in the range 40–60%.

Nature Biotechnology: doi:10.1038/nbt.3943

Supplementary Table 25. Distribution of SNPs in intra-genic and inter-genic regions across PMiGAP lines

It has been uploaded as an excel spread sheet.

Nature Biotechnology: doi:10.1038/nbt.3943

Supplementary Table 26. Distribution of SNPs in intra-genic and inter-genic regions across parental lines of mapping populations

It has been uploaded as an excel spread sheet.

Nature Biotechnology: doi:10.1038/nbt.3943

Supplementary Table 27. Distribution of SNPs in intra-genic and inter-genic regions across B- and R- lines

It has been uploaded as an excel spread sheet.

Nature Biotechnology: doi:10.1038/nbt.3943

Supplementary Table 28. Distribution of SNPs and Indels across the seven pseudomolecules and unanchored scaffolds in the PMiGAP lines, parental lines of mapping populations and B- and R- lines of pearl millet

Pseudo-molecule	PMiGAP lines			Parental lines			B- and R- lines		
	SNPs	SNP density	Indels	SNPs	SNP density	Indels	SNPs	SNP density	Indels
Pg1	4,506,009	16,358	618,602	1,979,895	7,187	184,602	445,740	1,618	115,872
Pg2	4,089,497	16,825	583,597	1,928,005	7,932	194,296	380,052	1,564	106,950
Pg3	5,018,746	16,679	585,835	2,124,996	7,062	172,275	480,301	1,596	101,597
Pg4	3,614,768	18,844	476,029	1,557,206	8,118	126,269	383,674	2,000	86,562
Pg5	2,492,716	15,708	399,561	1,105,708	6,968	126,265	244,140	1,538	74,959
Pg6	3,957,457	16,450	453,547	1,320,771	5,490	127,486	353,157	1,468	78,949
Pg7	2,602,833	16,900	361,197	1,101,575	7,153	100,934	268,555	1,744	66,056
Pg0	3,260,147		366,078	1,070,978		79,418	305,089		62,786
Total	29,542,173		3,844,446	12,189,134		1,111,545	2,860,708		693,731

Pg0 indicates unanchored scaffolds

Nature Biotechnology: doi:10.1038/nbt.3943

Supplementary Table 29. Insertions and deletions identified in the PMiGAP lines

It has been uploaded as an excel spread sheet.

Nature Biotechnology: doi:10.1038/nbt.3943

Supplementary Table 30. Insertions and deletions identified in the parental lines of mapping populations

It has been uploaded as an excel spread sheet.

Nature Biotechnology: doi:10.1038/nbt.3943

Supplementary Table 31. Insertions and deletions identified in B- and R- lines

It has been uploaded as an excel spread sheet.

Nature Biotechnology: doi:10.1038/nbt.3943

Supplementary Table 32. Summary of structural variations in the PMiGAP lines, parental lines of mapping populations and B and R-lines of pearl millet

Germplasm	Type of variation*	Number of variants	Total length (kb)	Average length (kb)
PMiGAP	DEL	150,731	424,588.46	2.82
	INS	257,256	53,281.54	0.21
	INV	2,139	140,934.53	65.89
	ITX	12,992	158,904.77	12.23
Parental lines	DEL	75,822	280,024.25	3.69
	INS	29,673	6,892.51	0.23
	INV	704	58,457.37	83.04
	ITX	5,032	70,050.52	13.92
B- and R-lines	DEL	4,684	33,532.59	7.16
	INS	15,652	3,080.10	0.20
	INV	176	28,261.41	160.58
	ITX	4,849	19,782.18	4.08

*DEL, deletion; INS, insertion; INV, inversion; ITX, intra-chromosomal translocation

Nature Biotechnology: doi:10.1038/nbt.3943

Supplementary Table 33. Diversity levels in cultivated (pearl millet inbred germplasm association panel PMiGAP) and wild accessions of pearl millet

Pseudomolecule name	PMiGAP lines		Wild		
	**Theta Pi (θπ)**	**Theta w (θω)**	**Theta Pi (θπ)**	**Theta w (θω)**	**F_{ST}**
Pg1	0.00229	0.00283	0.00347	0.003346	0.18602
Pg2	0.0026	0.00287	0.00345	0.003371	0.18249
Pg3	0.00241	0.00288	0.00355	0.00336	0.1829
Pg4	0.00274	0.00316	0.00383	0.003599	0.15983
Pg5	0.00231	0.00271	0.00343	0.003313	0.19348
Pg6	0.002	0.00286	0.00403	0.003412	0.31023
Pg7	0.00241	0.00289	0.00396	0.003611	0.22735
Average	0.00238	0.00289	0.00366	0.003417	0.20567

θπ and θω are measures of nucleotide diversity; Fixation index (F_{ST}) is a measure of population differentiation due to genetic structure

Nature Biotechnology: doi:10.1038/nbt.3943

Supplementary Table 34. Regions with loss of diversity and strong differentiation between wild and cultivated pearl millet

It has been uploaded as an excel spread sheet.

Nature Biotechnology: doi:10.1038/nbt.3943

Supplementary Table 35. List of the genes found in the regions showing strong differentiation between wild and cultivated germplasm and diversity loss in the cultigen

It has been uploaded as an excel spread sheet.

Nature Biotechnology: doi:10.1038/nbt.3943

Supplementary Table 36. Genome-wide marker-trait associations for grain and stover yield

It has been uploaded as an excel spread sheet.

Nature Biotechnology: doi:10.1038/nbt.3943

Supplementary Table 37. Best 170 predicted hybrid combinations

It is provided as separate Excel file

Nature Biotechnology: doi:10.1038/nbt.3943

Supplementary Table 38. Best 11 tested hybrid combinations

It is provided as separate Excel file

Nature Biotechnology: doi:10.1038/nbt.3943

Supplementary Table 39. Pedigree details of B- and R-lines used in the study

It is provided as separate Excel file

Nature Biotechnology: doi:10.1038/nbt.3943

Supplementary Table 40. Details of 345 Pearl Millet Inbred Germplasm Association Panel (PMiGAP) lines used in the study

It is provided as separate Excel file

Nature Biotechnology: doi:10.1038/nbt.3943

Supplementary Table 41. Details of 38 parental lines of mapping populations of pearl millet used in the study

It is provided as separate Excel file

Nature Biotechnology: doi:10.1038/nbt.3943

Supplementary Table 42. Passport data of 31 pearl millet wild species accessions

Accession ID	Country	Year of sampling	Group*
PE05720	Mali	1978	WA
PE05722	Mali	1978	WA
PE05724	Mali	1978	WA
PE08487	Mauritania	1987	WA
PE08498	Mauritania	1987	WA
PE08492	Mauritania	1987	WA
PE08492a	Mauritania	1987	WA
PE08503	Senegal	1987	WA
PE01458	Senegal	1976	WA
PE08726	NA	NA	WA
PE08743	Soudan	1988	WB
PE08744	Soudan	1988	WB
PE08727	Chad	1988	WB
PE08731	Chad	1988	WB
PE08732	Chad	1988	WB
PE08736	Chad	1988	WB
PE08721	Chad	1988	WB
PE08730	Chad	1988	WB
PE08734	Chad	1988	WB
PE08155	Mali	1986	WC
PE08473	Mali	1987	WC
PE08165	Mali	1986	WC
PE08171	Mali	1986	WC
PE08470	Mali	1987	WC
PE00838	Niger	1975	WC
PE08084	Niger	1985	WC
PE08136	Niger	1985	WC
PE08094	Niger	1985	WC
PE08106	Niger	1985	WC
PE08127	Niger	1985	WC
PE08146	Niger	1985	WC

*WA- Western part of Africa covering Senegal, Mauritania and West Mali; WB- Eastern Africa covering Chad and Sudan; WC- Western Central Africa covering East Mali and Niger

Nature Biotechnology: doi:10.1038/nbt.3943
